# Robust differential expression testing for single-cell CRISPR screens at low multiplicity of infection

**DOI:** 10.1186/s13059-024-03254-2

**Published:** 2024-05-17

**Authors:** Timothy Barry, Kaishu Mason, Kathryn Roeder, Eugene Katsevich

**Affiliations:** 1 Department of Biostatistics, Harvard T.H. Chan School of Public Health, Boston, USA; 2 Department of Statistics and Data Science, Wharton School, University of Pennsylvania, Philadelphia, USA; 3 Department of Statistics and Data Science, Carnegie Mellon University, Pittsburgh, USA; 4 Computational Biology Department, Carnegie Mellon University, Pittsburgh, USA

## Abstract

Single-cell CRISPR screens (perturb-seq) link genetic perturbations to phenotypic changes in individual cells. The most fundamental task in perturb-seq analysis is to test for association between a perturbation and a count outcome, such as gene expression. We conduct the first-ever comprehensive benchmarking study of association testing methods for low multiplicity-of-infection (MOI) perturb-seq data, finding that existing methods produce excess false positives. We conduct an extensive empirical investigation of the data, identifying three core analysis challenges: sparsity, confounding, and model misspecification. Finally, we develop an association testing method — SCEPTRE low-MOI — that resolves these analysis challenges and demonstrates improved calibration and power.

**Supplementary Information:**

The online version contains supplementary material available at 10.1186/s13059-024-03254-2.

## Background

Pooled CRISPR screens with single-cell readout (e.g., Perturb-seq [1]) have emerged as a scalable, flexible, and powerful technique for connecting genetic perturbations to molecular phenotypes, with applications ranging from fundamental molecular biology to medical genetics and cancer research [2]. In such screens, a library of genetic perturbations is transfected into a population of cells via CRISPR guide RNAs (gRNAs), followed by single-cell sequencing to identify the perturbations present and measure a rich molecular phenotype for each cell. The perturbations can target either genes [1] or non-coding regulatory elements [3–5], either repressing [1] or activating [6] these targets; the molecular readouts can include gene expression [1], protein expression [7–9], or epigenetic phenotypes like chromatin accessibility [10]. Typically, perturbations are introduced at low multiplicity of infection (MOI), with one perturbation per cell. In cases where perturbations are expected to have weak effects (like regulatory-element-targeting screens), perturbations also can be introduced at high-MOI (with many perturbations per cell) to increase scalability [3–5, 11, 12].

The most fundamental statistical task involved in the analysis of single-cell CRISPR screen data is to test for association between a perturbation and a univariate, count-based molecular phenotype, such as the expression of a gene or protein. In our previous work on high-MOI single-cell CRISPR screen analysis, we discovered that existing methods for association testing are prone to an excess of false positive hits [13]. In that work, we proposed SCEPTRE, a well-calibrated method for association testing on high-MOI data. Since low-MOI screens currently outnumber high-MOI screens, the low-MOI association testing problem is even more pressing. A variety of methods has been deployed for association testing in low-MOI [1, 8–10, 14–17]. However, there is no consensus as to which of these methods represents the “state of the art”; these methods have not undergone rigorous statistical validation and comparison [18], and in fact, there is no commonly accepted framework for quantifying the statistical validity of single-cell CRISPR screen association testing methods. Resolving these fundamental issues is essential to ensuring the reliability of biological conclusions made on the basis of single-cell CRISPR screen experiments.

We aimed to address the aforementioned challenges through three contributions. First, we developed a simple framework for evaluating the calibration of association testing methods for single-cell CRISPR screens. We then leveraged this framework to conduct the first-ever [18] comprehensive benchmarking study of association methods on low-MOI data, applying six leading methods to analyze six diverse datasets. We found that all existing methods exhibit varying degrees of miscalibration, indicating that results obtained using these methods may be contaminated by excess false positive discoveries. Second, to shed light on why existing methods might demonstrate miscalibration, we conducted an in-depth empirical investigation of the data, uncovering three core analysis challenges: confounding, model misspecification, and data sparsity. No existing method addresses all of these analysis challenges, explaining their lack of calibration. Finally, we developed SCEPTRE (low-MOI), a substantial extension of the original SCEPTRE [13] tailored to the analysis of low-MOI single-cell CRISPR screens. SCEPTRE (low-MOI) is based on the novel and statistically principled technique of permuting negative binomial score statistics (we often will refer to the low-MOI version of SCEPTRE simply as “SCEPTRE” for the sake of brevity). SCEPTRE addresses all three core analysis challenges both in theory and in practice, demonstrating markedly improved calibration and power relative to existing methods across datasets. SCEPTRE is available at katsevich-lab.github.io/sceptre/.

## Results

### A survey of leading analysis methods

**Fig. 1:**
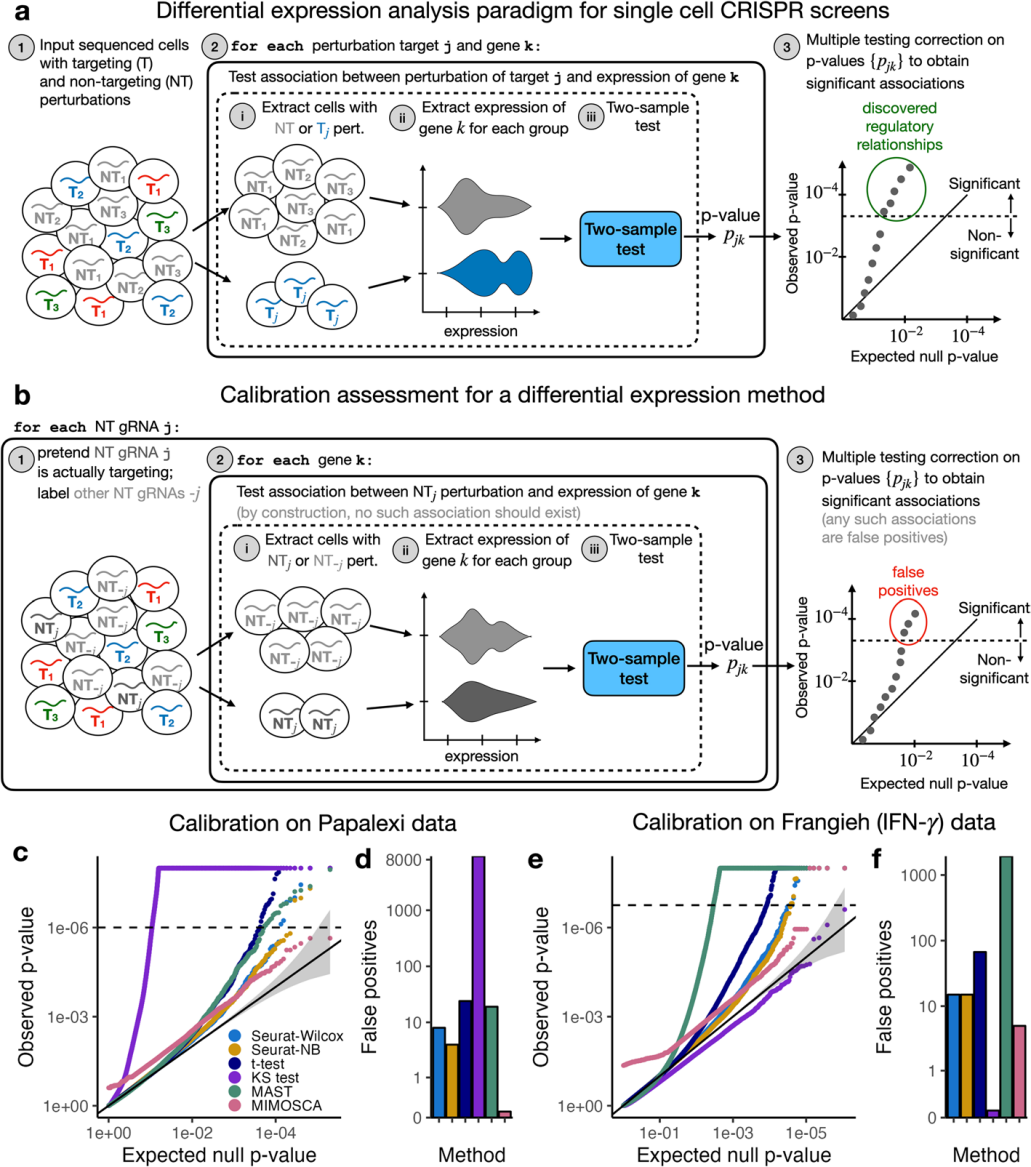
Comprehensive benchmarking study of single-cell CRISPR screen association testing methods on low-MOI data. **a** The standard paradigm for association testing on low-MOI single-cell CRISPR screen data. To test for association between a given targeting perturbation and gene, one tests for differential expression of the gene across two groups of cells: those containing the given targeting perturbation and those containing a non-targeting (NT) perturbation. One typically repeats this procedure for a large, preselected set of targeting-perturbation gene pairs, obtaining a discovery set by subjecting the resulting *p*-values to a multiple comparison correction procedure (e.g., Benjamini-Hochberg). **b** The calibration check paradigm. One constructs “null” or “negative control” perturbation-gene pairs by coupling each individual NT gRNA to the entire set of genes. One then assesses the calibration of a method by deploying the method to analyze these null pairs. Any *p*-values that survive the multiple testing correction procedure correspond to false positive discoveries. **c**, **d** Results of the calibration check benchmarking analysis on the Papalexi gene expression data. **c** QQ plot of the null *p*-values (colored by method) plotted on a negative log transformed scale. Gray region, 95% confidence band. **d** Number of false discoveries that each method makes on the null pairs after a Bonferroni correction at level 0.1. **e**, **f** Similar to panels **c–d**, but for the Frangieh IFN-$\gamma$ data

Association testing on low-MOI single-cell CRISPR screens is a variation on the classical single-cell differential expression testing problem (Fig. 1a). To test for association between a given targeting CRISPR perturbation and gene, one first divides the cells into two groups: those that received the targeting perturbation and those that received a non-targeting (NT) perturbation (all other cells typically are ignored). One then tests for differential expression of the given gene across these two groups of cells, yielding a fold change estimate and *p*-value. One repeats this procedure for a (typically) large, preselected set of perturbation-gene pairs. Finally, one computes the discovery set by subjecting the tested pairs to a multiplicity correction procedure (e.g., Benjamini-Hochberg).

We use the term “control group” to refer to the cells against which the cells that received the targeting perturbation are compared. As indicated above, the control group typically is the set of cells that received an NT perturbation (i.e., the “NT cells”). Certain single-cell CRISPR screen methods, however, take as their control group the set of cells that did *not* receive the targeting perturbation (i.e., the “complement set”). In low-MOI screens, the NT cells generally constitute a more natural control group than the complement set, as we seek to compare the effect of the targeting perturbation to that of a “null” perturbation rather than to the average of the effects of all other perturbations introduced in the pooled screen. In high-MOI screens, however, the complement set is the only choice, because few (if any) cells receive only NT perturbations.

We surveyed recent analyses of single-cell CRISPR screen data and identified five methods commonly in use: the default Seurat [19] FindMarkers() function based on the Wilcoxon test (Seurat-Wilcox), MIMOSCA [1], a *t*-test on the library-size-normalized expressions [10], MAST [20], and a Kolmogorov-Smirnov (KS) test on the library-size-normalized expressions [21]. We also considered applying FindMarkers() with negative binomial (NB) regression rather than the Wilcoxon test (Seurat-NB). These methods vary along several dimensions (Table 1; “the Existing method details” section), including their testing paradigm (two-sample test versus regression-based test), how they normalize the data, whether they make parametric assumptions, and whether they use the NT cells or the complement set as their control group. Most of these methods are popular single-cell differential expression procedures that have been adapted to the single-cell CRISPR screen setting.

**Table 1:** A summary of low-MOI single-cell CRISPR screen DE methods currently in use. The applications of each method to single-cell CRISPR screens are cited below the method name. The methods vary along several key axes, including the use (or lack thereof) of parametric assumptions, the construction of the null distribution, the variables adjusted for, and the control group. NT, non-targeting

Method	Paradigm	Parametric assumption	Null distribution	Normalization/ adjustments	Control group
Seurat-Wilcox [7, 9, 22]	Two-sample test	No	Asymptotic	Library size	NT cells
MIMOSCA [1, 8, 23–26]	Regression-based	No	Permutation	Library size, other covariates	Complement set
*t*-test [10]	Two-sample test	Yes	Asymptotic	Library size	NT cells
MAST [15, 20]	Regression-based	Yes	Asymptotic	Library size, expressed genes	NT cells
KS test [16, 21]	Two-sample test	No	Asymptotic	Library size, batch	NT cells
Seurat-NB (single-cell DE)	Two-sample test	Yes	Asymptotic	Library size	NT cells

### Comprehensive benchmarking study of leading analysis methods

We sought to assess whether these methods are correctly calibrated (i.e., whether they yield uniformly distributed *p*-values under the null hypothesis of no association between the perturbation and gene). Methods that are not correctly calibrated can produce discovery sets that are contaminated by excess false positives or false negatives. Unfortunately, there does not exist a standard protocol for assessing the calibration of single-cell CRISPR screen association methods. The closest existing analysis [15] proceeds by applying methods to analyze gene-perturbations pairs for perturbations with known targets. Any pair where the gene is not the known target of the perturbation is considered null. As acknowledged by the original authors, this approach underestimates precision because downstream effects of perturbations are not taken into account.

To help fill this methodological gap, we designed a simple procedure to ascertain the calibration of a single-cell CRISPR screen association method (Fig. 1b). We constructed a set of “null” or “negative control” perturbation-gene pairs by pairing each NT gRNA to each gene. We then deployed a given method to analyze these null pairs (for methods that use the NT cells as their control group — the majority of methods — this check consists of comparing cells containing a given NT gRNA to cells containing *all other* NT gRNAs). The output of this check is a set of $N_\text {gene} \cdot N_\text {NT}$ null *p*-values, where $N_\text {gene}$ is the number of genes and $N_\text {NT}$ is the number of NT gRNAs. Since the null perturbation-gene pairs are devoid of signal, a well-calibrated association method should output uniformly distributed *p*-values on these pairs. Deviations from uniformity — and thus miscalibration of the method — can be detected by inspecting a QQ plot of the *p*-values. Quantitatively, the number of null pairs passing a Bonferroni correction measures the extent of the miscalibration; well-calibrated methods should have roughly zero such pairs.

We note that there are two uses of the proposed calibration check procedure. The first use is for the goal of benchmarking existing analysis methods to identify which, if any, are suitable for broad application (the primary goal of this section). The second use is for the goal of testing whether a *given* method is well-calibrated on a *given* dataset. These two goals are distinct; a method may not be broadly well-calibrated but may perform adequately on a given dataset. In the context of the second goal, we recommend applying a modified calibration check where the set of negative control perturbation-gene pairs is matched to the set of pairs under consideration based on several criteria (described later).

We employed the above framework to systematically benchmark the performance of the existing methods, implementing each as faithfully as possible in a publicly available R package lowmoi (github.com/katsevich-lab/lowmoi). We applied the calibration check procedure using six single-cell CRISPR screen datasets, five real and one simulated (Additional file 1: Tables S1-S2). The five real datasets came from three papers: Frangieh 2021 [8] (three datasets), Papalexi 2021 [9] (one dataset), and Schraivogel 2020 [15] (one dataset). The data were diverse, varying along the axes of CRISPR modality (CRISPRko or CRISPRi), technology platform (perturb-CITE seq, ECCITE-seq, or targeted perturb-seq), cell type (TIL, K562, or THP1), and genomic element targeted (enhancers or gene TSS). Notably, the Papalexi data were multimodal, containing both gene and protein expression measurements. For simplicity we analyzed the gene and protein modalities separately throughout.

Surprisingly, the results of our analyses (Fig. 1c-f; Additional file 1: Figs. S1, S2, S3) revealed substantial miscalibration for many dataset-method pairs. On the Papalexi data, for example, the KS test produced inflated *p*-values, yielding over 9,000 false Bonferroni discoveries. MAST was similarly inflated on the Frangieh IFN-$\gamma$ data, falsely rejecting nearly 2000 null perturbation-gene pairs. MIMOSCA, meanwhile, exhibited noticeably non-uniform behavior on both datasets, outputting *p*-values strictly less than 0.26 across all pairs. Overall, the two best methods appeared to be Seurat-Wilcox and Seurat-NB, although these two methods still demonstrated clear signs of miscalibration. We noted that the calibration quality of a given method could vary significantly across datasets; this is explained by the fact that different datasets posed different analysis challenges. Nevertheless, we concluded that none of the methods was adequately calibrated across all datasets tested, suggesting that existing methods may not be suitable for broad application to single-cell CRISPR screen data.

### Systematic identification of core analysis challenges

We conducted an extensive empirical investigation of the data to search for possible sources of miscalibration, uncovering three core analysis challenges: sparsity, confounding, and model misspecification. No method that we examined addressed more than one of these analysis challenges (Additional file 1: Table S3), explaining their collective lack of calibration. This section is abbreviated to preserve space; interested readers can consult the “Details of the investigation into the core analysis challenges” section, which contains a more detailed description of this investigation.

Single-cell CRISPR screen data typically are sparse, both in terms of gene expression and perturbation presence. Many genes have nonzero expression in only a small fraction of cells. On the other hand, due to the pooling of a large number of perturbations in a single experiment, the perturbation presence data are also sparse: most perturbations are present in only a small fraction of cells. The latter sparsity distinguishes single-cell CRISPR screens from other single-cell applications and is particularly pronounced in low-MOI. To summarize both sources of sparsity in a single number, we defined the “effective sample size” for a given perturbation-gene pair as the number of cells containing both the perturbation and nonzero gene expression.

**Fig. 2:**
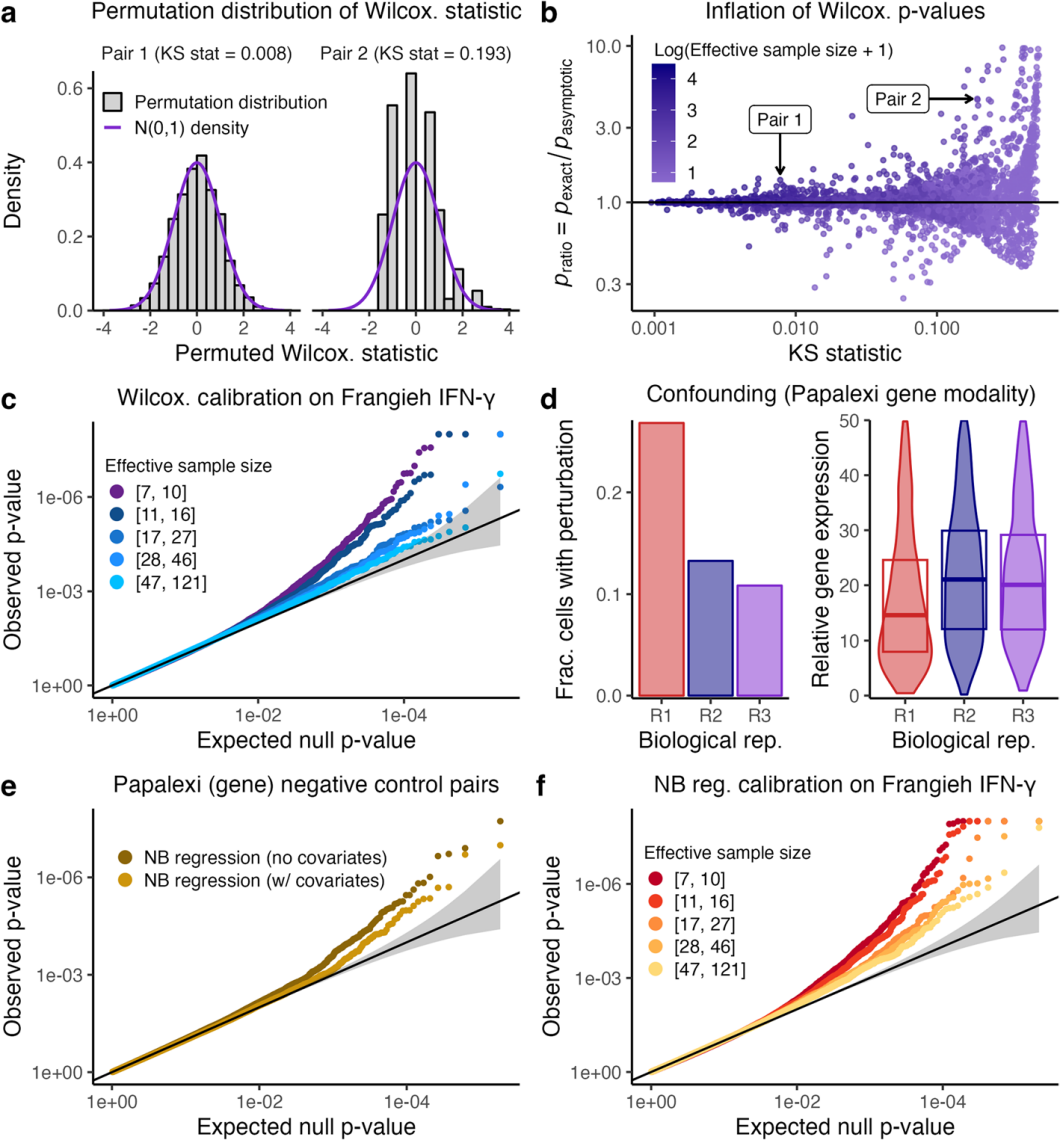
Sparsity, confounding, and model misspecification are core analysis challenges in single-cell CRISPR screen analysis. **a** The exact null distribution of the Wilcoxon test statistic (obtained via permutations; gray) on two pairs from the Frangieh IFN-$\gamma$ data. The Wilcoxon test (and thus Seurat-Wilcox) approximates the exact null distribution using a standard Gaussian density (purple). For pair 1 (left), the Gaussian approximation to the exact null distribution is good (goodness of fit KS statistic = 0.008), while for pair 2 (right) the approximation is inadequate (goodness of fit KS statistic = 0.193). **b** A plot of $p_\text {ratio}$ (defined as the ratio of the exact Wilcoxon *p*-value, $p_\text {exact}$, to the asymptotic Wilcoxon *p*-value, $p_\text {asymptotic}$) vs. goodness of fit of the Gaussian distribution to the exact null distribution (as quantified by the KS statistic). Each point represents a gene-gRNA pair; pairs 1 and 2 (from panel **a**) are annotated. As the KS statistic increases (indicating worse fit of the Gaussian distribution to the exact Wilcoxon null distribution), $p_\text {ratio}$ deviates more from one, indicating miscalibration. Points are colored according to the effective sample size of the corresponding pair. **c** Stratification of the Seurat-Wilcox *p*-values on the Frangieh IFN-$\gamma$ negative control data by effective sample size. **d** An example of confounding on the Papalexi data. Left (resp. right), the fraction of cells that received a given NT gRNA (resp., the relative expression of a given gene) across biological replicates “R1,” “R2,” and “R3.” **e** Application of NB regression with and without covariates to the Papalexi data. **f** Stratification of the NB regression *p*-values on the Papalexi (gene expression) negative control data by effective sample size

We found that effective sample size had a substantial effect on the calibration of many methods under consideration (Additional file 1: Figs. S4-S5), especially those based on asymptotic approximations, such as Seurat-Wilcox. Asymptotic approximations tend to break down when the effective sample size is too low. For example, we compared the exact null distribution of the Wilcoxon test statistic (obtained via permutations) to the asymptotic Gaussian distribution used by Seurat-Wilcox; the latter is a computationally tractable approximation to the former in large samples. The Gaussian distribution provided a reasonable approximation to the exact null distribution for some pairs (Fig. 2a, left) but not others (2a, right). Furthermore, as the effective sample size decreased and the Gaussian approximation degraded in accuracy, the *p*-value obtained via the Gaussian approximation likewise degraded in accuracy (Fig. 2b). Finally, stratifying the Seurat-Wilcox null *p*-values by effective sample size on the Frangieh IFN-$\gamma$ data revealed that pairs with small effective sample sizes yielded more inflated *p*-values than pairs with large effective sample sizes (Fig. 2c).

Second, technical factors, such as biological replicate, batch, and library size, impact not only a cell’s expression level but also its probability of receiving a perturbation, thereby creating a confounding effect that can lead to spurious associations [13]. All existing methods adjust for library size, but few adjust for other technical factors (Table 1). We studied how the variable of biological replicate confounded the Papalexi (gene modality) data (Fig. 2d). The Papalexi data were sequenced across three separate biological replicates (which we labeled “R1,” “R2,” and “R3”). We visually examined the relationship between biological replicate and a given NT gRNA (“NTg4”) and gene (*FTH1*). We plotted the fraction of cells in each biological replicate that harbored “NTg4” (Fig. 2d, left); additionally, we plotted the (library-size-normalized) expression level of *FTH1* across biological replicate, superimposing boxplots indicating the 25th, 50th, and 75th percentiles of the distribution (Fig. 2d, right). We observed clear visual evidence that biological replicate impacted *both* NTg4 presence or absence *and* *FTH1* expression level, creating a confounding effect. For example, cells in biological replicate R1 were much more likely than cells in biological replicates R2 or R3 to contain NTg4; on the other hand, cells in biological replicate R1 exhibited a much lower expression level of *FTH1* than cells in biological replicates R2 or R3. If one naively tested for association between NTg4 and *FTH1* while ignoring batch, one would incorrectly conclude that NTg4 *decreased* the expression of *FTH1*. In fact, NTg4 exerted no effect on the expression *FTH1*, as NTg4 was a negative control gRNA.

To assess the utility of adjusting for technical factors beyond library size, we applied negative binomial (NB) regression — both with and without biological replicate included as a covariate — to the Papalexi negative control data (Fig. 2e). The variant of NB regression with biological replicate, though not perfectly calibrated, outperformed its counterpart without biological replicate. Methods not adjusting for biological replicate on the Papalexi data (such as Seurat-Wilcox) exhibited *worse* calibration for large effective sample sizes (Additional file 1: Figs. S4 and S5), where there is more power to detect the spurious confounding-driven associations.

Third, methods that rely upon parametric models for the gene expression distribution, such as NB regression and MAST, can yield miscalibrated *p*-values when those models are misspecified [27]. To assess this effect, we monitored *p*-value calibration of the NB regression method on the Frangieh IFN-$\gamma$ data while gradually increasing the effective sample size (Fig. 2f). We found that the calibration quality improved until a point before plateauing; even for large effective sample sizes, noticeable miscalibration remained (the non-parametric Seurat-Wilcox method, by contrast, attained good calibration for large effective sample sizes on this dataset). This pattern was consistent with poor fit of the NB regression model, potentially due to inadequate estimation of the NB size parameter.

### SCEPTRE (low-MOI) addresses the analysis challenges

**Fig. 3:**
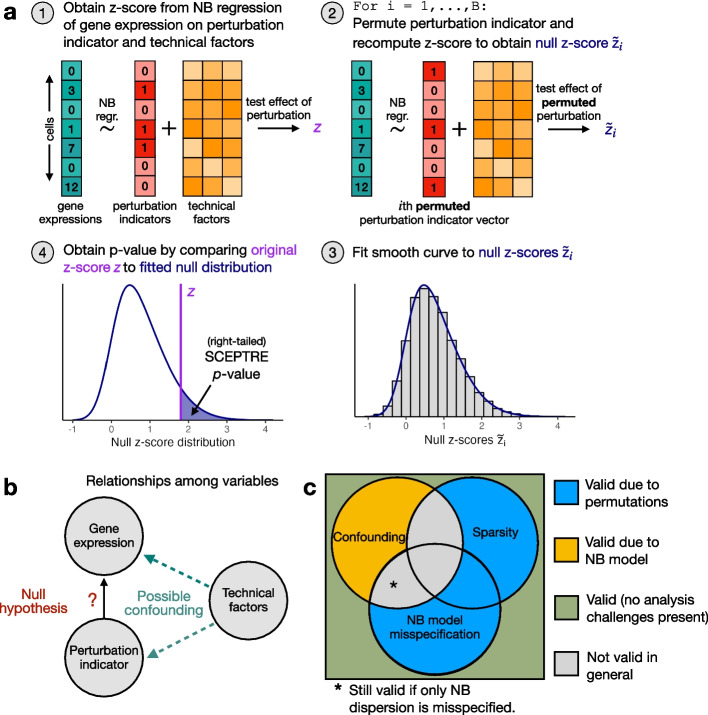
SCEPTRE addresses the core analysis challenges of sparsity, confounding, and model misspecification in theory. **a** The SCEPTRE algorithm. First, the gene expressions are regressed onto the perturbation indicators and technical factors, and the *z*-score $z_\text {obs}$ corresponding to the perturbation indicator is computed. Second, the perturbation indicators are permuted (while the gene expressions and technical factors are held fixed) and the *z*-score is recomputed, yielding *B* “null” *z*-values. Third, a smooth density is fit to the histogram of the null *z*-values. Fourth, a *p*-value is computed by evaluating the tail probability of the fitted density at $z_\text {obs}$. **b** A diagram representing the relationship between the variables in the analysis. The technical factors often (but not always) exert a confounding effect on the perturbation indicator and gene expression. **c** A diagram illustrating the robustness properties of SCEPTRE. The circles represent analysis challenges. A perturbation-gene pair can be affected any subset of the analysis challenges. The color in each region of the diagram indicates whether SCEPTRE is valid on pairs affected by that subset of analysis challenges (blue, yellow, or green = valid; gray = not valid in general). For regions in which SCEPTRE is valid, the color of the region indicates *why* SCEPTRE is valid (yellow = NB model, blue = permutations). The validity of SCEPTRE is overdetermined on pairs unaffected by *any* analysis challenge (green region) due to the combination of the NB model and permutations

We next developed SCEPTRE (low-MOI), a method for robust single-cell CRISPR screen association testing on low-MOI data (Fig. 3a). For a given targeting perturbation-gene pair, SCEPTRE first regresses the vector of gene expressions onto the vector of perturbation indicators and matrix of technical factors via an NB GLM (a given entry of the perturbation indicator vector is set to “1” if the corresponding cell contains a targeting perturbation and “0” if it contains a non-targeting perturbation). SCEPTRE then computes the *z*-score $z_{obs}$ for a test of the null hypothesis that the the coefficient corresponding to the perturbation indicator in the fitted GLM is zero. Next, SCEPTRE permutes the perturbation indicator vector *B* times (while holding fixed the gene expression vector and technical factor matrix) and recomputes a *z*-score for each of the permuted indicator vectors, yielding *B* “null” *z*-scores. Finally, SCEPTRE fits a smooth (skew-normal) density to the histogram of null *z*-scores and computes a *p*-value by evaluating the tail probability of the fitted density based on the original test statistic $z_\text {obs}$.

SCEPTRE possesses several appealing theoretical and computational properties. Theoretically, SCEPTRE is robust to the calibration threats of sparsity, confounding, and model misspecification. A key observation is that the technical factors (e.g., biological replicate) may or may not exert a confounding effect on the perturbation indicator and gene expression (Fig. 3b). If confounding is absent for a given perturbation-gene pair, then SCEPTRE is valid even when the NB model is misspecified or the problem is highly sparse. On the other hand, if confounding is present, then SCEPTRE retains validity if the NB model is correctly specified and the problem is not too sparse (Fig. 3c) (in fact, in the latter case, empirical results indicate that the NB model need only be specified correctly up to the dispersion parameter, sidestepping the difficult problem of NB dispersion parameter estimation [28, 29]). In this sense, SCEPTRE is the only method that addresses all three core analysis challenges (Additional file 1: Table S3). We empirically demonstrated the above key robustness property of SCEPTRE in simulation experiments (Additional file 1: Figs. S8 and S11).

SCEPTRE also is performant, capable of analyzing hundreds of perturbation-gene pairs per second. We attained this efficiency by implementing several computational accelerations. First, we elected to use a score test (as opposed to a more standard Wald or likelihood ratio test) to compute the NB *z*-scores; the score test enabled us to fit a single NB GLM per perturbation-gene pair and share this fitted GLM across all permuted perturbation indicator vectors. Second, we derived a new algorithm for computing GLM score tests, which is hundreds of times faster than the classical algorithm when the perturbation indicator vector is sparse, as is often the case in single-cell CRISPR screen analysis (Additional file 1: the “Comparing the spectral decomposition algorithm to the QR decomposition algorithm for computing GLM score tests” section). Finally, we developed a novel strategy — “inductive without replacement sampling” — for recycling compute across permutation tests in which each test contains the same number of control units (Additional file 1: the “Inductive without replacement sampling” section).

A natural question is whether SCEPTRE is better (in some sense) than the simpler method that entails “regressing out” the technical factors via an NB GLM, extracting the residuals from the fitted GLM, and then performing a permutation test on the residuals (taking, for example, the difference in means across treatment and control groups as the test statistic). We answered this question in the affirmative, finding that SCEPTRE was considerably more powerful than the alternative, residual-based method on both real and simulated data (Additional file 1: the “Comparing the score statistic to the difference-in-residual-means statistic” section). Finally, we note that SCEPTRE (low-MOI) is inspired by, but distinct in several ways from SCEPTRE (high-MOI) [13]. We clarify similarities and differences between these two methods in the “Comparison of SCEPTRE (low-MOI) and SCEPTRE (high-MOI)” section.

### Application of SCEPTRE to negative and positive control data

**Fig. 4:**
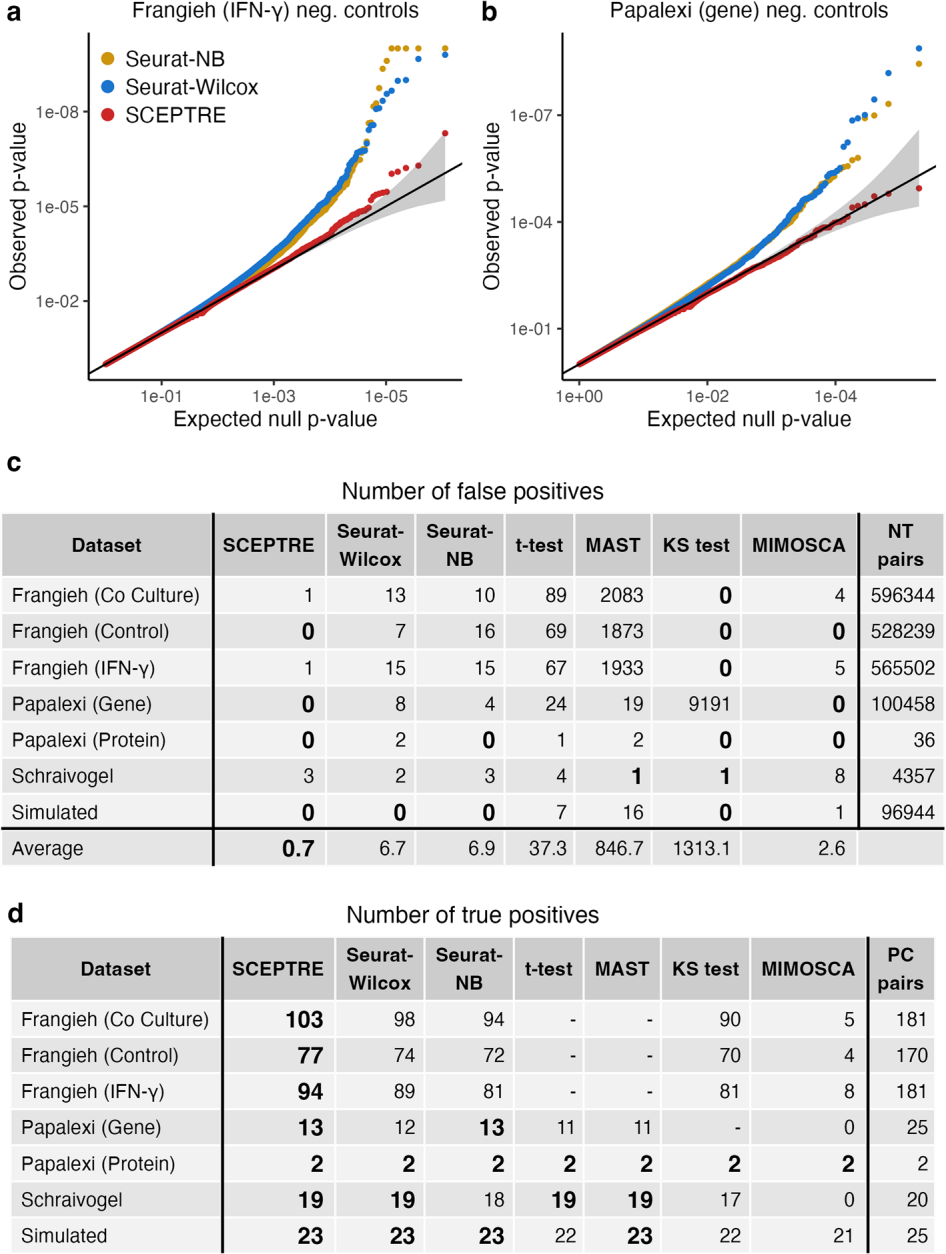
SCEPTRE demonstrates improved calibration and power relative to existing methods across datasets. **a** (resp. **b**) QQ plot of the *p*-values outputted by Seurat-NB, Seurat-Wilcox, and SCEPTRE on the Frangieh IFN-$\gamma$ (resp., Papalexi gene expression) negative control data. Gray band, 95% confidence region. **c** Number of false discoveries (at Bonferroni correction level 0.1) on the negative control data for each method-dataset pair. **d** Number of true discoveries (significant at level $\alpha = 10^{-5}$) on the positive control data for each each method-dataset pair

We included SCEPTRE in the calibration benchmarking analysis presented before. An inspection of the QQ plots revealed that SCEPTRE markedly improved on the calibration of the two best existing methods, namely Seurat-Wilcox and Seurat-NB (Fig. 4a, b). For example, on the Frangieh IFN-$\gamma$ data, SCEPTRE made one Bonferroni rejection and yielded *p*-values that lay mostly within the gray 95% confidence band. The Seurat methods, by contrast, made fifteen false rejections each and produced *p*-values that fell considerably outside the confidence band. Next, we tabulated the number of Bonferroni-significant false positives for each dataset-method pair (Fig. 4c; smaller values are better). SCEPTRE generally made the fewest number of false discoveries among all methods. On average over datasets, SCEPTRE made only 0.7 false discoveries, a roughly tenfold improvement over the Seurat methods.

Next, we assessed the power of the methods by applying them to positive control data. We constructed positive control pairs for each dataset by coupling perturbations targeting TSSs to the genes (or proteins) regulated by these TSSs. We examined the number of “highly significant” discoveries — operationally defined as rejections made at level $\alpha = 10^{-5}$ — made by each method on each dataset (Fig. 4d; larger values are better). Methods that exhibited extreme miscalibration on a given dataset (defined as $>50$ Bonferroni rejections on the negative control pairs of that dataset) were excluded from the positive control analysis, as assessing the power of such methods is challenging. We found that SCEPTRE matched or outperformed the other methods with respect to power on every dataset (while at the same time achieving better calibration on negative control data).

### Pairwise quality control and experimental design

Quality control (QC) — the removal of low-quality cells — is a key step in the analysis of single-cell data. In the context of single-cell CRISPR screens, it is useful not only only to remove low-quality cells but also low-quality perturbation-gene pairs. We term this latter type of QC “pairwise QC.” As discussed previously, effective sample size — the number of cells containing both the perturbation and nonzero gene expression — affects the calibration of several methods considered. It also affects power, as small effective sample sizes yield low power and therefore needlessly increase the multiplicity burden. We found that SCEPTRE rarely rejected positive control pairs with an effective sample size below seven (Additional file 1: Fig. S9); moreover, SCEPTRE maintained calibration for negative control pairs with an effective sample size of seven and above (Additional file 1: Figs. S4 and S5). For this reason, our pairwise QC strategy consisted of filtering for pairs with an effective sample size of seven or greater. We applied this pairwise QC throughout.

Additionally, we reasoned that our results on the power and calibration of SCEPTRE might inform questions related to the experimental design of single-cell CRISPR screens, such as the number of cells per perturbation required to ensure adequate power and calibration of SCEPTRE on a given dataset. We derived a simple mathematical expression for the minimum number of cells that must contain each perturbation so as to ensure that a specified fraction of pairs passes pairwise QC, where the pairwise QC threshold was selected on the basis of SCEPTRE’s calibration and power at different effective sample sizes. We concluded that, on a dataset of standard sparsity (e.g., the Frangieh IFN-$\gamma$ dataset or the Papalexi gene modality dataset), each perturbation should be contained within at least 50–65 cells to ensure that $95\%$ of pairs pass pairwise QC (see Additional file 1: the “Experimental design considerations” section for more details).

### Application of SCEPTRE for discovery analyses

**Fig. 5:**
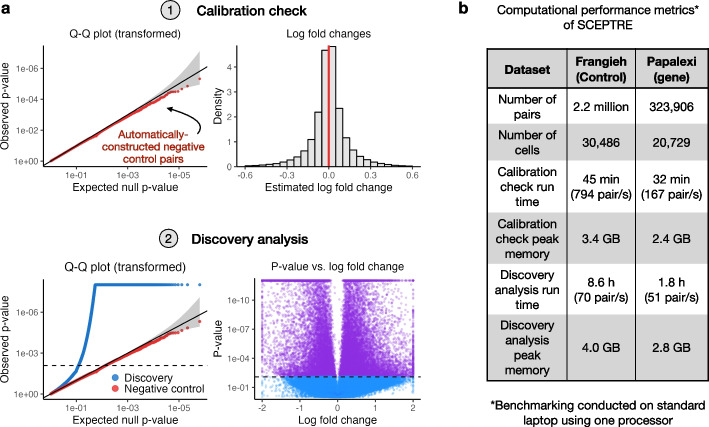
Applying SCEPTRE to make biological discoveries. **a** The standard workflow involved in applying SCEPTRE to a new dataset, using the Papalexi gene expression data as a running example. First, SCEPTRE is applied to analyze a set of automatically constructed negative control pairs (the “calibration check”). The resulting negative control *p*-values are plotted on a QQ plot to ensure uniformity (upper left), and the negative control log-fold changes are plotted on a histogram to ensure symmetry about zero (upper right). Second, SCEPTRE is applied to analyze the discovery pairs (the “discovery analysis”). The discovery *p*-values are superimposed over the negative control *p*-values to ensure that signal is present in the discovery set (lower left), and a volcano plot is created (lower right). **b** Computational performance metrics of SCEPTRE on the Frangieh (control) and Papalexi (gene expression) data. A complete *trans* analysis was conducted on both datasets. Several metrics are reported, including calibration check run time, calibration check peak memory usage, discovery analysis run time, and discovery analysis peak memory usage

The standard workflow involved in applying SCEPTRE to analyze a new single-cell CRISPR screen dataset consists of three main steps. First, the user prepares the data to pass to SCEPTRE and defines the “discovery set,” which is the set of perturbation-gene pairs that the user seeks to test for association (a reasonable default choice is the set of all possible pairs). Second, the user runs the “calibration check” to verify that SCEPTRE is adequately calibrated on the dataset under analysis. The calibration check involves applying SCEPTRE to analyze a set of automatically constructed negative control pairs. These negative control pairs are “matched” to the discovery pairs in several respects. For example, the negative control pairs and discovery pairs are subjected to the exact same pairwise QC, and the number of negative control pairs is set equal to the number of discovery pairs. If the calibration check fails, the user can take steps to improve calibration, such as adding covariates or varying the QC thresholds. After verifying adequate calibration, the user runs the “discovery analysis,” which entails applying SCEPTRE to analyze the pairs contained in the discovery set (Fig. 5a).

To illustrate the above workflow, we applied SCEPTRE to carry out a complete *trans* analysis of the Papalexi (gene expression) and Frangieh (control) datasets. Many of the genes targeted for knockout in these datasets were transcription factors (TFs); thus, our main biological objective was to map the TFs to their target genes. We carried out a calibration check and discovery analysis on both datasets (Fig. 5b). These fairly large analyses completed within a matter of hours on a single laptop processor and required a few gigabytes of memory.

To validate the results of the discovery analysis, we identified the downstream target genes of the transcription factors STAT1 and IRF1 using cell-type-relevant ChIP-seq data [30]. We examined the degree of concordance between the discovery set produced by each method and the ChIP-seq-identified targets (Additional file 1: Fig. S7). To this end, for each method, we constructed a two-by-two contingency table of genes contained within the discovery set of the method and genes whose TSS overlapped with a ChIP-seq peak. We computed a *p*-value (via a Fisher exact test) on this contingency table, quantifying the extent to which the method’s discovery set was enriched for ChIP-seq signal.

We made several observations. First, the discovery set of the KS test did not demonstrate enrichment for ChIP-seq signal (enrichment $p > 0.5$), likely because the KS test produced a large number of false discoveries. Second, and somewhat surprisingly, MIMOSCA’s discovery set exhibited the greatest degree of enrichment for ChIP-seq signal (among all methods) on STAT1 and the lowest degree of enrichment (among all methods, excluding the KS test) on IRF1. However, MIMOSCA made many fewer discoveries than any other method on the discovery data (in particular, 956 and 875 fewer discoveries than the next closest method for IRF1 and STAT1), a trend consistent with MIMOSCA’s results on the the positive control data^1^ (Fig. 4). Among the methods that made a large number of discoveries on both the positive control data and the discovery data (i.e., all methods except for MIMOSCA), SCEPTRE ranked second out of six for ChIP-seq signal enrichment on both IRF1 and STAT1, with the top method being different across the two transcription factors. Thus, SCEPTRE appeared to exhibit consistently good performance on the discovery data (and more broadly across all of our analyses; Fig. 4), increasing our confidence in the results.

## Discussion

Single-cell CRISPR screens have emerged as a powerful method for linking genetic perturbations to rich phenotypic profiles in individual cells. Although poised to impact a variety of research areas, single-cell CRISPR screens will play an especially important role in dissecting the regulatory logic of the noncoding genome. The bulk of genetic risk for diseases lies in noncoding regions, implicating dysregulation of gene expression [31–33]. A major challenge in genetics, therefore, is to map noncoding disease variants to the genes that they target, target genes to the molecular programs that they regulate, and — ultimately — molecular programs to disease [34]. Single-cell screens have enabled breakthrough progress on these tasks. For example, two recent studies leveraged high-MOI single-cell screens to perturb blood disease [11] and cancer [35] GWAS variants (in some cases at single nucleotide resolution) and link these variants to target genes in disease-relevant cell types (both studies used SCEPTRE (high-MOI) to analyze their data). Another recent study leveraged low-MOI single-cell screens to knock down genes regulated by heart disease GWAS variants and map these genes to downstream molecular programs [34]. Given the promise that single-cell screens have demonstrated in understanding noncoding variation, a wave of single-cell screens aiming to link noncoding variants to genes and genes to molecular programs likely will emerge over the coming decade.

It is therefore crucial that reliable methods for single-cell CRISPR screen data analysis be made available. The broad objective of this work was to put single-cell CRISPR screen analysis onto a more solid statistical foundation. To this end, we devised a simple framework for assessing the calibration and power of competing methods; applied this framework to conduct the first-ever comprehensive benchmarking study of existing methods; identified core statistical challenges that the data pose; and developed a method, SCEPTRE, that combines careful modeling with a resampling framework to produce a well-calibrated, powerful, fast, and memory-efficient test of association. Taken together, these contributions help bring statistical rigor to single-cell CRISPR screen data analysis. Furthermore, given the appealing theoretical properties and empirical performance of the proposed method, we anticipate that the method could be extended (with some modification) to applications beyond single-cell CRISPR screens, such as single-cell eQTL analysis and single-cell case-control differential expression analysis [36].

We identified sparsity, confounding, and model misspecification as key challenges in single-cell CRISPR screen analysis. However, the data pose additional challenges that SCEPTRE does not currently address. First, some NT gRNAs may have off-targeting effects. In such cases, testing for association by comparing cells that contain a targeting perturbation to those that contain an NT perturbation could result in a loss of error control. At least one prior work has attempted to resolve this problem [37]. Second, some targeting gRNAs are ineffective, i.e., they fail to perturb their target. Including such defective gRNAs in the analysis can result in a loss of power. Several methods, including MIMOSCA [1], MUSIC [38], and Mixscape [9], have been developed to address this issue. Third, it is challenging to distinguish between direct and indirect effects in the sense that perturbations can be associated with their direct targets or with targets further downstream. Disentangling direct from indirect effects likely admits a statistical solution, but to our knowledge, this problem remains unaddressed. Finally, genes often are co-expressed in “gene modules.” An exciting opportunity is to pool information across genes within the same module to increase the power of perturbation-to-gene association tests; the recent method GSFA does this in a Bayesian framework [39].

## Conclusions

Single-cell CRISPR screens are a promising technology for functional genomics discovery. However, the analysis of single-cell CRISPR screen data presents several statistical and computational challenges, demanding the development of new analytic methods. The SCEPTRE toolkit, which now supports both low- and high-MOI CRISPR screens, provides practitioners with a unified solution for statistically reliable and computationally efficient single-cell CRISPR screen differential expression analysis.

## Methods

### Dataset details

We downloaded, processed, and harmonized five single-cell CRISPR screen datasets (Additional file 1: Table S1), inheriting several data-related analysis decisions made by the original authors. First, we used the gRNA-to-cell assignments that the original authors used, thereby circumventing the need to assign gRNAs to cells using gRNA UMI and/or read count matrices. Papalexi and Schraivogel employed a simple strategy for this purpose: Papalexi identified the gRNA with the greatest UMI count in a given cell and assigned that gRNA to the cell, while Schraivogel assigned gRNAs by thresholding gRNA UMI counts. Frangieh, meanwhile, assigned gRNAs to cells via a more complex approach involving a separate dial-out PCR procedure. We found the gRNA-to-cell assignments adequate and thus used them without modification. Next, we inherited the cell-wise QC that the original authors implemented. For example, Papalexi removed likely duplets (as determined by the Seurat function MULTIseqDemux [40, 41]) as well as cells with excessive mitochondrial content and low gene expression.

We generated a synthetic, negative control single-cell CRISPR screen dataset to use for benchmarking the calibration of the competing methods. The synthetic dataset contained 5000 genes, 25 gRNAs, and 10,000 cells. We generated the matrix of gene expressions by sampling counts from a negative binomial distribution, allowing each gene to have its own mean and size parameter (we drew gene-wise means and sizes i.i.d. from a $\text {Gamma}(0.5, 2)$ distribution and a $\text {Unif}(1, 25)$ distribution, respectively). We randomly inserted gRNAs into cells such that the expected number of cells per gRNA was equal across gRNAs. The dataset was entirely devoid of signal and confounding: no gRNA affected the expression of any gene, and no technical factors impacted the gRNA assignments or gene expressions. We also generated a synthetic positive control dataset to assess the power of the competing methods under known ground truth. The synthetic positive control dataset contained 125 genes, 25 positive control gRNAs, 100 negative control gRNAs, and 15,000 cells. The mean expression of each gene across “treatment” and “control” groups was drawn (separately) from a $\text {Gamma}(0.5, 2)$ distribution. The gene-wise sizes were drawn from a $\text {Unif}(1, 25)$ distribution. Like the negative control dataset, the positive control dataset was devoid of confounding.

We applied our own minimal gene-wise, gRNA-wise, and cell-wise QC uniformly to the datasets. We filtered for genes expressed in at least 0.005 of cells, gRNAs expressed in at least 10 cells, and cells with exactly one gRNA, respectively. Additional file 1: Table S2 summarizes the statistical attributes (e.g., number of genes, number of cells, etc.) of each dataset. Finally, we obtained the set of cell-specific covariates (or technical factors) for each dataset, which we list below. Frangieh co-culture, control, and IFN-$\gamma$ datasets: number of gene UMIs, number of genes expressed; Papalexi (gene modality): number of gene UMIs, number of genes expressed, biological replicate, and percent of gene transcripts that mapped to mitochondrial genes; Papalexi (protein modality): number of protein UMIs, biological replicate, and percent of gene transcripts that mapped to mitochondrial genes; Schraivogel: number of gene UMIs, number of genes expressed, sequencing lane.

### Existing method details

We benchmarked the performance of six methods: Seurat-Wilcox, Seurat-NB, *t*-test, MAST, KS test, and MIMOSCA. The first five of these methods are generic single-cell differential expression methods that have been adapted to single-cell CRISPR screens (either by us or other single-cell researchers), while MIMOSCA is specific to single-cell screens. To facilitate benchmarking of the methods, we implemented all in an R package lowmoi (github.com/Katsevich-Lab/lowmoi). We implemented Seurat-Wilcox and Seurat-NB via a call to the Seurat FindMarkers() function. In the case of Seurat-Wilcox, we called NormalizeData() before FindMarkers() to normalize the gene expressions by dividing the gene expressions by library size. Next, we implemented the *t*-test via a call to t.test() in R. Following Liscovitch et al. [10], we normalized the gene expression vector for a given gene-perturbation pair by dividing by the library size, subtracting the mean, and dividing by the standard deviation. We used the implementation of MAST that Schraivogel et al. used to analyze their single-cell screen data [15]. To this end, we copied and pasted relevant portions of the Schraivogel et al. Github codebase (github.com/argschwind/TAPseq_manuscript) into lowmoi. Similarly, we used the implementation of the KS test that Replogle et al. used to analyze their single-cell screen data [16], again copying and pasting relevant portions of the corresponding codebase into lowmoi (github.com/thomasmaxwellnorman/Perturbseq_GI). Finally, we implemented MIMOSCA by copying and pasting relevant sections of the MIMOSCA package (github.com/klarman-cell-observatory/Perturb-CITE-seq) into lowmoi. Replogle et al.’s implementation of the KS test and MIMOSCA both were written in Python. Thus, we used the reticulate package to access these methods from within R. To ensure consistency of the API across methods, we implemented the methods in such a way that each took the same inputs and returned the same output. Finally, to ensure correctness, we tested for agreement between the output of our implementations and those of the original methods (when possible).

Some methods have an internal QC step in which gene-perturbation pairs that are unpromising or low-quality (as determined by the method itself) are removed. For example, Seurat DE by default filters out gene-perturbation pairs for which the log-fold change of the expression of the gene (across the treatment and control cells) falls below a certain threshold. We disabled such method-specific pairwise QC, allowing us to apply competing methods to the exact same set of gene-perturbation pairs on each dataset, facilitating head-to-head comparisons across methods.

We applied several variants of NB regression to the data. First, as described above, we applied Seurat-NB the negative control and positive control pairs of all datasets. Furthermore, as part of our investigation into the analysis challenges (the “Systematic identification of core analysis challenges” section), we applied NB regression as implemented by the MASS [42] package to the Papalexi (gene expression) and Frangieh IFN-$\gamma$ negative control data (these results are depicted in Fig. 2e–f). We used the MASS implementation of NB regression in exploring the analysis challenges, as MASS is slightly more flexible than Seurat, in particular enabling the straightforward inclusion of covariates. Within the context of MASS NB regression, we tested for association between a perturbation and the expression of a gene via a GLM score test, as implemented by the statmod [43] package. We elected to use a score test (as opposed to a more standard Wald or likelihood ratio test) test to make our implementation of NB regression more comparable to SCEPTRE, as SCEPTRE uses a permutation test built upon an NB regression score test statistic.

### Details of the calibration check procedure

We describe the calibration check procedure in greater detail. Suppose there are *d* distinct NT gRNAs; index these gRNAs from 1 to *d*. Let $\mathcal {C}_1$ denote the set of cells containing NT gRNA 1, $\mathcal {C}_2$ the set of cells containing NT gRNA 2, etc. Let $\mathcal {C} = \mathcal {C}_1 \cup \mathcal {C}_2 \cup \dots \cup \mathcal {C}_d$ denote the set of cells containing *any* NT gRNA (i.e., the “NT cells”). Next, let $\mathcal {C} \setminus \mathcal {C}_i$ denote the set of cells containing *any* NT gRNA *excluding* NT gRNA *i*. Additionally, let $\mathcal {T}$ denote the set of cells containing *any targeting* gRNA (observe that $\mathcal {T} \cup \mathcal {C}$ is the set of all cells). Finally, let $\mathcal {T} \cup \mathcal {C} \setminus \mathcal {C}_i$ denote the set of all cells excluding the cells that contain NT gRNA *i*. Let there be *p* distinct genes.

Suppose we seek to check the calibration of a given method. The way in which we deploy the method to analyze a given negative control pair depends on whether the method uses the NT cells or the complement set as its control group (Table 1). Consider the negative control pair formed by coupling NT gRNA *i* to gene *j*. If the method uses the NT cells as its control group (e.g., Seurat-Wilcox, Seurat-NB, SCEPTRE, etc.), then we apply the method to test for differential expression of gene *j* across the groups of cells $\mathcal {C}_i$ and $\mathcal {C}\setminus \mathcal {C}_i.$ By contrast, if the method uses the complement set as its control group (e.g., MIMOSCA), then we apply the method to test for differential expression of gene *j* across the groups of cells $\mathcal {T} \cup \mathcal {C} \setminus \mathcal {C}_i$ and $\mathcal {C}_i$. The *effective sample size* of the given negative control pair is the number of cells in the set $\mathcal {C}_i$ for which the expression of gene *j* is nonzero. In carrying out our benchmarking analysis (Fig. 1c, f, Fig. 4), we restricted our attention to the subset of the $d \cdot p$ possible negative control pairs whose effective sample size was greater than or equal to seven.

For testing calibration on a given input dataset, the SCEPTRE software automatically constructs a set of negative control pairs that is matched to the pairs in the “discovery set” — i.e., the set of targeting perturbation-gene pairs that the user seeks to test for association — in several respects. First, the negative control pairs and discovery pairs are subjected to the same pairwise QC. Second, the number of negative control pairs is set equal to the number of discovery pairs (assuming the number of possible negative control pairs matches or exceeds the number of discovery pairs). Third, if the user elects to “group” together gRNAs that target the same site (as opposed to running an analysis in which singleton gRNAs are tested for significance), then the negative control pairs likewise are constructed by “grouping” together individual NT gRNAs. Overall, the negative control pairs are designed to mirror the discovery pairs, the difference being that the negative control pairs are devoid of biological signal.

### Details of the investigation into the core analysis challenges

We describe in greater detail our empirical investigations into the core analysis challenges of sparsity, confounding, and model misspecification (as described in the “Systematic identification of core analysis challenges” section).

#### Sparsity

To explore the impact of sparsity on calibration, we deployed the two-sample Wilcoxon test to a randomly selected subset of 5,400 negative control gene-gRNA pairs from the Frangieh IFN-$\gamma$ data (the pairs were selected such that each had an effective sample size of one or greater). Following Seurat-Wilcox, we deployed the Wilcoxon test as follows: first, we normalized the gene expressions by dividing the raw counts by the cell-specific library sizes; then, we applied the Wilcoxon test (as implemented by the wilcox.test function from the stats package in R) to the normalized data, comparing the treatment cells to the control cells. Finally, we computed the Wilcoxon *p*-value in two ways. First, we calculated the asymptotic *p*-value $p_\text {asymptotic}$ by comparing the Wilcoxon test statistic to the standard Gaussian distribution. This approach implicitly assumes that the number of cells with nonzero expression (across both groups) is large enough for the null distribution of the Wilcoxon test statistic to be approximately Gaussian. Next, we calculated the exact *p*-value $p_\text {exact}$ by (i) computing the Wilcoxon statistic on the original data; (ii) permuting the gRNA indicator vector $B = 200,000$ times (while holding fixed the vector of normalized gene expressions), resulting in *B* permuted datasets; (iii) computing the Wilcoxon test statistic on each of these *B* permuted datasets, yielding a permutation (or “null”) distribution of Wilcoxon statistics; and then (iv) calculating the *p*-value $p_\text {exact}$ by comparing the original Wilcoxon statistic to the null Wilcoxon statistics [44]. The latter approach, though computationally expensive (due to the slowness of computing the Wilcoxon statistic), yields a much more accurate *p*-value than the asymptotic approach for lowly expressed genes. Seurat-Wilcox returns the asymptotic *p*-value $p_\text {asymptotic}$ instead of the exact *p*-value $p_\text {exact}$ in virtually all cases^2^.

To study the impact of making the above approximation, we plotted the asymptotic null distribution of the Wilcoxon statistic (i.e., the standard Gaussian distribution) superimposed on top of the exact null distribution of the Wilcoxon statistic (i.e., the permutation distribution) for two pairs from the Frangieh IFN-$\gamma$ negative control data (Fig. 2a). The asymptotic and exact distributions must be highly similar for the asymptotic *p*-value $p_\text {asymptotic}$ to be accurate. We measured goodness of fit of the Gaussian distribution to the exact null distribution by calculating the Kolmogorov-Smirnov (KS) statistic; this statistic ranges from zero to one, with smaller values indicating better fit of the Gaussian distribution to the exact null distribution. We reported the KS statistic for both example pairs in the panels of the plot.

Next, we calculated $p_\text {ratio}$, defined as the ratio of the exact *p*-value $p_\text {exact}$ to the asymptotic *p*-value $p_\text {asymptotic}$, for each of the the 5,400 negative control pairs sampled from the Frangieh IFN-$\gamma$ data. A $p_\text {ratio}$ value of one indicates that the asymptotic and exact *p*-values coincide; a $p_\text {ratio}$ value of greater than one (resp., less than one), on the other hand, indicates inflation (resp., deflation) of the asymptotic *p*-value relative to the exact *p*-value. We sought to explore visually how a small effective sample sizes lead to degradation of the Gaussian approximation, thereby resulting in *p*-value miscalibration (as reflected by $p_\text {ratio}$ values that deviate from one). To this end, we plotted $p_\text {ratio}$ versus goodness of fit of the the Gaussian distribution to the exact null distribution (as quantified by the KS statistic) for each pair (Fig. 2b). We colored the points according to their effective sample size. Pairs 1 and 2 from Fig. 2a were annotated in Fig. 2b.

Finally, to directly assess the impact of sparsity on calibration, we applied Seurat-Wilcox to the IFN-$\gamma$ negative control data, binning the pairs into five categories based on their effective sample size. The bins were defined by effective sample sizes in the ranges [7,10], [11,16], [17,27], [28,46], and [47,121]. The bins were constructed such that an approximately equal number of pairs would fall into each bin. We observed that as the effective sample size increased, the Seurat-Wilcox *p*-values converged to uniformity, illustrating that sparsity is a cause of the miscalibration of Seurat-Wilcox.

#### Confounding

We first explored how the variable of biological replicate confounded the Papalexi (gene modality) data. The Papalexi data were generated and sequenced across three independent experimental replicates, which we labeled “R1,” “R2,” and “R3” (the original data contained a fourth biological replicate as well, but this replicate was removed by the original authors, as it was deemed to be of low quality). We explored the relationship between biological replicate and a given NT gRNA (“NTg4”) and a given gene (*FTH1*). We plotted the fraction of cells in each biological replicate that received the NT gRNA (Fig. 2d, left); additionally, we created a violin plot of the relative expression of the gene across biological replicate (the relative expression $r_i$ of the gene in cell *i* was defined as $r_i = 1000 \cdot \log \left( u_i/l_i + 1 \right) ,$ where $u_i$ was the UMI count of the gene in cell *i*, and $l_i$ was the library size of cell *i*. The violin plots were truncated at a relative expression level of 50). We superimposed boxplots indicating the 25th, 50th, and 75th percentiles of the empirical relative expression distributions on top of the violin plots (Fig. 2d, right). We observed clear visual evidence that biological replicate impacted both NTg4 and *FTH1*, creating a confounding effect.

Next, we extended the above analysis to investigate the entire set of NT gRNAs and genes. First, we tested for association between each NT gRNA and biological replicate. To this end, we constructed a contingency table of gRNA presences and absences across biological replicate, testing for significance of the contingency table using a using a Fisher exact test (as implemented in the R function fisher.test). Next, we tested for association between the relative expression of each gene and biological replicate. To do so, we fit two NB regression models to each gene; the first contained only library size as a covariate, while the second contained both library size *and* biological replicate as covariates. We compared these two models via a likelihood ratio test, yielding a *p*-value for the test of association between relative gene expression and biological replicate. Finally, we created QQ plots of the resulting *p*-values (Fig. S6; gRNA *p*-values, left; gene *p*-values, right). An inflation of the *p*-values across modalities suggested that the bulk of gene-NT gRNA pairs was confounded by biological replicate.

Finally, we directly assessed the impact of adjusting for biological replicate (alongside other potential confounders) by applying two variants of NB regression to the Papalexi (gene modality) negative control data: (i) NB regression with library size (only) included as a covariate and (ii) NB regression with library size as well as all potential confounders (including biological replicate) included as covariates. We plotted the negative control *p*-values on a QQ plot (Fig. 2e). The variant of NB regression with confounders included as covariates exhibited superior calibration, demonstrating that confounding is an analysis challenge. To reduce the effect of sparsity (i.e., the first analysis challenge), we restricted our attention in this plot to gene-gRNA pairs with an effective sample size greater than 10.

#### Model misspecification

To explore the analysis challenge of model misspecification, we applied NB regression to the Frangieh IFN-$\gamma$ negative control data. As in Fig. 2c (in which we applied Seurat-Wilcox to the Frangieh IFN-$\gamma$ negative control data), we partitioned the pairs into five categories based on the effective sample size of each pair. As the number of nonzero treatment cells increased, the NB regression *p*-values failed to converge to uniformity (in contrast to the Seurat-Wilcox *p*-values). The key difference between Seurat-Wilcox and NB regression is that the former is a nonparametric method while the latter is parametric method. Thus, we reasoned that miscalibration of the NB regression *p*-values likely was due to misspecification of the NB regression model (we note that miscalibration of the NB regression *p*-values likely was not due to confounding, as Seurat-Wilcox, which does not adjust for confounding, was well-calibrated for pairs with high expression levels).

### SCEPTRE (low-MOI) overview

Consider a given gene and perturbation. We call the cells that contain the targeting perturbation the “treatment cells” and those that contain an NT perturbation the “control cells.” Suppose there are *n* cells across treatment and control groups. Let $Y = [Y_1, \dots , Y_n]^T$ be the vector of raw gene (or protein) expressions, and let $X = [X_1, \dots , X_n]^T$ be the vector of perturbation indicators, where an entry of one (resp., zero) indicates presence of the targeting (resp. NT) perturbation. Finally, for cell $i \in \{1, \dots , n\},$ let $Z_i$ be the *p*-dimensional vector of technical factors for cell *i* (containing library size, batch, etc.). We include an entry of one in each $Z_i$ to serve as an intercept term. Let *Z* be the $n \times p$ matrix formed by concatenating the $Z_i$s, and let [*X*, *Z*] be the $n \times (p+1)$ matrix formed by concatenating *X* and *Z*.

We model $Y_i$ as a function of $X_i$ and $Z_i$ via an NB generalized linear model (GLM):

1 $$\begin{aligned} Y_i \sim \text {NB}_\theta (\mu _i); \quad \log (\mu _i) = \gamma X_i + \beta ^T Z_i, \end{aligned}$$

where $\text {NB}_{\theta }(\mu _i)$ denotes a negative binomial distribution with mean $\mu _i$ and size parameter $\theta$, and $\gamma \in \mathbb {R}$ and $\beta \in \mathbb {R}^p$ are unknown constants (in fact, SCEPTRE in theory is compatible with arbitrary GLMs, including Poisson GLMs, which may be more appropriate for highly sparse data). SCEPTRE is a permutation test that uses as its test statistic the *z*-score that results from testing the hypothesis $\gamma = 0$ in the model (1). We present the basic SCEPTRE algorithm in Algorithm 1. Several key accelerations speed Algorithm 1 by multiple orders of magnitude.

**Algorithm 1**: Basic SCEPTRE algorithm



#### Acceleration 1: Score test

First, we use a GLM score test to compute the test statistics $z_\text {orig}$, $z_1, \dots , z_B$. Consider the following simplified NB GLM in which the gene expression $Y_i$ is modeled as a function of the technical factor vector $Z_i$ only:

2 $$\begin{aligned} Y_i \sim NB_{\theta }(\mu _i); \quad \log (\mu _i) = \beta ^T Z_i. \end{aligned}$$

Regressing *Y* onto *Z* by fitting the GLM (2) produces estimates $\hat{\beta }$ and $\hat{\theta }$ of the coefficient vector $\beta$ and the size parameter $\theta$, respectively, under the null hypothesis of no relationship between the gRNA indicator and the gene expression. Denote the *i*th fitted mean of the model by $\hat{\mu }_i = \exp (\hat{\beta }^TZ_i),$ and let $\hat{\mu } = [\hat{\mu }_1, \dots , \hat{\mu }_n]^T$ be the vector of fitted means. We can test the gRNA indicator vector *X* for inclusion in the fitted model by computing a score statistic $z_\text {score}$, as follows.

3 $$\begin{aligned} z_\text {score} = \frac{X^T W M (Y - \hat{\mu })}{\sqrt{X^T W X - X^T W Z (Z^T W Z)^{-1} Z^T W X}}. \end{aligned}$$

This expression is derived in the “Derivation of the expression for the GLM score test statistic” section of Additional file 1. Here, *W* and $M(Y - \hat{\mu })$ are a matrix and vector, respectively, that depend on the fitted means $\hat{\mu }$, gene expressions *Y*, and estimated size $\hat{\theta }$:

4 $$\begin{aligned} W = \text {diag}\left\{ \frac{\hat{\mu }_1}{1 + \hat{\mu }_1/\hat{\theta }}, \dots , \frac{\hat{\mu }_n}{1 + \hat{\mu }_n/\hat{\theta }} \right\} ; \quad M(Y - \hat{\mu }) = \left[ \frac{Y_1}{\hat{\mu }_1} - 1, \dots , \frac{Y_n}{\hat{\mu }_n} - 1\right] ^T. \end{aligned}$$

The vector $M(Y - \hat{\mu })$ is a quantity called the working residual. The score statistic (3) is asymptotically equivalent to the Wald or likelihood ratio statistic that one obtains by testing $H_0: \gamma = 0$ in the full model (1). However, unlike the Wald statistic, the score statistic only depends on a fit of the model under the null hypothesis. SCEPTRE (with score statistic; Algorithm 2) exploits this useful property of the score statistic to accelerate the basic SCEPTRE algorithm.

**Algorithm 2:** SCEPTRE (with score statistic) algorithm



#### Acceleration 2: A fast score test for binary treatments

Calculating the score statistic (3) is not trivial. The quadratic form

$$X^TWZ(Z^TWZ)^{-1}Z^TWX$$

in the denominator of (3) is hard to compute, as the matrix $WZ(Z^TWZ)^{-1}Z^TW$ is a large, dense matrix. The classical solution is to algebraically manipulate the score statistic so that it can be evaluated via a QR decomposition. However, the QR decomposition approach does not leverage the structure in *X* when *X* contains many zeros (as is the case in single-cell CRISPR screen analysis). We therefore devised an alternate strategy for computing the score statistic that instead is based on a spectral decomposition; the proposed strategy is tens to hundreds of times faster than the QR decomposition approach when the treatment vector is sparse, as shown in the “Comparing the spectral decomposition algorithm to the QR decomposition algorithm for computing GLM score tests” section of Additional file 1.

First, observe that $Z^T W Z$ is a symmetric matrix. Thus, $Z^T W Z$ can be spectrally decomposed as $Z^T W Z = U^T \Lambda U,$ where *U* is an orthonormal matrix and $\Lambda$ is a diagonal matrix of eigenvalues. Exploiting this decomposition, we can express the quadratic form in the denominator of (3) as follows:

$$X^T W Z (Z^T W Z)^{-1} Z^T W X = X^T W Z U \Lambda ^{-1/2} \Lambda ^{-1/2} U^T Z^T W X = L^T L = ||L||^2,$$

where $L = \Lambda ^{-1/2}U^TZ^TWX$ is a *p*-dimensional vector. Evaluating the above expression reduces to computing the vector *L* and then summing over the squared entries of *L*, which is fast. This observation motivates Algorithm 3, which computes the score statistics for $X, \tilde{X}_1, \dots , \tilde{X}_B$ via a spectral decomposition^3^. The inner product and matrix-vector multiplication operations of step 3 are extremely fast because $X_\text {curr}$ is sparse. Furthermore, we program step 3 in C++ (via Rcpp [45]) for maximum speed.

**Algorithm 3**: Computing the GLM score statistics for $X, \tilde{X}_1, \dots , \tilde{X}_B$  via spectral decomposition.Below, w is the n-dimensional vector constructed from the diagonal entries of W



#### Acceleration 3: Adaptive permutation testing

Computing a large number of permutation resamples for a gene-gRNA pair that yields an unpromising *p*-value after only a few thousand resamples is wasteful. To reduce this inefficiency, we implement a two-step adaptive permutation testing scheme. First, we compute the *p*-value of a given gene-gRNA pair out to a small number (e.g., $B_1 = 500$) of resamples. If this initial *p*-value is unpromising (i.e., if it exceeds some pre-selected threshold of $p_\text {thresh}$, where $p_\text {thresh} \approx 0.01$), then we return this *p*-value to the user. Otherwise, we draw a larger number ($B_2 = 5000$) of fresh resamples and compute the *p*-value using this second set of resamples. As most pairs are expected to be null (and thus yield unpromising *p*-values), this procedure eliminates most of the compute associated with carrying out the permutation tests.

#### Acceleration 4: Skew-normal fit

The null distribution of the test statistics $z_1, \dots , z_B$ converges to a standard Gaussian distribution as the number of cells increases. Thus, to compute a precise *p*-value using a small number of permutations, we fit a skew-normal distribution to the set of null statistics. We then compute a *p*-value by evaluating the tail probability of the fitted skew-normal distribution at the observed test statistic $z_\text {obs}$. If the skew-normal fit to the null statistics is poor (an event that happens rarely), we instead return the standard permutation test *p*-value. We fit the skew-normal distribution via a method of moments estimator and evaluate the skew-normal tail probability via the C++ Boost library. All operations involving the skew-normal distribution are fast.

#### Acceleration 5: Recycling compututation across permutation tests via IWOR sampling

When carrying out a permutation test to test for association between a gene expression vector $Y = [Y_1, \dots , Y_n]^T$ and a perturbation indicator vector $X = [X_1, \dots , X_n]^T$ , SCEPTRE (low-MOI) randomly permutes the perturbation indicator vector *B* times, where *B* is some large number (e.g., $B \approx 5000$). Unfortunately, randomly permuting the perturbation indicator vector *B* times is slow; this cost becomes prohibitive when testing many perturbation-gene pairs. We therefore derived a novel strategy for “sharing” a set of *B* randomly permuted indicator vectors across all perturbation-gene pairs, even pairs with different numbers of cells containing the targeting perturbation. This strategy — which we call “inductive without replacement” (IWOR) sampling — considerably reduces the cost associated with applying SCEPTRE to the data (in fact, this method is generic, compatible with any permutation-based single-cell CRISPR screen association testing method). IWOR sampling is described in the “Inductive without replacement sampling” section of Additional file 1.

### Statistical robustness property of SCEPTRE

SCEPTRE empirically demonstrated a robustness property that we term “confounder adjustment via marginal permutations” or “CAMP.” We observed evidence of CAMP across our simulation studies (Fig. 4; Additional file 1: Figs. S8, and S11) and real data analyses. We describe CAMP in greater detail here. For simplicity, we consider the version of SCEPTRE that does *not* involve fitting a skew-normal distribution to the null test statistics and instead computes the standard permutation test *p*-value by directly comparing the observed test statistic to the null test statistics. If at least one of the following conditions holds, the left-, right-, and two-tailed SCEPTRE *p*-values are valid: (i) the perturbation is unconfounded (i.e., the vector of technical factors $Z_i$ contains all possible confounders, and $Z_i$ is independent of $X_i$); (ii) the NB GLM (1) is correctly specified up to the size parameter $\theta$ and the effective sample size is sufficiently large. We reasoned that CAMP enabled SCEPTRE to address the core single-cell CRISPR screen analysis challenges of sparsity, confounding, and model misspecification both in theory and practice. Our evidence of CAMP is empirical; we intend to derive a mathematical proof of CAMP in a follow-up, more theoretical work.

### CAMP simulation study details

We conducted a simulation study (Additional file 1: Fig. S8) to demonstrate the existence and utility of the CAMP phenomenon. We based the simulation study on a gene (namely, *CXCL10*) and perturbation (namely, “CUL3”) from the Papalexi data. Following the notation introduced in the “SCEPTRE (low-MOI) overview” section, let $Y = [Y_1, \dots , Y_n]^T$ denote the vector of gene expressions of *CXCL10* and $X = [X_1, \dots , X_n]^T$ the vector of perturbation indicators of “CUL3.” Next, let $Z_i \in \mathbb {R}^p$ denote the vector of technical factors of the *i*th cell (for $i \in \{1, \dots , n\}$), and let *Z* denote the $n \times p$ matrix formed by assembling the $Z_i$s into a matrix. We regressed *Y* onto *Z* by fitting the GLM (2), yielding estimates $\hat{\beta }$ for $\beta$ and $\theta ^*$ for $\theta$ under the null hypothesis of no association between the perturbation and gene. An examination of $\hat{\beta }$ revealed that the gene expressions *Y* were moderately associated with the technical factors *Z*. Letting $\hat{\mu }_i = \exp (\hat{\beta }^T Z_i)$ denote the fitted mean of cell *i*, we sampled *B* i.i.d. synthetic expressions $\tilde{Y}_i^1, \dots , \tilde{Y}_i^B$ from an NB model with mean $\hat{\mu }_i$ and size parameter $\theta ^*$. We then constructed *B* synthetic gene expression vectors $\tilde{Y}^j = [\tilde{Y}_1^j, \dots , \tilde{Y}_n^j]^T \in \mathbb {R}^n$ for $j \in \{1, \dots , B\}$. Next, we generated a synthetic perturbation indicator vector $\tilde{X} \in \mathbb {R}^n$ such that $\tilde{X}$ was independent of *Z*. To this end, we marginally sampled synthetic perturbation indicators $\tilde{X}_1, \dots , \tilde{X}_n$ i.i.d. from a Bernoulli model with mean $\hat{\pi }$, where $\hat{\pi } = (1/n)\sum _{i=1}^n X_i$ was the fraction of cells that received the targeting perturbation (the observed perturbation indicator vector *X* was moderately associated with *Z*).

We assessed three methods in the simulation study: NB regression, SCEPTRE, and the standard permutation test. We deployed NB regression and SCEPTRE in a slightly different way than usual: we set the NB size parameter $\theta$ upon which these methods rely to a fixed value (typically, NB regression and SCEPTRE estimate $\theta$ using the data). This enabled us to assess the impact of misspecification of the size parameter on the calibration of NB regression and SCEPTRE. We set the test statistic of the standard permutation test to the sum of the gene expressions in the treatment cells. We then generated *B* confounded (resp., unconfounded) datasets by pairing the synthetic response vectors $\tilde{Y}_1, \dots , \tilde{Y}_B$ to the design matrix [*X*, *Z*] (resp., $[\tilde{X}, Z]$). We applied the methods to the datasets twice: once setting the SCEPTRE/NB regression size parameter to the correct value of $\theta ^*$ and once setting this parameter to the incorrect value of $5 \cdot \theta ^*.$ We displayed the results produced by the methods in each of the four settings (i.e., confounded versus unconfounded, correct versus incorrect specification of the size parameter; Additional file 1: Fig. S8) on a QQ plot. We sought to show that SCEPTRE maintains calibration in all settings, while the standard permutation test and NB regression break down under confounding and incorrect specification of the size parameter, respectively.

### Positive control analysis

We grouped together gRNAs that targeted the same genomic location, referring to these grouped gRNAs as “gRNA groups” [5]. We constructed positive control pairs by coupling a given gRNA group to the gene or protein that the gRNA group targeted. We developed a Nextflow pipeline to apply all methods to analyze the positive control pairs of all datasets.

### ChIP-seq enrichment analysis

We obtained ChIP-seq data for CD14+ monocyte cultures with MCSF (10ng/ml) and stimulated with IFN-gamma (100U/ml) for 24 h [30]. Peaks were screened based on their score, with the top 25% selected using the enrichment score as the criterion. We identified downstream target genes for transcription factors by identifying genes with transcription start sites located within 5 kb upstream or downstream of a peak. Using ChIP-seq as the benchmark, our objective was to assess the consistency between the genes associated with a given transcription factor as identified by SCEPTRE and the downstream target genes determined by ChIP-seq. To achieve this, we calculated odds ratios and their corresponding *p*-values using a Fisher exact test on the contingency table comprised of genes found to be affected by knockout of the perturbed gene, as identified by SCEPTRE, and genes whose promoter regions overlapped with a ChIP-seq peak. We conducted this analysis for both STAT1 and IRF1. We also performed this analysis across competing methods, as shown in Additional file 1: Fig. S7.

### Comparison of SCEPTRE (low-MOI) and SCEPTRE (high-MOI)

SCEPTRE (low-MOI) is a substantial statistical and computational extension of SCEPTRE (high-MOI). Below, we outline the ways in which SCEPTRE (low-MOI) differs from SCEPTRE (high-MOI) version 0.0.2, which is the version of SCEPTRE (high-MOI) available on our website at the time of submission.

- SCEPTRE (low-MOI) carries out inference via a permutation test, while SCEPTRE (high-MOI) does so via a conditional randomization test. Given that the low-MOI problem suffers from stronger sparsity, while the high-MOI problem suffers from greater confounding, we reasoned that permutations would yield better calibration in the low-MOI setting.
- SCEPTRE (low-MOI) uses a full GLM score statistic as its test statistic, while SCEPTRE (high-MOI) uses a distilled GLM score statistic. The full score statistic is more powerful than its distilled counterpart, yielding a greater number of discoveries. Moreover, the full score statistic used by SCEPTRE (low-MOI) is supported by a novel and fast algorithm for computing GLM score tests.
- SCEPTRE (low-MOI) leverages a novel algorithm for recycling computation across a large number of permutation tests, thereby considerably decreasing computational cost. This approach — which we term “inductive without replacement sampling” — is described in the “Inductive without replacement sampling” section of Additional file 1.
- SCEPTRE (low-MOI) fits a skew-normal distribution to the null test statistics, while SCEPTRE (high-MOI) fits a skew-*t* distribution to the null test statistics. The skew-normal distribution admits a fast and numerically stable method-of-moments estimator, while the skew-*t* distribution requires a slow and (relatively) numerically unstable maximum likelihood estimator.
- SCEPTRE (low-MOI) checks for goodness of fit of the fitted skew-normal distribution before it uses the fitted distribution to compute a *p*-value. SCEPTRE (high-MOI), by contrast, does not check for goodness of fit of the fitted skew-*t* distribution, which potentially can lead to miscalibrated *p*-values for perturbation-gene pairs whose resampling distributions are not approximately skew-*t*-distributed.
- SCEPTRE (low-MOI) uses an adaptive permutation testing scheme to reduce the number of permutations computed for pairs with unpromising *p*-values. SCEPTRE (high-MOI), by contrast, does not leverage any sort of adaptive resampling scheme.
- SCEPTRE (high-MOI) is programmed entirely in R. SCEPTRE (low-MOI) by contrast is programmed in a mix of C++ and R, with the computationally intensive portions programmed in C++.
- SCEPTRE (low-MOI) uses a considerably faster method than SCEPTRE (high-MOI) for fitting the negative binomial regression models.
- SCEPTRE (low-MOI) includes support for “pairwise quality control,” in which low-quality perturbation-gene pairs (defined as pairs whose effective sample size falls below some threshold) are detected and removed.
- SCEPTRE (low-MOI) can automatically construct negative control pairs that are “matched” to the discovery pairs in several respects; these negative control pairs can be used to assess the calibration of SCEPTRE (low-MOI) on a user-inputted dataset. SCEPTRE (high-MOI) does not have such functionality.
- SCEPTRE (low-MOI) by default uses the set of negative control cells as its control group; this choice is especially appropriate for gene-targeting screens. SCEPTRE (high-MOI), by contrast, uses the complement set as its control group, as this is the only option, since few (if any) cells contain exclusively non-targeting perturbations in the high-MOI setting.
- SCEPTRE (low-MOI) includes new functions for visualizing the results, including a function to create a volcano plot and a function to create a QQ plot with the discovery *p*-values superimposed on top of the negative control *p*-values.

Taken together, these extensions make SCEPTRE (low-MOI) faster, more memory efficient, more statistically powerful, more statistically robust, more numerically stable, and more user-friendly than SCEPTRE (high-MOI). We anticipate that many of these extensions can be applied to improve the high-MOI functionality of SCEPTRE as well (important differences between the two modules, however, including the choice of control group, will remain). We intend to explore this possibility in future work.

### Methods not included in the benchmarking analysis

Several methods that recently have been proposed for single-cell CRISPR screen analysis were not included in our benchmarking study. First, guided sparse factor analysis (GSFA; introduced by Zhou et al. [39]) couples factor analysis to differential expression analysis to infer the effects of perturbations on gene modules and individual genes. GSFA is a Bayesian method, returning a posterior inclusion probability instead of a *p*-value for each test of association. Given that the methods that we studied in this work were frequentist (and thus returned a *p*-value), we deprioritized GSFA for benchmarking. Next, Normalisr (proposed by Wang [17]) is a method for single-cell differential expression, co-expression, and CRISPR screen analysis. Normalisr non-linearly transforms the gene expression counts to Gaussianity and then models the transformed counts via a linear model. We were unable to locate an example low-MOI single-cell CRISPR screen analysis in the Normalisr Github repository (although gene co-expression, case-control differential expression, and high-MOI CRISPR screen examples are available). Given this, and given the complexity of the Normalisr codebase, we deprioritized Normalisr for benchmarking. Finally, scMaGECK (proposed by Yang [14]) tests for association between CRISPR perturbations and gene expressions using a permutation test with a linear regression coefficient test statistic. Given that we carefully evaluated the high-MOI version of scMaGECK in our prior work [13], and given that MIMOSCA — benchmarked in this work — also is based on a permutation test with a linear regression coefficient test statistic, we deprioritized scMaGECK for benchmarking.

### Supplementary information

**Additional file 1.** Contains the supplementary tables and figures referenced in the main text, additional mathematical details of SCEPTRE (low-MOI), and several additional empirical analyses.

**Additional file 2.** Review history.

## Data Availability

The code for this paper is contained across nine Github repositories. Navigate to github.com/Katsevich-Lab/sceptre2-manuscript (i.e., the second Github repository of those listed) for instructions on reproducing the analyses reported in this manuscript. 1. The sceptre package implements the SCEPTRE method. It is released under a GPL-3.0 license. The repository contains detailed tutorials and examples. katsevich-lab.github.io/sceptre 2. The sceptre2-manuscript repository [46, 47] contains code to reproduce all analyses reported in this paper. It is the main reproduction repository associated with this manuscript. It is released under a GPL-3.0 license. It is deposited at Zenodo with DOI 10.5281/zenodo.10976334. github.com/Katsevich-Lab/sceptre2-manuscript 3. The lowmoi package implements the existing single-cell CRISPR screen analysis methods (methods originally written in Python are implemented via reticulate). github.com/Katsevich-Lab/lowmoi 4. The undercover-grna-pipeline repository contains the Nextflow pipeline to carry out negative control benchmarking analysis. github.com/Katsevich-Lab/undercover-grna-pipeline 5. The pc-grna-pipeline repository contains the Nextflow pipeline to carry out the positive control benchmarking analysis. github.com/Katsevich-Lab/pc-grna-pipeline 6. The ondisc package implements data structures that we use to store the single-cell expression data. github.com/timothy-barry/ondisc 7. The import-frangieh-2021 repository imports and processes the Frangieh data. github.com/Katsevich-Lab/import-frangieh-2021 8. The import-papalexi-2021 repository imports and processes the Papalexi data. github.com/Katsevich-Lab/import-papalexi-2021 9. The import-schraivogel-2020 repository imports and processes the Schraivogel data. github.com/Katsevich-Lab/import-schraivogel-2020 Next, the results are available on DropBox (https://www.dropbox.com/scl/fo/5uw77telw7uvgatkj0xod/AKAPdd5jdrswtRXahVxY0Tk?rlkey=2mw1kzu4erztqzr9qd6f2ojxq&dl=0). Finally, the processed single-cell CRISPR screen data (stored in ondisc v1.1.0 format) are available on Zenodo (DOI: 10.5281/zenodo.10976417) and on Dropbox (www.dropbox.com/sh/jekmk1v4mr4kj3b/AAAhznGqk-TIZKhW40xiU6ORa?dl=0).

## Footnotes

1 One potentially could make more discoveries using MIMOSCA by increasing the tuning parameter *B*, i.e., the number of times that the dataset is permuted and the test statistic recomputed. However, doing so would considerably increase computational cost.

2 The wilcox.test function on which Seurat-Wilcox relies returns $p_\text {exact}$ only if (i) there are fewer than 50 cells across both treatment and control groups and (ii) no two cells (in either the treatment or the control group) have the same normalized expression level. This condition is expected to hold rarely, if ever.

3 A Cholesky decomposition of $Z^TWZ$ could be used in place of the spectral decomposition, but the spectral decomposition is slightly more general, as it applies to matrices with eigenvalues equal to zero, which can occur (for example) when columns of *Z* are highly correlated.
