# Supplementary material for: Robust differential expression testing for single-cell CRISPR screens at low multiplicity of infection
Source: Genome Biol. 2024 May 17;25:124. doi: 10.1186/s13059-024-03254-2 (PMC11100084; doi:10.1186/s13059-024-03254-2)
Supplement: Supplementary file 1 — Additional file 1. Contains the supplementary tables and figures referenced in the main text, additional mathematical details of SCEPTRE (low-MOI), and several additional empirical analyses. [file 13059_2024_3254_MOESM1_ESM.pdf]

Additional file 1 for *Robust differential expression testing for single-cell CRISPR screens at low multiplicity of infection*

Timothy Barry[1†], Kaishu Mason[2], Kathryn Roeder[3,4], and Eugene Katsevich[2,*]

April 13, 2024

1. Department of Biostatistics, Harvard T.H. Chan School of Public Health
2. Department of Statistics and Data Science, Wharton School, University of Pennsylvania
3. Department of Statistics and Data Science, Carnegie Mellon University
4. Computational Biology Department, Carnegie Mellon University

† First author email: `tbarry@hsph.harvard.edu`

* Last author email: `ekatsevi@wharton.upenn.edu`

Organization of Additional file 1

This document contains supplementary materials for the paper *Robust differential expression testing for single-cell CRISPR screens at low multiplicity of infection.* First, we present the supplementary tables (i.e., Tables S1 — S3) and figures (i.e., S1 — S9) referenced in the main text. Next, we provide additional mathematical details about the SCEPTRE (low-MOI) method, including a discussion of inductive without replacement (IWOR) sampling. Finally, we present several additional empirical analyses, including a comparison of the score statistic and the difference-in-residual-means statistic in the context of permutation testing, a comparison of the spectral decomposition algorithm and the QR decomposition algorithm for computing GLM score tests, and an analysis of the number of cells required per perturbation to ensure adequate power.

S1 Supplementary tables and figures referenced in the main text

Paper	Datasets	CRISPR modality	Platform	Target	Modality measured	Cell type
Frangieh 2021	co-culture, control, IFN-γ (3)	CRISPRko	Perturb-CITE seq	Gene TSSs	Gene expressions*	TIL
Papalexi 2021	ECCITE screen (1)	CRISPRko	ECCITE-seq	Gene TSSs	Gene and protein expressions	THP1
Schraivogel 2020	Enhancer screen (1)	CRISPRi	Targeted perturb-seq	Enhancers	Gene expressions	K562
-	Simulated dataset (1)	-	-	Gene TSSs	Gene expressions	-

Table S1: **Datasets analyzed in this work**. The first column indicates the name of a low-MOI single-cell CRISPR screen paper; the second column indicates the datasets that we obtained from that paper; and the subsequent columns indicate the (paper-specific) biological attributes of the data, including CRISPR modality, technology platform, target type, cellular modality measured, and cell type. *The Frangieh data also contain protein measurements, but we focus exclusively on the gene modality in this work.

Dataset	N genes (or proteins)	N cells	N targeting gRNAs	N NT gRNAs	N neg. control pairs	N pos. control pairs
Frangieh Co-culture	14,438	46,427	744	74	596,344	181
Frangieh control	15,449	30,486	744	74	528,239	170
Frangieh IFN-γ	14,654	50,053	744	74	565,502	181
Papalexi (gene)	14,559	20,729	101	9	100,458	25
Papalexi (protein)	4	20,729	101	9	36	2
Schraivogel*	82 (Chr11), 71 (Chr8)	99,884 (Chr11), 88,715 (Chr8)	3,073 (Chr11), 4,089 (Chr8)	30 (Chr11), 30 (Chr8)	4,693 (pooled)	25 (pooled)
Simulated	4439	10,000	-	25	108,510	-

Table S2: **Statistical attributes of the datasets**. The number of genes, cells, targeting gRNAs, NT gRNAs, negative control pairs, and positive control pairs for each dataset. Neg., negative; pos., positive.

*Schraivogel separately assayed two chromosomes: Chr11 and Chr8. Given the similarity of these assays, we pool together the negative and positive control pairs across assays.

Method	Sparsity	Confounding (beyond library size)	Model misspecification
Seurat-Wilcox	No	No	Yes
Seurat-NB	No	No	No
t-test	Yes	No	No
MAST	No	No	No
KS test	No	No	Yes
NB regression (with covariates)	No	Yes	No
Standard permutation test	Yes	No	Yes
**SCEPTRE**	**Yes**	**Yes**	**Yes**

Table S3: **Analysis challenges addressed by each method**. Each cell indicates whether the method in the row addresses the analysis challenge in the column. SCEPTRE (bottom row) is the only method that addresses all three analysis challenges. Note: MIMOSCA is excluded from this table, as we could not determine which analysis challenge(s) MIMOSCA addresses.

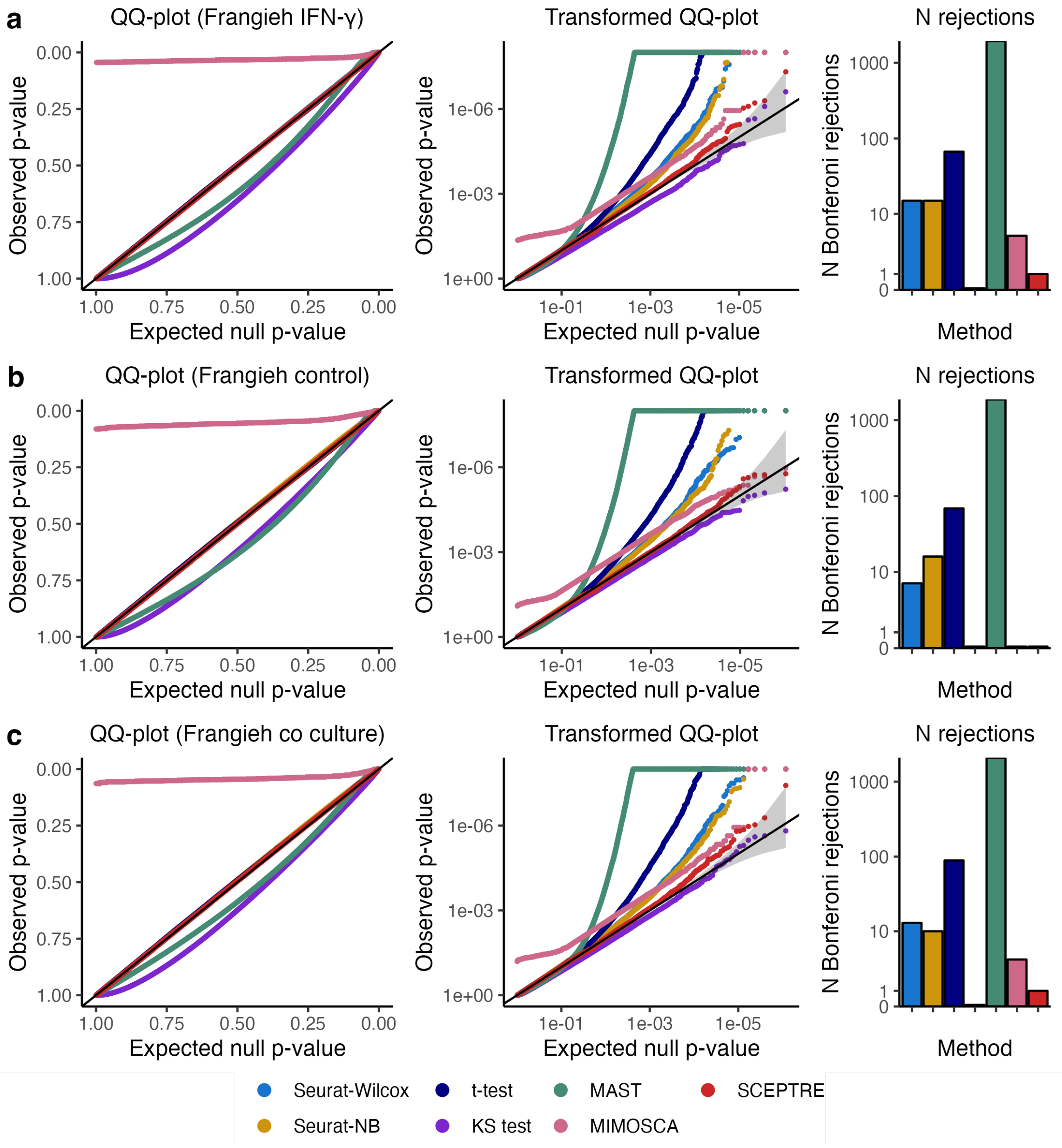

Figure S1: **Calibration results for all methods on Frangieh IFN-γ, Frangieh control, and Frangieh co-culture negative control data.** Left, untransformed QQ plots; middle, negative log-10 transformed QQ plots; right; number of false rejections after a Bonferroni correction at level 0.1.

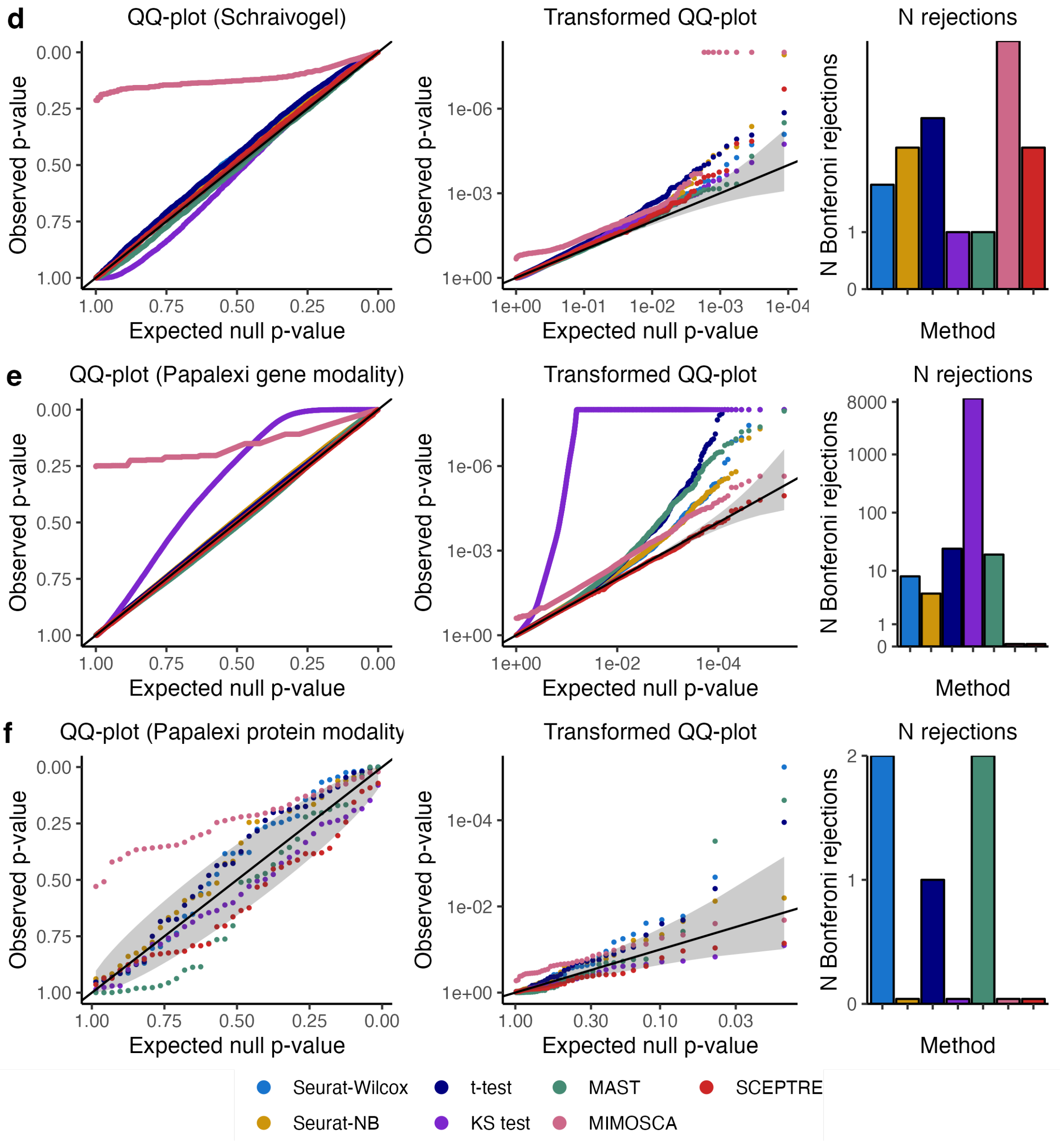

Figure S2: **Calibration results for all methods on Schraivogel, Papalexi (gene modality), and Papalexi (protein modality) negative control data.** Interpretation is the same as in Figure S1.

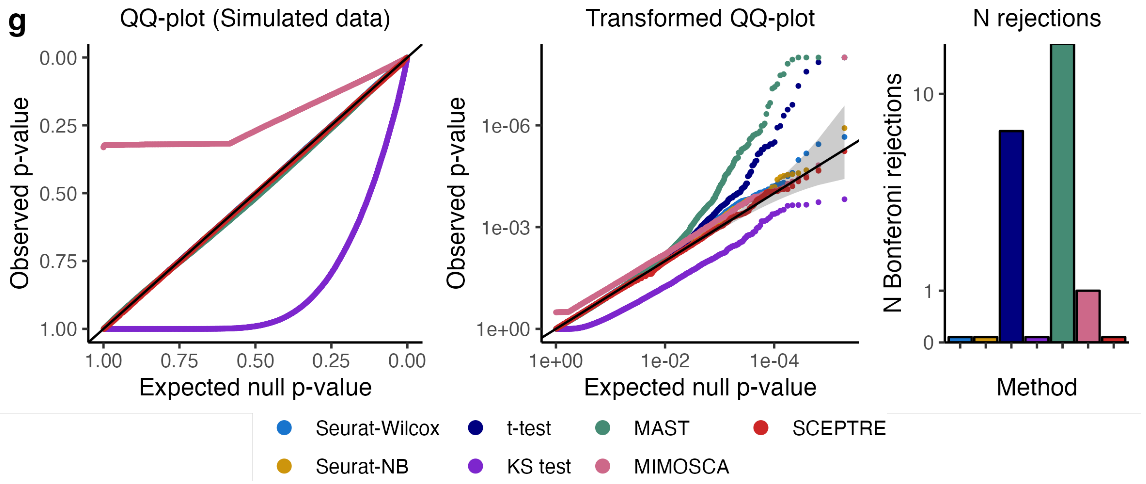

Figure S3: **Calibration results for all methods on simulated data.** Interpretation is the same as in Figure S1.

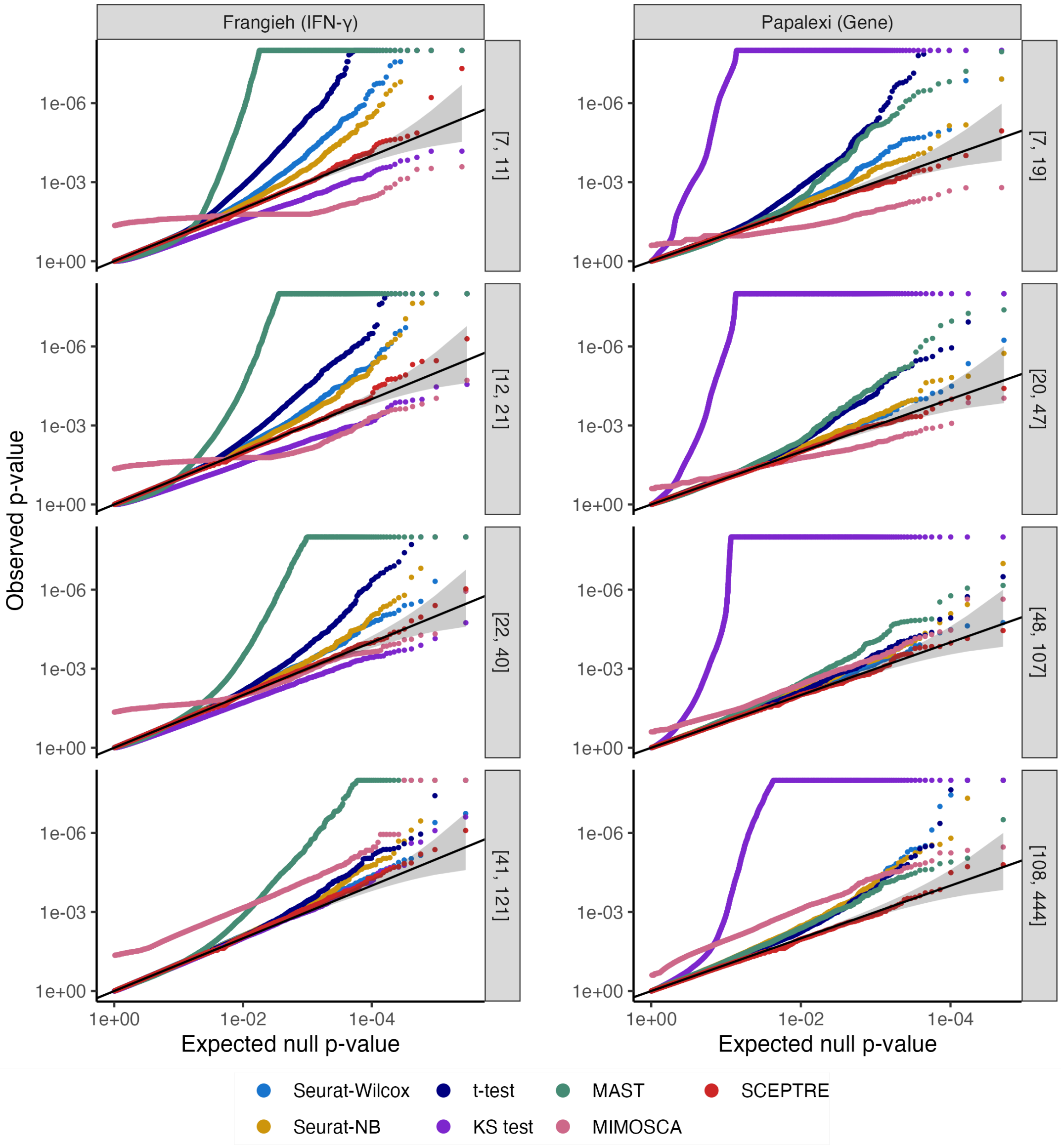

Figure S4: **Calibration results for all methods on Frangieh (IFN-γ) and Papalexi (gene modality) datasets, stratified by effective sample size**. Negative control gene-gRNA pairs are partitioned into four bins of approximately equal size based on the number of treatment cells with nonzero expression in a given pair. The interval on the right-hand side of each panel indicates the minimum and maximum number of treatment cells with nonzero gene expression for pairs in that bin. Some methods (e.g., Seurat-Wilcox on the Frangieh IFN-γ data) exhibit better calibration as the number of treatment cells with nonzero expression increases (i.e., as sparsity decreases).

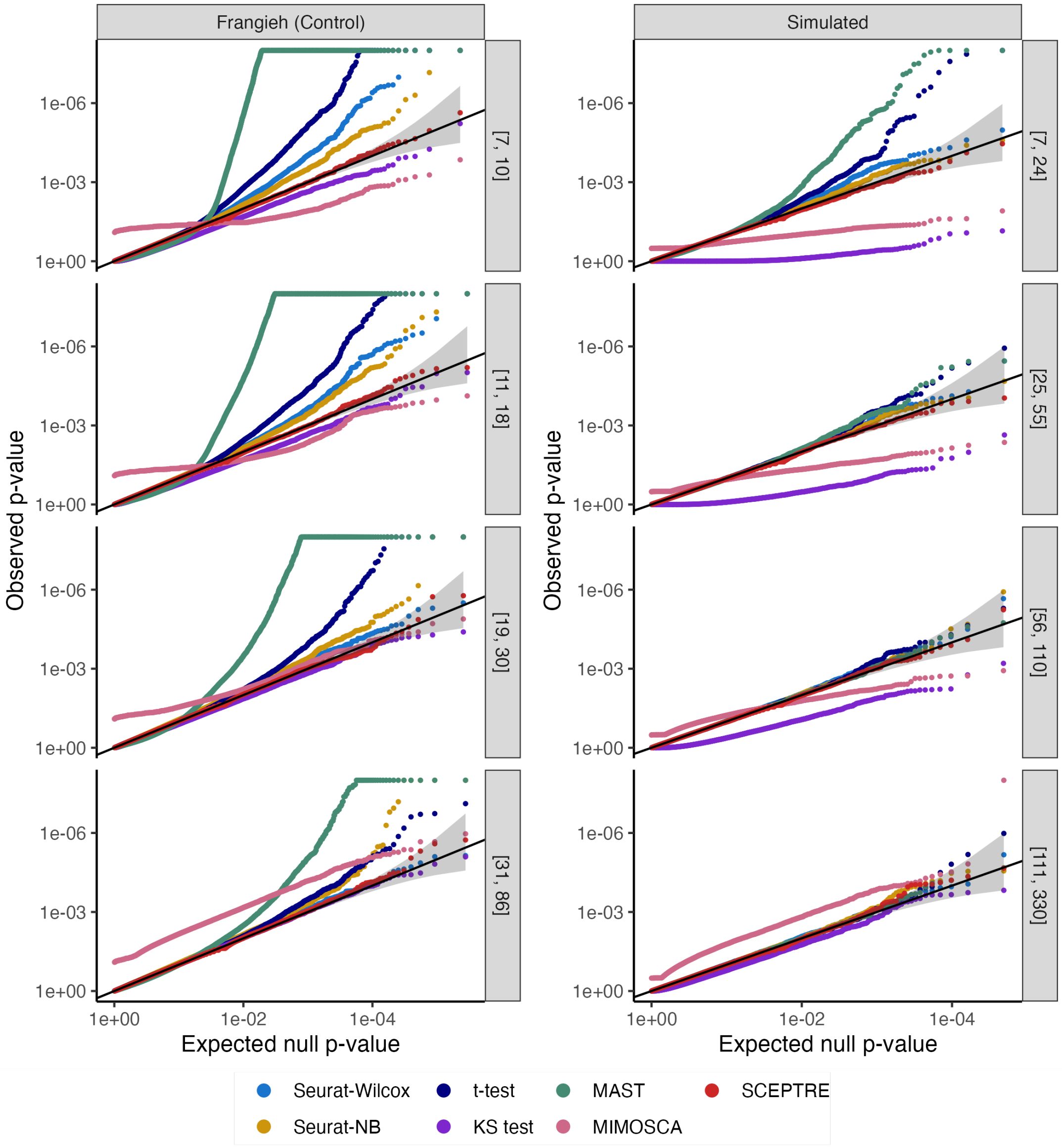

Figure S5: **Calibration results for all methods on Frangieh (control) and simulated datasets, stratified by effective sample size**. This figure is identical to Figure S4 but displays results on the Frangieh (control) and simulated datasets.

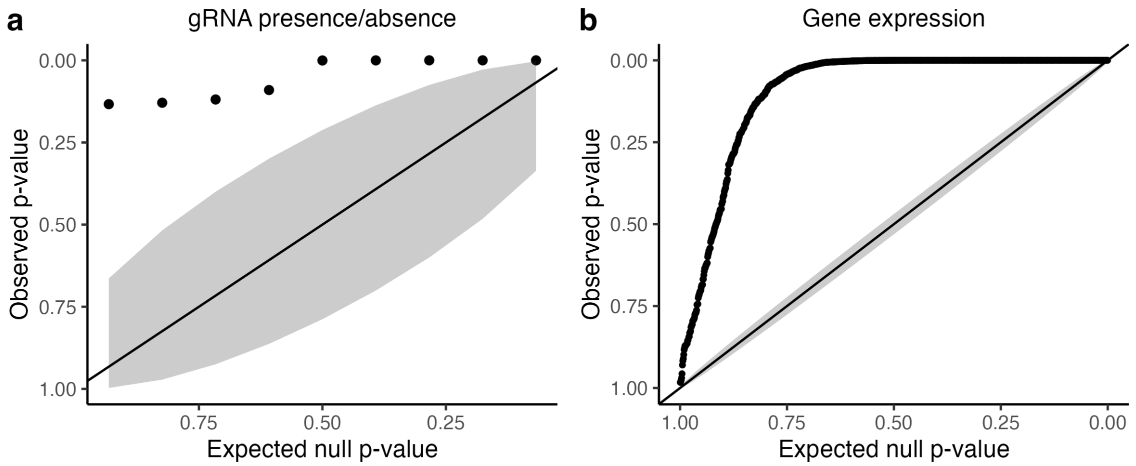

Figure S6: **Confounding due to biological replicate on the Papalexi (gene modality) data.** We conducted a test of association between each NT gRNA and biological replicate. We plotted the resulting p-values on a QQ plot (left); each point in the plot corresponds to a different NT gRNA. Likewise, we carried out a test of association between the expression of each gene and biological replicate (right); each point in the plot similarly corresponds to a different gene. We observed that the two sets of p-values were inflated, indicating that the bulk of the NT gRNAs and the bulk of the genes was associated with biological replicate. Because biological replicate affected *both* gRNA presence/absence *and* gene expression, we reasoned that biological replicate acted as a confounder on the Papalexi data. The section Details of the investigation into the core analysis challenges ("Confounding" subsection) describes how the tests of association were conducted.

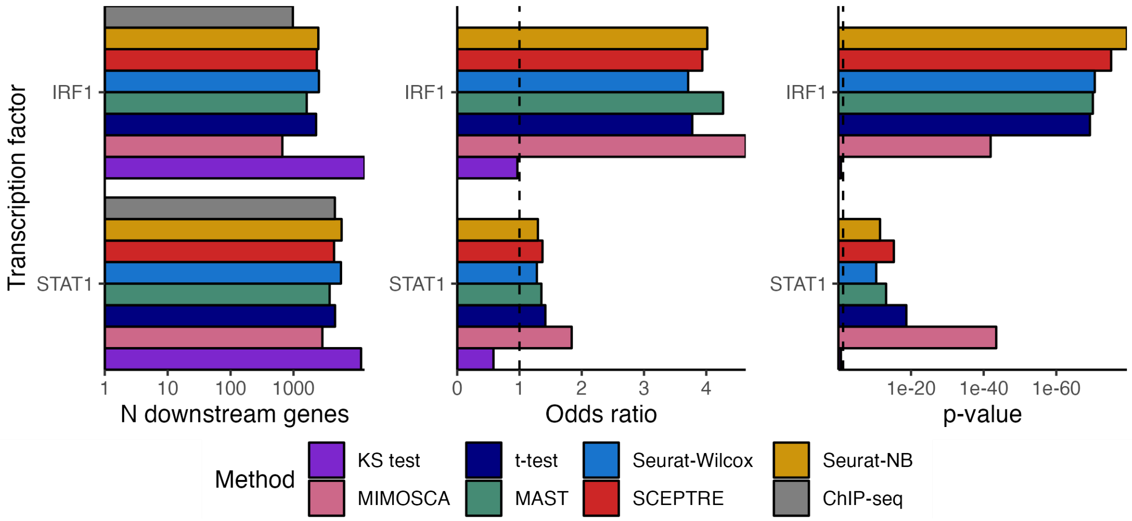

Figure S7: **Comparison of methods for enrichment of ChIP-seq signal on the Papalexi data**. Number of discoveries (after a BH correction at level 0.1), ChIP-seq enrichment odds ratio, and ChIP-seq enrichment p-value of each method for the transcription factors IRF1 and STAT1. The p-value quantifies the extent to which the discovery set of a given method exhibited enrichment for cell-type-relevant ChIP-seq signal. See section ChIP-seq enrichment analysis for a description of how the ChIP-seq data were used to identify the target genes of STAT1 and IRF1.

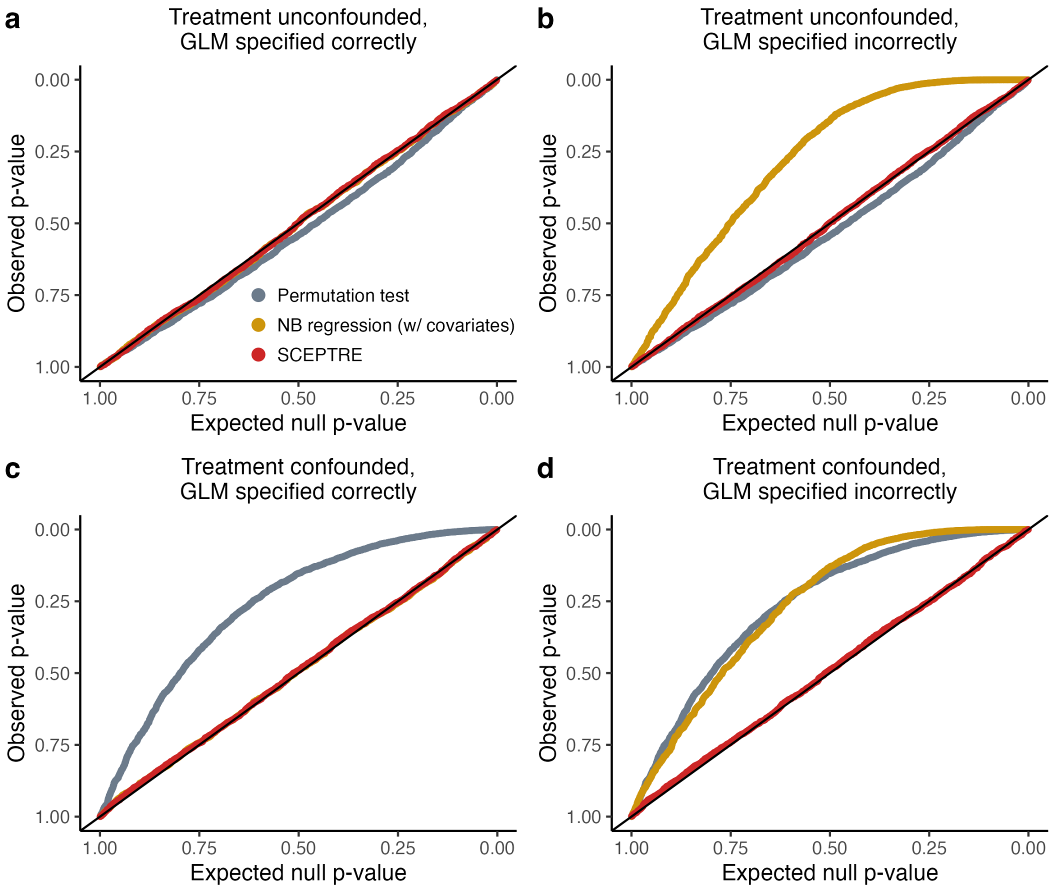

Figure S8: **Demonstration of the CAMP ("confounder adjustment via marginal permutations") phenomenon on realistic semi-synthetic data**. Application of a standard permutation test, NB regression, and SCEPTRE to realistic semi-synthetic data generated under two conditions: confounded and unconfounded. Panels **a** and **b** (resp., **c** and **d**) show the results on the unconfounded (resp., confounded) data; meanwhile, panels **a** and **c** (resp., **b** and **d**) show the results under correct (resp. incorrect) specification of the negative binomial size parameter. The permutation test (gray) works well when the data are unconfounded (panels **a** and **b**) but breaks down in the presence of confounding (panels **c** and **d**). On the other hand, NB regression is well-calibrated when the size parameter is correctly specified (panels **a** and **c**) but fails when the size parameter is misspecified (panels **b** and **d**). SCEPTRE is well-calibrated in all settings. We note that SCEPTRE is expected to break down when the (i) problem is confounded and the NB regression model is arbitrarily misspecified or (ii) the problem is confounded and the sparsity is high. Details of the simulation study are given in Section CAMP simulation study details.

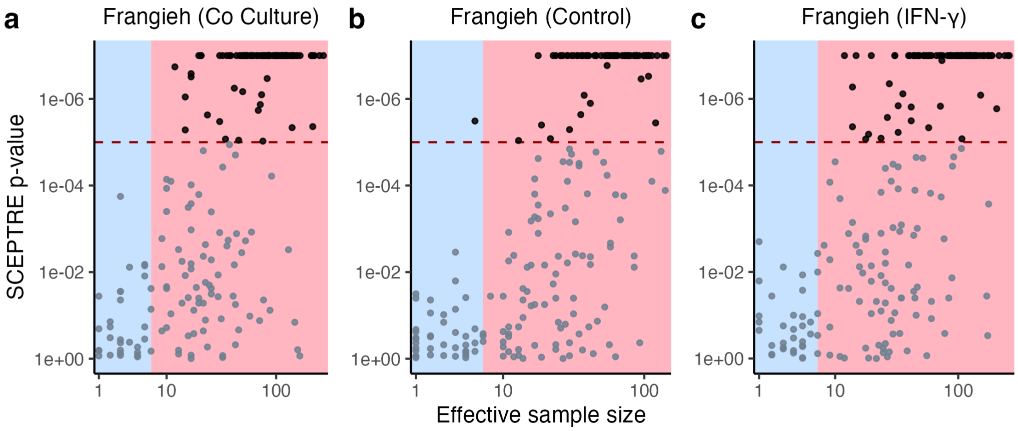

Figure S9: **SCEPTRE's power to detect associations increases as effective sample size increases**. **a-c**, SCEPTRE p-value (truncated at 10^{-6}) versus effective sample size for each pair on the Frangieh co-culture (**a**), control (**b**), and IFN-γ (**c**) positive control data. The horizontal dashed line is drawn at 10^{-5}, demarcating a highly significant discovery. SCEPTRE makes only one rejection at a highly significant level on pairs for which the effective sample size less than seven (blue region).

S2 Additional mathematical details of SCEPTRE (low-MOI)

This section contains additional mathematical details of SCEPTRE (low-MOI). First, we derive the expression for the GLM score statistic used within the method, and next, we describe IWOR sampling.

Derivation of the expression for the GLM score test statistic

We derive the GLM score test statistic for adding a single variable to a fitted GLM model. (We are not aware of any book or paper that presents this derivation.) Let $Y \in \mathbb{R}^n$ be the vector of responses. Let $\boldsymbol{Z} \in \mathbb{R}^{n \times p}$ be the matrix of variables onto which we are regressing Y. (Assume that $\boldsymbol{Z}$ includes a column of ones corresponding to the intercept.) Let $X \in \mathbb{R}^n$ be the vector we seek to test for inclusion in the fitted GLM. Let $\boldsymbol{D} = [X \quad \boldsymbol{Z}] \in \mathbb{R}^{n \times (p+1)}$ be the augmented design matrix. We model the mean μ_i of Y_i according to

$$g(\mu_i) = \sum_{i=1}^{p} \beta_i Z_i + \gamma X_i,$$

where $\beta = [\beta_1, \ldots, \beta_p]^T \in \mathbb{R}^p$ and γ are constants, and $g : \mathbb{R} \to \mathbb{R}$ is a link function. Let $\theta = [\beta, \gamma]^T \in \mathbb{R}^{p+1}$ denote the vector of unknown parameters. Also, let $\eta_i = g^{-1}(\mu_i)$ denote the linear component of the model, and let $V(\mu_i)$ denote the variance of the response y_i given mean μ_i. Standard GLM results indicate that the score u and Fisher information I of the model evaluated at θ are

$$\begin{cases} u(\theta) = \nabla \mathcal{L}(\theta) = \boldsymbol{D}^T W M (Y - \mu) \\ I(\theta) = -\mathbb{E}(\nabla^2 \mathcal{L}(\theta)) = \boldsymbol{D}^T W \boldsymbol{D}, \end{cases}$$

where $\mathcal{L}(\theta)$ is the log-likelihood of the model evaluated at θ,

$$W = \text{diag}\left\{ \frac{1}{V(\mu_i)(d\eta_i/d\mu_i)^2} \right\}_{i=1}^{n}$$

is the matrix of "weights" and

$$M = \text{diag}\left\{ \frac{d\eta_i}{d\mu_i} \right\}_{i=1}^{n}$$

is the matrix of derivatives of the linear component with respect to the mean. We can rewrite the score as

$$u(\theta) = \begin{bmatrix} \boldsymbol{Z}^T \\ X^T \end{bmatrix} W M (Y - \mu) = \begin{bmatrix} \boldsymbol{Z}^T W M (Y - \mu) \\ X^T W M (Y - \mu) \end{bmatrix}.$$

Next, we can rewrite the Fisher information as

$$I(\theta) = \begin{bmatrix} \boldsymbol{Z}^T \\ X^T \end{bmatrix} W [\boldsymbol{Z} \quad X] = \begin{bmatrix} \boldsymbol{Z}^T W \boldsymbol{Z} & \boldsymbol{Z}^T W X \\ X^T W \boldsymbol{Z} & X^T W X \end{bmatrix} = \begin{bmatrix} I_{11} & I_{12} \\ I_{21} & I_{22} \end{bmatrix}.$$

Define I_X by

$$I_X = I_{22} - I_{21} I_{11}^{-1} I_{12}.$$

We have that

$$I_X = X^T W X - X^T W \boldsymbol{Z} (\boldsymbol{Z}^T W \boldsymbol{Z})^{-1} \boldsymbol{Z}^T W X.$$

Let $\hat{\beta}$ be the MLE for β. Next, let u_X denote the component of the score vector corresponding to γ, i.e.,

$$u_X(\theta) = X^T W M (Y - \mu).$$

The value of u_X evaluated at $(\hat{\beta}, 0)$ is

$$u_X(\hat{\beta}, 0) = X^T \hat{W} \hat{M} (Y - \hat{\mu}),$$

where $\hat{W}$, $\hat{M}$, and $\hat{\mu}$ denote W, M, and μ, respectively, evaluated at $(\hat{\beta}, 0)$. Similarly, the value of $I_X(\theta)$ evaluated at $(\hat{\beta}, 0)$ is

$$I(\hat{\beta}, 0) = X^T \hat{W} X - X^T \hat{W} \boldsymbol{Z} (\boldsymbol{Z}^T \hat{W} \boldsymbol{Z})^{-1} \boldsymbol{Z}^T \hat{W} X.$$

Standard asymptotic results indicate that

$$X^T \hat{W} \hat{M} (Y - \hat{\mu}) \xrightarrow{d} N(0, X^T \hat{W} X - X^T \hat{W} \boldsymbol{Z} (\boldsymbol{Z}^T \hat{W} \boldsymbol{Z})^{-1} \boldsymbol{Z}^T \hat{W} X).$$

The GLM score test z-score is therefore

$$z_{\text{score}} = \frac{X^T \hat{W} \hat{M} (Y - \hat{\mu})}{\sqrt{X^T \hat{W} X - X^T \hat{W} \boldsymbol{Z} (\boldsymbol{Z}^T \hat{W} \boldsymbol{Z})^{-1} \boldsymbol{Z}^T \hat{W} X}}. \quad (1)$$

The GLM score test statistic typically is expressed in a different way in books and papers.[1] Let E_2 denote the matrix of residuals after least squares regression of the columns of X onto $\boldsymbol{Z}$, i.e.,

$$E_2 = X - \boldsymbol{Z} (\boldsymbol{Z}^T \hat{W} \boldsymbol{Z})^{-1} \boldsymbol{Z}^T \hat{W} X.$$

Furthermore, define $\hat{r} = \hat{M}(y - \hat{\mu})$ as the "working residual" vector. The GLM score test statistic typically is expressed as

$$z_{\text{score}} = \frac{X^T \hat{W} \hat{r}}{\sqrt{E_2^T \hat{W} E_2}}. \quad (2)$$

The expressions (1) and (2) are identical:

$$\begin{aligned}
&X^T \hat{W} X - X^T \hat{W} \boldsymbol{Z} (\boldsymbol{Z}^T \hat{W} \boldsymbol{Z})^{-1} \boldsymbol{Z}^T \hat{W} X \\
&\quad = X^T \hat{W} X - X^T \hat{W} \boldsymbol{Z} (\boldsymbol{Z}^T \hat{W} \boldsymbol{Z})^{-1} \boldsymbol{Z}^T \hat{W} X \\
&\quad\quad - X^T \hat{W} \boldsymbol{Z} (\boldsymbol{Z}^T \hat{W} \boldsymbol{Z})^{-1} \boldsymbol{Z}^T \hat{W} X \\
&\quad\quad + X^T \boldsymbol{W} \boldsymbol{Z} (\boldsymbol{Z}^T W \boldsymbol{Z})^{-1} \boldsymbol{Z}^T \hat{W} \boldsymbol{Z} (\boldsymbol{Z}^T \hat{W} \boldsymbol{Z})^{-1} \boldsymbol{Z}^T \hat{W} X \\
&\quad = (X - \boldsymbol{Z} (\boldsymbol{Z}^T \hat{W} \boldsymbol{Z})^{-1} \boldsymbol{Z} \hat{W} X)^T \hat{W} (X - \boldsymbol{Z} (\boldsymbol{Z}^T \hat{W} \boldsymbol{Z})^{-1} \boldsymbol{Z} \hat{W} X) \\
&\quad = E_2^T \hat{W} E_2.
\end{aligned}$$

Expression (2) is evaluated via QR decomposition in practice. By contrast, we propose to evaluate (1) via spectral or Cholesky decomposition, thereby exploiting the sparsity of X.

Inductive without replacement sampling

Permutation testing is a computationally expensive statistical technique. There are two main steps involved permutation testing: first, permuting the treatment vector B times, where B is some large integer (e.g., $B = 5,000$), and second, recomputing the test statistic on the observed dataset and the B permuted datasets. Both of these steps are slow; which is slower depends on several factors, including the test statistic in use and the sparsity of the treatment vector. Permutation testing is even more expensive in the "high-throughput setting" in which one seeks to test tens of thousands (or more) of hypotheses via a permutation test. We introduce "inductive without replacement" (IWOR) sampling, a technique for recycling compute across permutation tests in which the number of "control units" is constant across tests (as in low-MOI single-cell CRISPR screens). IWOR sampling is a (provably) approximation-free technique, yielding an exact and correct permutation p-value for every hypothesis tested.

A concrete example of IWOR sampling

We first provide a concrete example of IWOR sampling to help convey intuition for the procedure. (We describe IWOR sampling in a mathematically precise way in the following section.) Suppose that we are analyzing a single-cell CRISPR screen dataset containing 19 cells, four of which contain a non-targeting perturbation, four of which contain targeting perturbation A, five of which contain targeting perturbation B, and six of which contain targeting perturbation C. Suppose that we seek to test for association between each of the targeting perturbations and some gene. Call pair A (resp., pair B, pair C) the pair that results from coupling perturbation A (resp., perturbation B, perturbation C) to the gene.

Figure S10 depicts the "observed treatment vector" corresponding to pair A, pair B, and pair C. For example, the observed treatment vector for pair A contains four treatment cells (blue circles) and four control cells (gray circles), while the observed treatment vector for pair B contains five treatment cells and four control cells, etc. We can enumerate the cells contained within each pair by assigning an integer index to each cell. For example, we can enumerate the cells contained within pair A by $1, \ldots, 8$, and we can enumerate the cells contained within pair B by $1, \ldots, 9$, etc. (Note: although we reuse integer indices across pairs, the cells themselves *are not shared* across pairs.) We seek to permute the observed treatment vector corresponding to each pair B times. This is equivalent to drawing B without replacement samples of size k from the set of indices $1, \ldots, k+N$, where k is the number of treatment cells in a given pair and N is the number of control cells in the dataset. For example, consider the observed treatment vector corresponding to pair A (Figure S10, left column). We can permute this vector by assigning cells 2, 4, 5, and 6 to "treatment" status and assigning the remaining cells to "control" status. Moreover, we can identify this particular permutation of the vector with the set of integers $\{2, 4, 5, 6\}$, where $\{2, 4, 5, 6\}$ can be thought of as a without-replacement sample from the set of indices $1, \ldots, 8$. Here, 8 is the sum of the number of control cells (4) and the number of treatment cells (4).

Our goal is to randomly permute the observed treatment vector corresponding to each pair. IWOR sampling is a strategy for doing this efficiently. An example IWOR sample for this dataset is $v := [5, 6, 4, 2, 9, 10]$ (Figure S10, top). The set that contains the first k elements of v constitutes a valid without-replacement sample of size k from the set of indices $1, \ldots, k + 4$. (The significance of 4 in this context is that 4 is the number of control cells in the example dataset.) For example, the set constructed from the first four elements of v — $\{5, 6, 4, 2\}$ — is a without-replacement sample from the set $1, \ldots, 8$. Thus, $\{5, 6, 4, 2\}$ is a valid permutation of the treatment vector corresponding

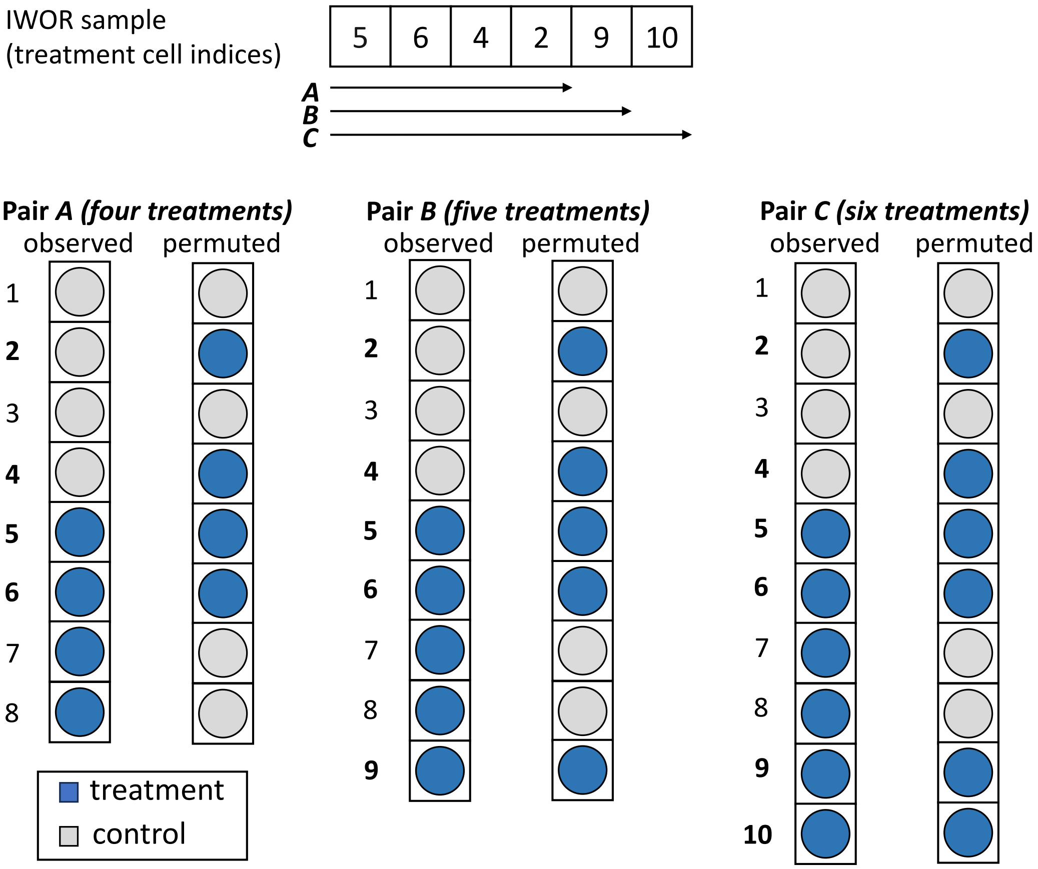

Figure S10: **Inductive without replacement (IWOR) sampling is an approximation-free technique for sharing permutation indices across a large number of permutation tests.** An "IWOR sample" (top) is a single vector from which the set of permutation indices for each hypothesis can be extracted. The permutation indices for pair A consist of the first four elements of the IWOR sample; the permutation indices for pair B consist of the first five elements of the IWOR sample; and the permutation indices for pair C consist of the first six elements of the IWOR sample. Importantly, *the cells themselves are not shared across hypotheses*; rather, the IWOR sample is shared across hypotheses. IWOR sampling assumes that the number of control units is fixed across hypotheses; this assumption holds on low-MOI single-cell CRISPR screen data.

to pair A, which contains four treatment cells.

Next, the set containing the first five elements of v — $\{5, 6, 4, 2, 9\}$ — is a valid without-replacement sample from the set $1, \ldots, 9$. Thus $\{5, 6, 4, 2, 9\}$ is a valid permutation of the treatment vector corresponding to pair B, which contains five treatment cells. Finally, the set constructed from the first six elements of v — v itself — is a valid without replacement sample from the set $1, \ldots, 10$. Thus, v is a valid permutation of the treatment vector corresponding to pair C, which contains six treatment cells. To permute each treatment vector B times, we generate B IWOR samples. For example, four possible IWOR samples for the above dataset are as follows: $[5, 6, 4, 2, 9, 10]$, $[4, 5, 7, 8, 2, 9]$, $[5, 2, 1, 8, 3, 10]$, and $[3, 4, 7, 1, 2, 10]$.

More generally, suppose a given dataset contains N control cells. Let x be a random (i.e., unrealized) IWOR sample, and let $x[1:k]$ denote the first k elements of x. The IWOR sample is carefully constructed such that it satisfies the following crucial property:

$$\mathbb{P}(1 \in x[1:k]) = \mathbb{P}(2 \in x[1:k]) = \cdots = \mathbb{P}(k + N \in x[1:k]) = 1/(k + N).$$

In other words, the first k elements of x constitute a valid WOR sample from the set $\{1, \ldots, k+N\}$. The permutation defined by $x[1:k]$ is therefore a valid permutation for a pair containing k treatment cells and N control cells.

Definition of IWOR sampling

We provide a more precise definition of IWOR sampling here. Consider a gene expression vector $Y \in \mathbb{R}^n$, technical factor matrix $\boldsymbol{Z} \in \mathbb{R}^{n \times p}$, and binary "treatment" vector of perturbation indicators $X \in \{0, 1\}^n$. We can identify a given treatment vector with the set of nonzero entries within that vector. In other words, we can represent the vector X as the set $S = \{i : X[i] = 1\}$, where $X[i]$ denotes the value of the vector X at position i. Suppose that X has m nonzero entries. Randomly permuting X is equivalent to sampling m elements without replacement (WOR) from the set $\{1, \ldots, n\}$. Unfortunately, drawing B WOR samples for every perturbation-gene pair — which is required for carrying out the permutation test dataset-wide — is slow. IWOR sampling shares WOR samples across pairs.

A key observation is that the number of control cells is fixed across all hypotheses, while the number of treatment cells — i.e., the number of cells containing the targeting perturbation — varies from hypothesis to hypothesis (depending on the targeting perturbation). We exploit this structure of the problem as follows. Let N be the number of control cells; label the control cells by $c_1, c_2, \ldots, c_N$. Assume that there are p targeting perturbations. For perturbation $i \in \{1, \ldots, p\}$, let k_i be the number of cells containing perturbation i. Finally, let $M = \max_{i \in \{1,\ldots,p\}} k_i$ be the number of cells containing the perturbation that infects the greatest number of cells. Label the treatment cells by $t_1, \ldots, t_M$. We construct a length-M random sequence $a_1, a_2, \ldots, a_M$ that satisfies the following three properties:

1. $a_i \in \{c_1, \ldots, c_N, t_1, \ldots, t_i\}$ for all $i \in \{1, \ldots, M\}$; that is, the ith element of the sequence is a control cell or one of the first i treatment cells.

2. $a_i \neq a_j$ for $i \neq j$. That is, the elements of the sequence are unique.

Let $A_i := \{a_1, \ldots, a_i\}$ denote the set containing the first i elements of the sequence. (Observe that there are $N + i$ elements that possibly could be in A_i: the N control cells and the first i treatment cells.) We additionally require the following property to hold:

3. $\mathbb{P}(c_1 \in A_i) = \cdots = \mathbb{P}(c_N \in A_i) = \mathbb{P}(t_1 \in A_i) = \cdots = \mathbb{P}(t_i \in A_i) = i/(i+N)$. That is, each of the elements that possibly could be in A_i has an equal chance of being in A_i.

The sequence $a_1, \ldots, a_M$ yields a sequence of increasing sets $A_1 \subset A_2 \subset \cdots \subset A_m$. The first set A_1, which contains a single element, contains the control cells and the first treatment cell with equal probability. The second set A_2, which contains two elements, contains the control cells and each of the first *two* treatment cells with equal probability, and so on. Thus, if a given gene-perturbation pair contains $k \leq M$ treatment cells, then A_k constitutes a valid WOR sample for that pair. Importantly, because the sequence of sets $A_1, \ldots, A_M$ is increasing, we can share the random sample $a_1, \ldots, a_M$ across all gene-perturbation pairs. We call this technique "inductive without replacement sampling" (or "IWOR sampling"). The SCEPTRE (low MOI) algorithm generates a set of B length-M IWOR samples and shares this set of samples across all pairs.

An abstract approach for constructing an IWOR sample

We describe an abstract approach for constructing an IWOR sample and prove its correctness. The procedure is inductive.

Step 1. Sample one element from the set $\{c_1, \ldots, c_N, t_1\}$, putting an equal mass of $1/(N+1)$ onto each of the elements. Call the sampled element a_1. Set $A_1 = \{a_1\}$.
Step i **for** $i \in \{2, \ldots, M\}$. Let $B_i := \{c_1, \ldots, c_N, t_1 \ldots, t_{i-1}\} \setminus A_{i-1}$ denote the set of "leftover" elements not sampled in steps $1, \ldots, i-1$. There are N elements in the set B_i. Draw an element at random from the set $B_i \bigcup \{t_i\}$, placing a mass of $\frac{i}{i+N}$ on t_i and a mass of $\left(1 - \frac{i}{i+N}\right)/N$ on each of the elements in B_i. Call the sampled element a_i. Set $A_i = A_{i-1} \cup \{a_i\}$.

The resulting sequence $a_1, \ldots, a_M$ satisfies the three properties listed above, which we now prove.

Proof: It is clear that $a_i \in \{c_1, \ldots, c_N, t_1, \ldots, t_i\}$ for all i and that $a_i \neq a_j$ for $i \neq j$. We focus on proving the third property, which we do inductively.

Base case: Let $i = 1$. Then

$$\mathbb{P}(c_1 \in A_1) = \ldots \mathbb{P}(c_N \in A_1) = \mathbb{P}(t_1 \in A_1) = 1/(1+N).$$

Inductive step: Suppose that

$$\mathbb{P}(c_1 \in A_i) = \cdots = \mathbb{P}(c_N \in A_i) = \mathbb{P}(t_1 \in A_i) = \cdots = \mathbb{P}(t_i \in A_i) = \frac{i}{N+i}.$$

To construct the set A_{i+1}, we sample t_{i+1} with probability $(i+1)/(N+i+1)$ and each element of B_i with probability

$$\frac{1}{N}\left(1 - \frac{i+1}{N+i+1}\right).$$

We call the sampled element a_{i+1}, and we set $A_{i+1} = A_i \cup \{a_{i+1}\}$. Our goal is to show that, for all $u \in \{c_1, \ldots, c_N, t_1, \ldots, t_{i+1}\}$,

$$\mathbb{P}(u \in A_{i+1}) = \frac{i+1}{N+i+1}. \tag{3}$$

(That is, for all elements that *could* be in A_{i+1}, each has an equal chance of *actually being* in A_{i+1}.) The equality (3) holds for $u = t_{i+1}$ by construction. Next, suppose that $u \in \{c_1, \ldots, c_N, t_1, \ldots, t_i\}$. We compute $\mathbb{P}(u \in A_{i+1})$ using the law of total probability:

$$\mathbb{P}(u \in A_{i+1}) = \mathbb{P}(u \in A_{i+1} | u \in A_i)\mathbb{P}(u \in A_i) + \mathbb{P}(u \in A_{i+1} | u \notin A_i)\mathbb{P}(u \notin A_i). \tag{4}$$

We consider first the term $\mathbb{P}(u \in A_{i+1}|u \in A_i)\mathbb{P}(u \in A_i)$. We have that $\mathbb{P}(u \in A_{i+1}|u \in A_i) = 1$, as membership of u in A_i implies membership of u in A_{i+1}. By the inductive hypothesis, $\mathbb{P}(u \in A_i) = i/(N+i)$, implying that

$$\mathbb{P}(u \in A_{i+1}|u \in A_i)\mathbb{P}(u \in A_i) = i/(N+i). \tag{5}$$

Next, we consider the term $\mathbb{P}(u \in A_{i+1}|u \notin A_i)\mathbb{P}(u \notin A_i)$. If $u \notin A_i$ (i.e., if u is a "leftover" element at step i), then $u \in B_i$, implying

$$\mathbb{P}(u \in A_{i+1}|u \notin A_i) = \frac{1}{N}\left(1 - \frac{i+1}{N+1+i}\right)$$

by construction. Furthermore, by the inductive hypothesis, $\mathbb{P}(i \notin A_i) = 1 - \mathbb{P}(u \in A_i) = 1 - i/(N+i)$. Thus,

$$\mathbb{P}(u \in A_{i+1}|u \notin A_i)\mathbb{P}(u \notin A_i) = \frac{1}{N}\left(1 - \frac{i+1}{N+1+i}\right)(1 - i/(N+i)). \tag{6}$$

Combining (4), (5), and (6),

$$\begin{aligned}
\mathbb{P}(u \in A_{i+1}) &= \frac{i}{N+i} + \frac{1}{N}\left(1 - \frac{i+1}{N+1+i}\right)\left(1 - \frac{i}{N+i}\right) \\
&= \frac{i}{N+i} + \frac{1}{N}\left(\frac{N+1+i}{N+1+i} - \frac{i+1}{N+1+i}\right)\left(\frac{N+i}{N+i} - \frac{i}{N+i}\right) \\
&= \frac{i}{N+i} + \frac{1}{N}\left(\frac{N}{N+1+i}\right)\left(\frac{N}{N+i}\right) = \frac{i}{N+i} + \frac{N}{(N+1+i)(N+i)} \\
&= \frac{i(N+1+i)+N}{(N+i)(N+1+i)} = \frac{iN+i+i^2+N}{(N+1)(N+1+i)} = \frac{i+1}{N+1+i}.
\end{aligned}$$

□

A concrete algorithm for IWOR sampling

We now derive a concrete algorithm for constructing an IWOR sample of size M on the set $\{c_1, \ldots, c_N, t_1, \ldots, t_M\}$. The algorithm requires only a high-quality uniform random number generator, which is readily available in most programming languages. First, we describe a simple algorithm for sampling from the discrete probability distribution with support $\{0, \ldots, N\}$ that places mass $i/(i+N)$ on N and mass $(1/N)[1 - i/(i+N)]$ on $\{0, 1, ..., N-1\}$. (We call this distribution the $\texttt{IWOR}(N, i)$ distribution.) The algorithm, given in Algorithm 1, takes $\mathcal{O}(1)$ time and space.

Next, we provide an algorithm for constructing an IWOR sample of length M on the set of control cells $\{c_1, \ldots, c_N\}$ and treatment cells $\{t_1, \ldots, t_M\}$. Algorithm 2 is a concrete instantiation of the "abstract approach for constructing an IWOR sample," as described in the previous section. The vector r is a vector of length $N+1$. At step i, the N cells that remain (i.e., those that have not yet been sampled among $\{c_1, ..., c_N, t_1, \ldots, t_{i-1}\}$) are stored in the leftmost N positions of r. The cell t_i is then inserted into the rightmost position of r. A cell is sampled from r via the $\texttt{IWOR}(N, i)$ distribution, which places more mass on the cell in the rightmost position (i.e., t_i). The sampled cell is moved into v. Then, the cell in the rightmost entry of r (i.e., t_i) is moved into the position vacated by the sampled cell. Finally, the cell t_{i+1} is moved into the rightmost position of r. We

Algorithm 1: Generating a sample from the `IWOR`(N, i) distribution.

Input N and i
$u \sim U(0,1)$
$p \leftarrow i/(N+i)$
if $u > 1 - p$ **then**
  $d \leftarrow N$
else
  $d \leftarrow \lfloor uN/(1-p) \rfloor$ // floor operator
end
return d

repeat this process until we have iterated through all of the treatment cells. This algorithm is fast and efficient, taking $\mathcal{O}(M)$ time and $\mathcal{O}(M+N)$ space.

Algorithm 2: Generating an IWOR sample of size M given control cells $c_1, \ldots, c_N$ and treatment cells $t_1, \ldots, t_M$.

Input Control cells $c_1, \ldots, c_N$ and treatment cells $t_1, \ldots, t_M$.
Initialize an empty vector v of size M.
Initialize the vector $r \leftarrow [c_1, c_2, \ldots, c_N, t_1]$.
for $i = 1 \ldots M$ **do**
  `pos` $\sim$ IWOR(N, i) // sample a position within r
  $v[i-1] \leftarrow r[$`pos`$]$ // move the element at that position into v
  $r[$`pos`$] \leftarrow r[N]$ // move the rightmost entry of r to position `pos`
  $r[N] \leftarrow t_{i+1}$ // update the rightmost entry of r with t_{i+1}
end
return v

SCEPTRE (low-MOI) uses a slightly modified, more efficient, and more numerically stable version of Algorithm 2 for constructing the IWOR sample. Suppose there are p targeting perturbations, with the ith perturbation infecting k_i cells. Let $M = \max_{i \in \{1,\ldots,p\}} k_i$ be the number of cells containing the perturbation that infects the greatest number of cells, and let $m = \min_{i \in \{1,\ldots,p\}} k_i$ be the number of cells containing the perturbation that infects the least number of cells. SCEPTRE first samples k cells without replacement from the set $c_1, \ldots, c_N, t_1, \ldots, t_k$. Then, SCEPTRE constructs an IWOR sample, taking as input the cells that remain from the first step and treatment cells $t_{k+1}, t_{k+2}, \ldots, t_M$.

S3 Additional empirical analyses

In this section we present several additional empirical analyses.

Comparing the score statistic to the difference-in-residual-means statistic

SCEPTRE (low-MOI) uses a permutation test based on an NB GLM score statistic. A natural question is whether SCEPTRE (low-MOI) is better (in some sense) than the following alternative

method: first, "regress out" the covariates by regressing the response vector onto the covariate matrix via an NB GLM; second, extract the residuals[1] from the fitted GLM; third, perform a permutation test between the treatment vector (i.e., the vector of perturbation presences/absences) and the vector of residuals, taking as the test statistic the standard difference in means statistic. Using mathematical language, let $Y = [Y_1, \ldots, Y_n]^T$ be the vector of gene (or protein, etc.) UMI counts, $X = [X_1, \ldots, X_n]^T$ the vector of perturbation presence or absence indicators, and $Z \in \mathbb{R}^{n \times p}$ the matrix of technical factors (or covariates). Regressing Y onto Z via an NB GLM yields a vector of fitted means $[\hat{\mu}_1, \ldots, \hat{\mu}_n]$. Define the ith (raw) residual by $r_i = y_i - \hat{\mu}_i$. The difference-in-residual-means test statistic T_{resid} is defined as

$$T_{\text{resid}} = \frac{1}{s} \sum_{i:X_i=1} r_i - \left(\frac{1}{n-s} \right) \sum_{i:X_i=0} r_i, \tag{7}$$

where $s = \sum_{i=1}^{n} X_i$ is the number of "treated" cells (i.e., the number of cells containing a perturbation). The GLM score statistic, meanwhile, is defined as

$$T_{\text{score}} = \frac{X^T W M (Y - \hat{\mu})}{\sqrt{X^T W X - X^T W Z (Z^T W Z)^{-1} Z^T W X}}, \tag{8}$$

where W and $M(Y - \hat{\mu})$ are given by

$$W = \text{diag} \left\{ \frac{\hat{\mu}_1}{1 + \hat{\mu}_1/\theta}, \ldots, \frac{\hat{\mu}_n}{1 + \hat{\mu}_n/\theta} \right\}$$

and

$$M(Y - \hat{\mu}) = \left[\frac{Y_1}{\hat{\mu}_1} - 1, \ldots, \frac{Y_n}{\hat{\mu}_n} - 1 \right]^T,$$

respectively. Clearly, T_{score} is more complex than T_{resid}: evaluating the denominator of the former involves performing matrix multiplication, addition, transposition, and inversion operations. How (if at all) do T_{score} and T_{resid} differ with respect to the p-values that they produce? To explore this question, we applied a permutation test based on T_{score} and another based on T_{resid} to real and simulated data, comparing the two methods with respect to type-I error control, power, and computational performance. We found that T_{score} and T_{resid} exhibited similar type-I error control and computational performance but that T_{score} could be much more powerful than T_{resid}. We note that these results — while interesting — are preliminary; we intend to carry out a more in-depth comparison between T_{score} and T_{resid} in a followup, more statistically-focused work.

Simulation study. We first compared T_{resid} to T_{score} on simulated data. We used $n = 5,000$ cells in the simulation study. We constructed the cell-specific covariates $z_1, \ldots, z_n \in \mathbb{R}^3$ by drawing n i.i.d. samples from a bivariate normal distribution and appending a one for the intercept term. (The cell-specific covariates were meant to represent technical factors, such as sequencing depth and percent mitochondrial reads.) Next, we generated the ith binary treatment indicator x_i by sampling x_i from a Bernoulli distribution with mean $\mu_i = \sigma^{-1}(\tau^T z_i)$, where σ was the logit function and

[1] There are several types of GLM residuals, including response (or "raw") residuals, Pearson residuals, deviance residuals, and standardized (or "internally studentized") residuals. We examined the simplest type of residual — namely, the raw residual — in this analysis. We internally performed comparisons to the other types of residuals as well, and we obtained broadly similar results (not shown).

$\tau \in \mathbb{R}^3$ a fixed constant. Thus, the treatment x_i and covariate z_i were correlated, putting us into the *confounded* (as opposed to *unconfounded*) problem setting.

We then simulated gene expression data on $n_{\text{genes}} = 500$ genes. For a given gene $j \in \{1, \ldots, n_{\text{genes}}\}$, we sampled UMI counts $y_1^j, \ldots, y_n^j$ from an NB GLM model:

$$\begin{cases} y_i^j \sim \text{NB}_{\theta_j}(\mu_i^j), \\ \log(\mu_i^j) = \beta^T z_i + \gamma_j x_i, \end{cases} \tag{9}$$

where $\beta^T \in \mathbb{R}^p$ was a fixed regression coefficient, $\theta_j > 0$ was a gene-specific NB size parameter, and $\gamma_j \in \mathbb{R}$ was a gene-specific treatment effect. To simulate variation in overdispersion across genes, we sampled $\theta_1, \ldots, \theta_{n_{\text{sim}}}$ from a Unif$(0.1, 5)$ distribution. Additionally, to simulate variation in response to the treatment, we set the gene-specific treatment effect γ_j to 0 for 450 (randomly-selected) genes, and we set γ_j to a small, positive constant for the remaining 50 genes. Thus, for 450 (or 90% of) genes, we simulated data from the *null* model (in which treatment and response *were not* associated), and for 50 (or 10% of) genes, we simulated data from an *alternative* model (in which treatment and response *were* associated).

We applied three methods to analyze the data: a permutation test based on the difference-in-residual-means statistic T_{resid}, a permutation test based on the score test statistic T_{score}, and a GLM likelihood ratio test. We employed the adaptive permutation testing scheme and skew-normal approximation of SCEPTRE (low-MOI) to accelerate the two permutation-based methods (see Acceleration 3: Adaptive permutation testing and Acceleration 4: Skew-normal fit in the Methods section for details). Additionally, to simplify comparison of the methods, we treated the NB size parameter θ_j as fixed and known. (We estimated the NB size parameter in the real data analysis; see next section.) The GLM likelihood ratio test consisted of comparing the full model (9) to the reduced model that results from setting γ_j to zero in (9). We note that the GLM likelihood ratio test was optimal in the simulation study setting (i.e., the setting in which the GLM is correctly specified and the sample size is sufficiently large so as to enable accurate asymptotic inference). The T_{resid}-based permutation test and T_{score}-based permutation test were applied with two-sided test statistics so as to enable a meaningful comparison with the GLM likelihood ratio test, which is an inherently two-tailed test.

We assessed the degree of concordance between the T_{score}-based permutation test p-values, the T_{resid}-based permutation test p-values, and the GLM likelihood ratio test (LRT) p-values. As the GLM LRT p-values were optimal, we looked for agreement between the GLM LRT p-values and the p-values outputted by the two permutation methods. First, we plotted the GLM LRT p-values against the T_{resid}-based permutation test p-values (Figure S11a). Each point in the plot represents one of the 500 hypotheses tested; the red (resp., blue) points correspond to the null (resp., alternative) hypotheses. The vertical (resp., horizontal) position of a given point indicates the GLM LRT (resp., T_{resid}-based permutation test) p-value computed on that hypothesis. The p-values were negative log-transformed to facilitate visualization of small p-values. We observed that the T_{resid}-based p-values were strongly and positively correlated with the GLM LRT p-values. However, the T_{resid}-based p-values were generally larger (i.e., less significant) than the GLM LRT p-values. We regressed the GLM LRT p-values onto the T_{resid}-based p-values (via OLS) and superimposed the line of best fit onto the plot (dashed orange line). The line of best fit clearly lay above the identity line (solid black line), indicating deflation of the T_{resid}-based p-values relative to the GLM LRT p-values.

Next, we plotted the GLM LRT p-values against the T_{score}-based permutation p-values (Figure S11b). The two sets of p-values were highly concordant, as evidenced by the tight clustering

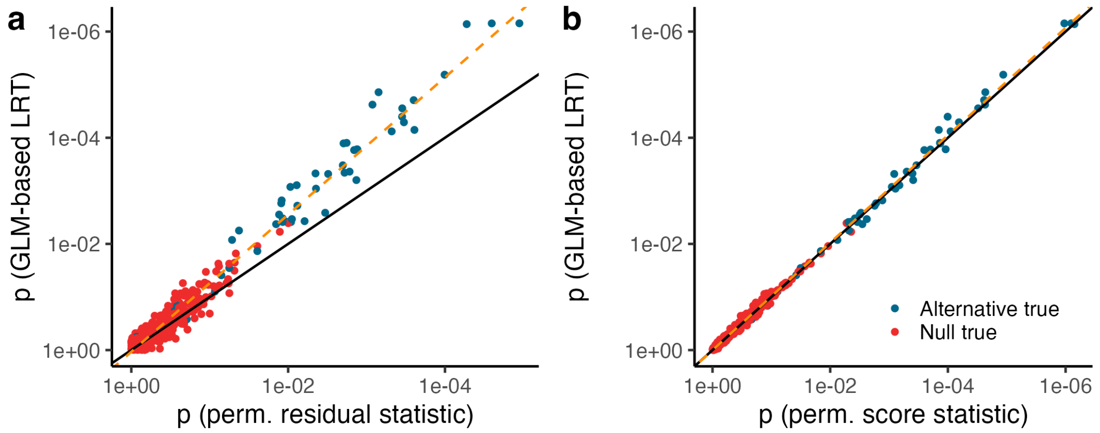

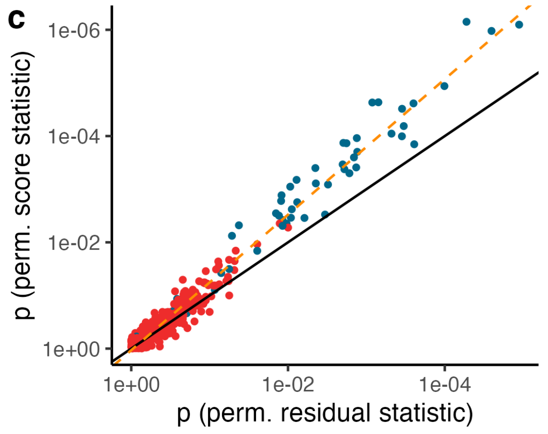

d

	GLM-based LRT	Perm. test with residual statistic	Perm. test with score statistic
Mean FDP (nominal level 10%)	9.0%	2.6%	9.0%
Mean N discoveries	36.6	23.9	36.3
Mean running time	0.021 s	0.012 s	0.022 s

Figure S11: **A comparison of the T_{score} and T_{resid} permutation test statistics on data simulated from an NB GLM.** **a**, GLM likelihood ratio test (LRT) p-values vs. T_{resid}-based permutation test p-values. The T_{resid}-based p-values were generally larger (i.e., more conservative) than the GLM LRT p-values. **b**, GLM LRT p-values vs. T_{score}-based permutation test p-values. These two sets of p-values were highly concordant. **c**, T_{score}-based permutation test p-values vs. T_{resid}-based permutation test p-values. The T_{resid}-based permutation test p-values were generally more conservative than the T_{score}-based permutation test p-values. **d**, mean false discovery proportion, number of discoveries, and running time of the GLM LRT, T_{resid}-based permutation test, and T_{score}-based permutation test across 500 replicates of the experiment. The GLM LRT and T_{score}-based permutation test exhibited near-identical statistical and computational performance. The T_{resid}-based permutation test exhibited lower power than the former two methods.

of the points around the identity line (solid black line). Furthermore, the line of best fit (solid orange line) closely tracked the identity line, indicating a high degree of agreement between the two sets of p-values. Finally, we plotted the T_{score}-based permutation p-values against the T_{resid}-based permutation p-values (Figure S11c). As expected, the T_{score}-based p-values were generally

smaller (i.e., more significant) than the T_{resid}-based p-values. We concluded that the T_{score}-based permutation p-values demonstrated a higher degree of concordance with the GLM LRT p-values than did the T_{resid}-based permutation p-values.

Finally, we subjected the three sets of p-values (i.e., those produced by the T_{score}-based permutation test, the T_{resid}-based permutation test, and the GLM LRT) to a BH correction at level 10%, obtaining a discovery set for method. The GLM LRT yielded 41 discoveries, two of which were false discoveries (false discovery proportion = 4.9%); next, the T_{score}-based permutation test produced 42 discoveries, two of which were false discoveries (false discovery proportion = 4.7%); finally, the T_{resid}-based permutation test yielded 25 discoveries and zero false discoveries (false discovery proportion = 0%). All three methods controlled the false discovery proportion at the nominal level of 10%. However, the T_{score}-based permutation test and GLM LRT yielded about 68% more discoveries than the T_{resid}-based permutation test.

To ensure robustness of the above results to choice of the random seed, we repeated the above experiment 500 times (Figure S11d). Averaged across runs, the GLM LRT, the T_{score}-based permutation test, and the T_{resid}-based permutation test yielded a mean false discovery proportion of 9.0%, 9.0%, and 2.6%, respectively. (The mean false discovery proportion functioned as an estimate for the false discovery rate.) Thus, all methods controlled the false discovery rate at the nominal level of 10%.[2] Next, the GLM LRT, the T_{score}-based permutation test, and the T_{resid}-based permutation test yielded a mean of 36.6 discoveries, 36.3 discoveries, and 23.9 discoveries, respectively. Hence, the GLM LRT and T_{score}-based permutation test produced about 53% more discoveries than the T_{resid}-based permutation test. Finally, the GLM LRT, the T_{score}-based permutation test, and the T_{resid}-based permutation test had a mean per-hypothesis compute time of 0.021 s, 0.022 s, and 0.012 s, respectively. Thus, the GLM LRT and T_{score}-based permutation test demonstrated similar running times, while the T_{resid}-based permutation test was roughly twice as fast.

In summary the GLM LRT and the T_{score}-based permutation test exhibited nearly identical performance in the simulation study: the two methods controlled type-I error at the same rate, made virtually the same number of discoveries, and had essentially the same running time. The T_{resid}-based permutation test, on the other hand, was considerably less powerful than either the GLM LRT or the T_{score}-based permutation test. We highlight that — in principle — the T_{score}-based permutation test can retain validity in a much broader range of settings than the standard GLM LRT, such as settings in which the data are sparse or the NB model is misspecified. We conjecture that the T_{score}-based permutation test trades a small loss in power for a substantial improvement in robustness relative to a standard GLM LRT. Additional work remains to be done to understand this phenomenon.

Real data analysis. We applied both the T_{score}-based permutation test and the T_{resid}-based permutation test to the Frangieh IFN-γ data. Similar to the simulation study, both methods controlled type-I error, while the T_{score}-based test produced a larger discovery set than the T_{resid}-based test. We analyzed three sets of perturbation-gene pairs: negative control pairs, positive control pairs, and discovery pairs. The discovery pairs consisted of the entire set (i.e., the *trans* set) of perturbation-gene pairs (that passed pairwise QC). The negative control pairs were constructed by the SCEPTRE software and were "matched" to the discovery pairs.[3] In particular, each negative control target consisted of three, randomly-grouped non-targeting gRNAs. (In this sense the calibration check analysis reported here is not precisely the same as the calibration check analysis

[2] We conjectured that the T_{resid}-based permutation test controlled type-I error due to the CAMP phenomenon.

[3] See `timothy-barry.github.io/sceptre-book/run-calibration-check.html` for details.

reported in Figure 4.) Finally, the positive control pairs consisted of perturbations targeting gene transcription start sites paired to the genes regulated by those transcription start sites.

Initially, the p-values outputted by the T_{score}-based method and the T_{resid}-based method exhibited mild miscalibration on the negative control data: the former method yielded five false discoveries, and the latter method yielded three false discoveries (at BH level 0.1). Therefore, we slightly increased the stringency of the cellwise QC thresholds, removing cells whose percent mitochondrial value exceeded 0.1 or whose number of genes expressed or total gene UMI count fell outside of the [0.1, 0.99] percentile range. We plotted the resulting T_{score}-based p-values against the T_{resid}-based p-values computed on the negative control pairs (S12a), positive control pairs (S12b), and discovery pairs (S12c). We subjected both the T_{score}-based p-values and the T_{resid}-based p-values to a BH correction (separating out the negative control pairs, positive control pairs, and discovery pairs). We varied the nominal FDR level over the grid $\{0.1, 0.05, 0.01, 0.005\}$ to explore how increasing the stringency of the multiplicity correction might affect the results. The T_{score}-based negative control p-values and T_{resid}-based negative control p-values were roughly symmetrically distributed about the identity line (S12a). Moreover, both the T_{score}-based test and T_{resid}-based test controlled type-I error on the negative control pairs, as each method produced zero or one false discoveries at the various BH levels (S12d).

Next, we observed that the positive control T_{score}-based p-values and T_{resid}-based p-values were highly correlated (S12b). However, the T_{score}-based p-values were generally smaller (i.e., more significant than) their T_{resid}-based counterparts, hinting at the greater power of the T_{score} test statistic. Finally, the discovery T_{score}-based p-values and T_{resid}-based p-values also were highly correlated, and again, the T_{score}-based p-values were generally smaller than their T_{resid} counterparts, especially in the tail of the p-value distribution. Moreover, the T_{score}-based method yielded a larger discovery set than the T_{resid}-based method over the various nominal FDR levels. The relative difference between the size of the of the T_{score}-based discovery set and T_{resid}-based discovery set increased as the BH correction became more stringent. For example, at BH level 0.1, the T_{score}-based method yielded 274 discoveries, a 3% improvement over the T_{resid}-based method, which made 266 discoveries. However, at BH level 0.005, the T_{score}-based method produced 112 discoveries, a 29% improvement over the T_{resid}-baesd method, which made 87 discoveries (Figure S12d).

We repeated this analysis on the Papalexi gene expression data, obtaining broadly similar results: the T_{score}-based and T_{resid}-based methods both controlled type-I error on the negative control data, but the T_{score}-based method produced slightly smaller p-values on the positive control pairs, and the T_{score}-based method yielded a larger discovery set (by about 3%) on the discovery pairs. Overall, synthesizing the results of the simulation study, the Frangieh data analysis, and the Papalexi data analysis, we concluded that the T_{score} statistic and T_{resid} statistic can yield quite different results. In particular, depending on the dataset under analysis and the stringency of the multiple testing adjustment, the T_{score} statistic can produce a discovery set up to 50% larger than the one produced by the T_{resid} statistic.

Comparing the spectral decomposition algorithm to the QR decomposition algorithm for computing GLM score tests

We proposed a spectral decomposition algorithm for computing GLM score tests within the context of SCEPTRE (see Acceleration 2: A fast score test for binary treatments). The spectral decomposition approach differs from the standard approach for computing GLM score tests; the latter is based on a QR decomposition. We conducted a small computational experiment to compare

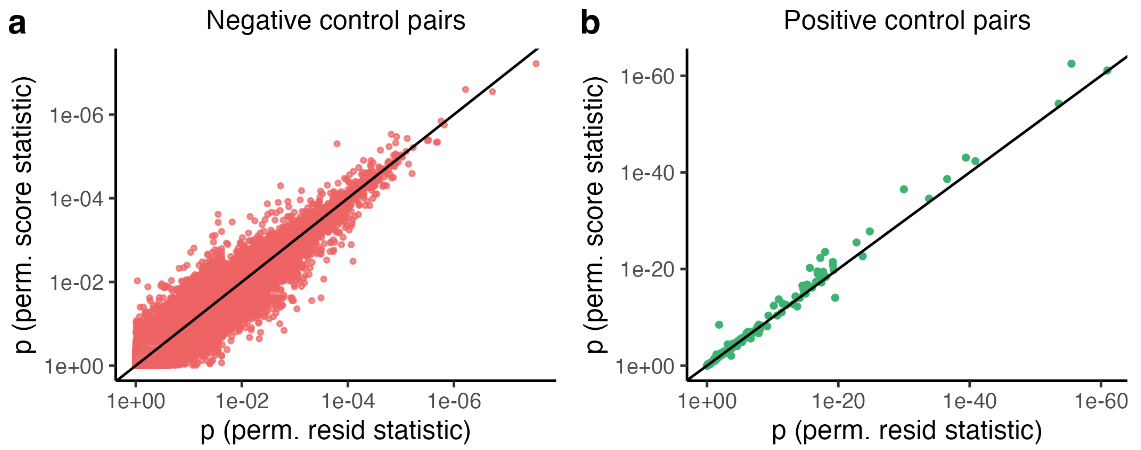

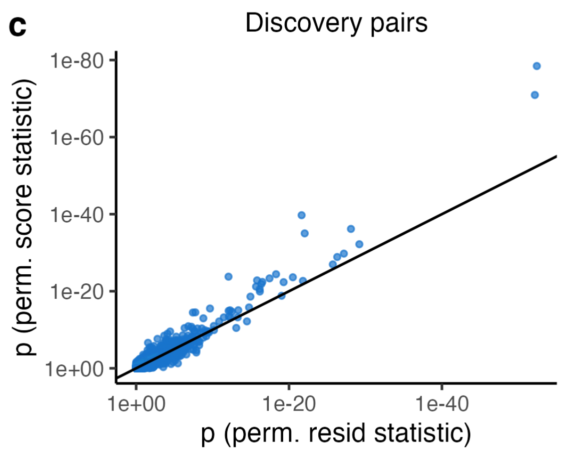

d

N (false) rejections on negative control pairs

Nominal FDR:	0.1	0.05	0.01	0.005
Residual	1	0	0	0
Score	0	0	0	0

N rejections on discovery pairs

Nominal FDR:	0.1	0.05	0.01	0.005
Residual	266	189	107	87
Score	274	207	126	112
Percent increase	3.0%	9.5%	17.8%	28.7%

Figure S12: **A comparison of the $T_{\textbf{score}}$ and $T_{\textbf{resid}}$ permutation test statistics on the Frangieh IFN-γ data.** T_{score}-based p-values vs. T_{resid}-based p-values on the negative control (**a**), positive control (**b**), and discovery (**c**) pairs. **d**, number of rejections made by the T_{resid}-based method (top row) and T_{score}-based method (bottom row) on the negative control (top table) and discovery (bottom table) pairs. The various nominal FDR levels — 0.1, 0.05, 0.01, and 0.005 — are in the columns.

the speed of the spectral decomposition algorithm to that of the QR decomposition algorithm. We found that the former was faster for sparse, binary treatment vectors. Furthermore, as we increased the sparsity level of the treatment vector, we found that the speed of the spectral decomposition algorithm relative to that of the QR decomposition algorithm increased.

We generated synthetic data on $n = 100,000$ cells, drew the covariates $z_1, \ldots, z_n$ from a bivariate Gaussian distribution, and generated the gene expression counts from a negative binomial model:

$$y_i \sim \text{NB}_\theta(\mu_i) \quad \log(\mu_i) = \beta^T z_i.$$

We regressed the gene expression counts onto the covariates, obtaining a fitted GLM `fit`.

Sparsity	Spectral decomp. running time	QR decomp. running time	Speedup
99.9%	$0.0057s$ (0.0056, 0.0058)	$1.55s$ (1.55, 1.56)	272
99%	$0.0088s$ (0.0087, 0.0089)	$1.60s$ (1.60, 1.60)	181
95%	$0.023s$ (0.023, 0.023)	$1.62s$ (1.61, 1.62)	71
90%	$0.040s$ (0.040, 0.040)	$1.61s$ (1.61, 1.61)	40
50%	$0.147s$ (0.146, 0.147)	$1.62s$ (1.62, 1.62)	11

Table S4: **Benchmark comparison of the spectral decomposition algorithm and the QR decomposition algorithm for computing GLM score tests**. Left column, sparsity level of the treatment vector (i.e, mean fraction of entries within the treatment vector equal to zero). Middle two columns, mean running time of the spectral decomposition method and QR decomposition method (95% confidence intervals in parentheses). Right column, "speedup" (i.e., the QR decomposition running time divided by the spectral decomposition running time).

Next, for a given sparsity level $\pi \in \{0.999, 0.99, 0.95, 0.90, 0.50\}$, we generated 1000 synthetic treatment vectors $x^1, \ldots, x^{1000}$ (each of length n) by sampling i.i.d. variates from a Bern$(1 - \pi)$ distribution. We tested each treatment vector for inclusion in the fitted GLM `fit` by running a GLM score test. We evaluated two different methods for computing the GLM score test: the spectral decomposition approach (proposed in this work), and the standard QR decomposition approach. We used the implementation of the QR decomposition approach available within the `statmod` [1] R package, which is a fast and high-quality implementation of the method. To ensure robustness of the results to choice of the random seed, we repeated the experiment 50 times for each choice of the sparsity level π, generating fresh covariates $z_1, \ldots, z_n$ and gene expression counts $y_1, \ldots, y_n$ for each run of the experiment.

We found that the spectral decomposition algorithm was much faster than the QR decomposition algorithm (Table S4). For example, at sparsity level 99.9%, the mean running time of the spectral decomposition algorithm was $0.0057s$ (95% CI: ($0.0056s$, $0.0058s$)), while that of the QR decomposition algorithm was $1.55s$ (95% CI: ($1.55s$, $1.56s$)), indicating a speedup by a factor of 272. Consistent with theory, the relative efficiency of the spectral decomposition algorithm in comparison to the QR decomposition algorithm decreased as the sparsity level decreased. For example, at sparsity level 50%, the spectral decomposition algorithm was only 11 times faster than the QR decomposition algorithm. We concluded that the spectral decomposition algorithm is well-suited to computing GLM score tests for sparse, binary treatment vectors.

Experimental design considerations

A question that an experimenter might ask is as follows: "How many cells per perturbation do I need if I plan to use SCEPTRE to analyze my data?" We sought to provide a preliminary answer to this question, leveraging our results on the calibration and power of SCEPTRE to guide our analysis. Consider a given perturbation-gene pair. Let n_{trt} be the number of cells containing the targeting perturbation. We refer to the number of cells containing *both* the targeting perturbation *and* nonzero expression of the gene as the "effective sample size" or the "number of nonzero treatment cells" for that pair. The SCEPTRE software filters out pairs for which the effective sample size falls below some threshold T. (By default, T is set to 7, a choice informed by the calibration (Figure S4 - S5) and power (Figure S9) results of SCEPTRE at different effective sample sizes.) Finally, let s denote the sparsity of a given dataset, i.e., the fraction of entries in the gene-by-cell expression

matrix equal to zero.

We can model the effective sample size X of a given pair using a binomial model:

$$X \sim \text{Bin}(n_{\text{trt}}, 1-s).$$

Among the n_{trt} cells that contain the perturbation, each exhibits nonzero expression of the gene with probability $1-s$. Thus, the number of cells containing *both* the perturbation *and* nonzero expression of the gene — i.e., the effective sample size — is binomially distributed with parameters n_{trt} and $1-s$. The probability that X equals $x \in \{0,\ldots,n_{\text{trt}}\}$ is given by the binomial pmf:

$$\mathbb{P}(X = x) = \binom{n_{\text{trt}}}{x}(1-s)^x s^{n_{\text{trt}}-x}.$$

The pair passes pairwise QC only if $X \geq T$, i.e., the effective sample size is greater than or equal to the pairwise QC threshold. Let π be the probability that the pair passes pairwise QC. (One should think of π as a parameter specified by the experimenter, like 0.9.) Finally, let $n_{\text{trt}}(\pi, T, s)$ be the number of treatment cells required for the pair to pass pairwise QC with probability π at pairwise QC threshold T on a dataset of sparsity s. We can express $n_{\text{trt}}(\pi, T, s)$ mathematically as

$$n_{\text{trt}}(\pi, T, s) = \min\left\{ n \in \{1, 2, 3, \ldots\} : \sum_{x=T}^{n} \binom{n}{x}(1-s)^x s^{n-x} \geq \pi \right\}.$$

In other words $n_{\text{trt}}(\pi, T, s)$ is the smallest integer n such that the right-tail probability of $\text{Bin}(n, 1-s)$ evaluated T is greater than or equal to π. It is challenging to derive an exact expression for $n_{\text{trt}}(\pi, T, s)$, but we can evaluate $n_{\text{trt}}(\pi, T, s)$ numerically. In summary, suppose that the sparsity level of the dataset is s, that the pairwise QC threshold is set to T, and that the desired probability that a given pair passes pairwise QC is π. Then the experimenter should ensure that each perturbation is contained within at least $n_{\text{trt}}(\pi, T, s)$ cells.

We evaluated $n_{\text{trt}}(\pi, T, s)$ for different values of π, T, and s, plotting n_{trt} as a function of π (Figure S13). We set the sparsity level s by computing the sparsity of the datasets analyzed in this work. The four "standard" datasets with gene expression readout — i.e., Frangieh co-culture, Frangieh control, Frangieh IFN-γ, and Papalexi (gene modality) — exhibited sparsity levels of 0.76, 0.77, 0.80, and 0.77, respectively (mean sparsity level 0.78). Next, the two TAP-seq datasets — i.e., Schraivogel chromosome 11 and Schraivogel chromosome 8 — exhibited sparsity levels of 0.33 and 0.27, respectively (mean sparsity level 0.30). Finally, the Papalexi (protein modality) dataset exhibited a sparsity level of 0.0. Thus, we set the sparsity s to 0.77, 0.30, and 0.0, corresponding to a "standard" screen with gene expression readout, a TAP-seq screen with gene expression readout, and a screen with protein expression readout.

We plotted the number of required treatment cells $n_{\text{trt}}(\pi, T, s)$ as a function of the probability π that a given pair passes pairwise QC (Figure S13). The left (resp., right) panel shows $n_{\text{trt}}(\pi, T, s)$ for an effective sample size QC threshold T of 7 (resp., 14). (The default threshold that the SCEPTRE software uses is 7.) Finally, the colors within each plot indicate the different sparsity levels: 0.78 (red), 0.30 (purple), and 0.0 (blue). The visual Figure S13 can be used to help guide experimental design. For example, suppose that an experimenter is designing a screen in which she plans to sequence gene transcripts using the standard single-cell sequencing protocol. (That is, the experimenter is not using the TAP-seq protocol.) Based on prior single-cell CRISPR screens datasets, the experimenter expects the sparsity of the dataset to be about 0.78. Moreover, suppose

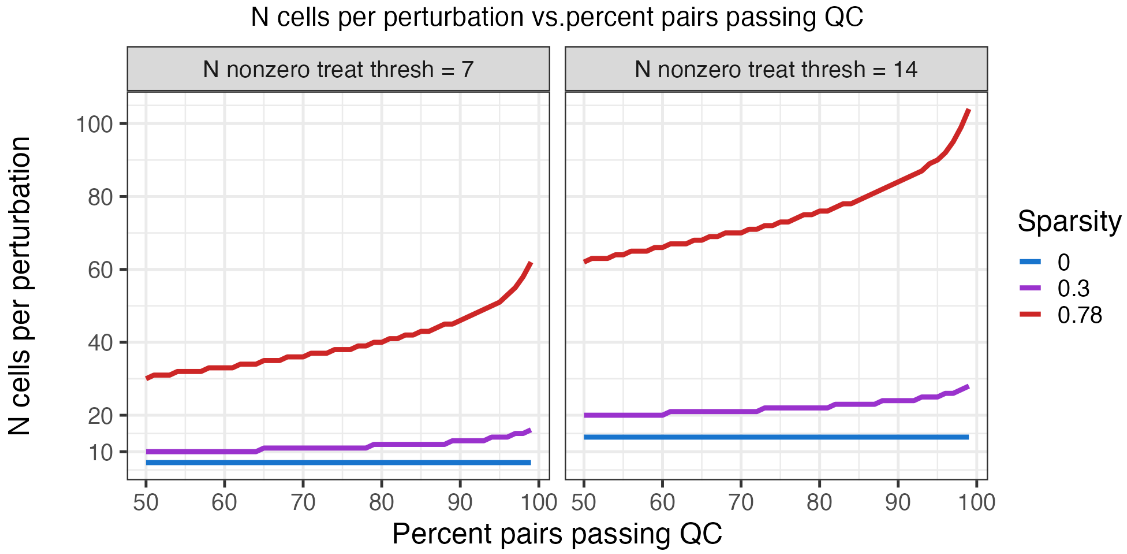

Figure S13: **Number of cells per perturbation vs. probability that a given pair passes pairwise QC.** A plot of the number of cells required per perturbation ($n_{\text{trt}}(\pi, T, s)$) vs. the probability that a given pair passes pairwise QC (π). The left (resp., right) plot considers setting the pairwise QC threshold T to 7 (resp., 14). Finally, the colors indicate different sparsity levels s: red = 0.78, purple = 0.30, and blue = 0.0.

that the experimenter intends to use the default SCEPTRE pairwise QC threshold of $T = 7$. Finally, suppose that experimenter aims for 95% of the pairs to pass pairwise QC. Then the experimenter should try to ensure that each perturbation is contained within at least $n_{\text{trt}}(0.95, 7, 0.95) = 50$ cells. As a very rough, general guideline, experimenters probably should ensure that each perturbation is contained within at least 50-80 cells. We (i.e., the authors) intend to add functionality to the SCEPTRE software in the future to aid users in designing single-cell CRISPR screen experiments, including a module for determining the approximate number of cells required per perturbation.

We note that the simple model considered here does not account for more complex phenomena present in single-cell CRISPR screen data. For example, cells containing multiple perturbations typically are removed as part of cellwise QC in low MOI, thereby decreasing the sample size. Moreover, sparsity can vary considerably across genes, as some genes are more highly expressed or more deeply sequenced than others. We believe that the analysis above constitutes a solid starting point for reasoning about experimental design in the context of SCEPTRE; however, additional further investigation remains to be done in a followup work.

References

[1] Dunn, P. K. & Smyth, G. K. *Generalized linear models with examples in R*, chap. 7, 286–287 (Springer, 2018).
