# Supplementary material for: Robust differential expression testing for single-cell CRISPR screens at low multiplicity of infection
Source: Genome Biol. 2024 May 17;25:124. doi: 10.1186/s13059-024-03254-2 (PMC11100084; doi:10.1186/s13059-024-03254-2)
Supplement: Supplementary file 2 — Additional file 2. Review history. [file 13059_2024_3254_MOESM2_ESM.docx]

Review History

**First round of review**

**Reviewer 1**

**Were you able to assess all statistics in the manuscript, including the appropriateness of statistical tests used?** Yes.

**Were you able to directly test the methods?** No.

**Comments to author:**

The authors address a core and surprisingly difficult problem in perturb-seq data: how to decide if a given difference in gene expression is real. The existing approaches often fail to control false positives, generally because the model is misspecified in some important respect. As a researcher who has dealt with this problem, I much appreciate the authors for developing a performant solution and establishing a solid framework to test its validity. SCEPTRE (low MOI) combines negative binomial regression with cell label permutation to test the null hypothesis that a gene's expression has the same mean before and after perturbation. The intuition is simple enough and the method is clearly described. Critically, the authors have put considerable effort into creating a efficient, tractable implementation of the permutation testing. If this had been implemented naively, it would have been completely unfeasible for typical single cell data sets.

The authors compare their method to major competitors on two sets of real single cell data. I found their negative control test convincing, and as the authors state, a good framework overall for testing false positive control. The positive control test was even more striking, although somewhat limited--it is difficult to identify solid positive controls in this data type. I appreciate as well that the authors took the time to explore the causes of misspecification in rival methods. Overall, the paper makes a very strong case that SCEPTRE is superior to current methods. Additionally, I hope the paper will encourage (or shame) future authors into conducting a similar validation and reduce the flood of elaborate but useless single cell methods.

I had three major comments/questions. #3 is the most critical to address.

1. The authors adopted a number of tricks to make repeated NB regression tractable. But I wonder: is there a big difference in performance (in terms of sensitivity/specificity) vs. if one just regressed out the confounding variables from the matrix, then directly tested the null that the gene expression had the same mean in the two cell populations? I.e. take as your statistic the difference in means, and develop the null distribution of differences in means over the permutations.

2. There's a mismatch between how strongly the authors state their robustness claims (CAMP) and how much evidence is provided. To justify the strong guarantees stated in the Methods, the authors should really need to present a proof, rather than a single example in which the model appears to tolerate artificial deviations well. Without a proof, I'd suggest rephrasing to "We tested the model under these conditions, and found it robust."

3. The authors' description of "Inductive without replacement sampling" makes it sound like they are sampling from the same set of treatment cells for the permutations to test ALL treatment-gene associations: "Thus, if a given gene-perturbation pair contains k ≤ M treatment cells, then Ak constitutes a valid WOR sample for that pair." Ak appears to be a permutation using a fixed set of treatment cells.

In general, it is not valid to use samples drawn from (potentially) a completely different distribution X to test the null hypothesis that distributions Y and Z have the same mean. For example, suppose that gRNA A induces large increase in gene G's expression. Permutations using A-infected cells will have systematically increased variance in G due to randomly mixing in these high-expressing cells. If these permutations are used to test for significance with gRNA B, which might produce weaker changes in G, it can lead to a pessimistic p-value for the B-G association. Alternatively, if G has lower dispersion in gRNA A cells than gRNA B, using A-treated cells could lead to an optimistic p-value for the B-G association.

It's important to note that the analyses the authors use are unlikely to detect misspecification due to using the wrong treatment cells in the permutations, since the most robust test they perform is based on pairs of negative controls which should be drawn from the same expression distributions (modulo confounding). Can the authors explain why this sampling strategy does not result in misspecification for the case of different treatments? Can they make plots comparing the p-values calculated using permutations of the correct treatment and control vs permutations using the treatment with most cells across gene/targeting sgRNA pairs? Alternatively, if I've misunderstood the sampling strategy, it would be helpful if they could clarify that section.

Minor points:

1. In the Methods, line 42 "This formula is derived in Section ."

2. In the derivation of the score statistic, line 18, should the augmented matrix D have shape n x p+1?

3. Line 8: the reference to Figure 4c should be to 4d

4. Line 17: "We used publicly available ChIP-Seq data"--could the authors cite the data?

5. The authors cite but don't evaluate scMaGECK. Can they explain why in the "Methods not included" section?

6. Reference 27 used an Anderson-Darling test on z-scored expression changes IIRC, not KS

**Reviewer 2**

**Were you able to assess all statistics in the manuscript, including the appropriateness of statistical tests used?** Yes.

**Were you able to directly test the methods?** No.

**Comments to author:**

This manuscript presents an elegant study to explore differential expression testing for single-cell CRISPR screens, which is a core problem in the data analysis of single-cell CRISPR screens. As this technology becomes commonly adapted in various fields, addressing this core problem is definitely very critical in terms of statistical rigor and robustness. Through benchmarking, authors reveal that existing methods do not perform well due to three different analysis challenges, and further propose a method (SCEPTRE-low-MOI) to address these challenges. This method has been benchmarked with existing methods, and overcomes the current three limitations.

Overall this is a well-performed study that addresses a critical problem in the field. The statistical aspect of this method is novel and robustness. A few issues are identified and mentioned below.

Major:

- systematic identification of core analysis challenges. This section is definitely very important and thanks the authors for performing detailed analysis. This section is currently very brief and can be expanded to address the following:

1. Sample size: this is a very important point to address the limitation of existing methods and is very informative on the experimental design (e.g., how many cells per perturbation are needed to avoid p value inflation). Maybe authors can perform additional analysis to inform the experimental design.

2. Sample size: right now only 2 datasets are tested (Fig. 2 and S5) and the performance of existing methods is very different. Therefore adding 1-2 more datasets for evaluation is recommended

3. Technical factors: Figure 2d is a little but confusing as I don't quite get how differences in biological replicates affect the results? Maybe authors show the results for R1/R2/R3 separately, and in comparison with the results merging three replicates together?

4. Technical factors: Fig S6 is difficult to understand. More explanation is needed to show how replicates affect the QQ plot

- positive control data (page 7, line 51). A more description is needed here in terms of what pairs are considered positive. TSS guides are easy to understand. For enhancers: identifying enhancer-gene pairs is a very challenging task, therefore I'm not sure how to define "known" enhancers here.

1. A related thought is, since authors already use synthetic data, generating synthetic positive perturbation-gene pairs would be a better way for evaluating sensitivity?

- discovery analysis (page 9, last paragraph before Discussion). The IRF1/STAT1 analysis is rudimentary and requires further analysis. For example, how is SCEPTRE compared with other methods for identifying the enrichment of IRF1/STAT1 targets using existing ChIP-seq? Also authors mention Papalexi and Frangieh datasets are used, however only IRF1/STAT1 (from Papalexi dataset) are mentioned. How about the results in the Frangieh dataset?

Minor:

- page 7, line 24: "hundreds of times faster" -- is there a figure/table to actually demonstrate the author's claims?

**Authors Response**

**Point-by-point responses to the reviewers’ comments:**

*We thank the editor for the opportunity to complete a major revision. Please find our response to reviewers in the file "response_to_reviewers.pdf." We paste the contents of our response below.*

*=============================================*

*> In this document we provide a detailed account of the changes that we have made to the manuscript. We also respond to the reviewers’ comments line-by-line. The reviewers’ comments are unindented, and our responses are indented with a ">" symbol. We thank both reviewers and the editor for their thoughtful and insightful feedback, which considerably strengthened the manuscript.*

Reviewer #1: The authors address a core and surprisingly difficult problem in perturb-seq data: how to decide if a given difference in gene expression is real. The existing approaches often fail to control false positives, generally because the model is misspecified in some important respect. As a researcher who has dealt with this problem, I much appreciate the authors for developing a performant solution and establishing a solid framework to test its validity. SCEPTRE (low MOI) combines negative binomial regression with cell label permutation to test the null hypothesis that a gene's expression has the same mean before and after perturbation. The intuition is simple enough and the method is clearly described. Critically, the authors have put considerable effort into creating a efficient, tractable implementation of the permutation testing. If this had been implemented naively, it would have been completely unfeasible for typical single cell data sets.

The authors compare their method to major competitors on two sets of real single cell data. I found their negative control test convincing, and as the authors state, a good framework overall for testing false positive control. The positive control test was even more striking, although somewhat limited--it is difficult to identify solid positive controls in this data type. I appreciate as well that the authors took the time to explore the causes of misspecification in rival methods. Overall, the paper makes a very strong case that SCEPTRE is superior to current methods. Additionally, I hope the paper will encourage (or shame) future authors into conducting a similar validation and reduce the flood of elaborate but useless single cell methods.

*> We thank the reviewer for the positive and thoughtful comments.*

*> We briefly respond to the issue related to the positive control pairs. The original manuscript contained 584 positive control pairs. Of these, only five (or <1%) were enhancer-gene pairs; the rest were TSS (transcription start site)—gene pairs. Enhancer-gene pairs are less reliable positive controls than TSS-gene pairs, as enhancer-gene links are harder to validate than TSS-gene links. Thus, to ensure that the positive control pairs were of uniformly high quality, we removed the five enhancer-gene pairs from the set of positive control pairs, leaving only the 579 TSS-gene pairs. The positive control results remained essentially the same after we made this change. Furthermore, we applied the methods to analyze a new simulated positive control dataset in which the ground truth was known (Figure 4c).*

I had three major comments/questions. #3 is the most critical to address.

The authors adopted a number of tricks to make repeated NB regression tractable. But I wonder: is there a big difference in performance (in terms of sensitivity/specificity) vs. if one just regressed out the confounding variables from the matrix, then directly tested the null that the gene expression had the same mean in the two cell populations? I.e. take as your statistic the difference in means, and develop the null distribution of differences in means over the permutations.

*> We agree that this is an interesting and natural question to ask. We conducted an in-depth exploration of this question in the newly-added section “Comparing the score statistic to the difference-in-residual means statistic” (pg 55), which is a part of Supplement 3 (“Additional analyses”). We compared the NB GLM score statistic to the statistic formed by taking the difference in means of the NB GLM residuals over the “treatment cells” and the “control cells.” We compared these two statistics on simulated data and two real datasets (namely, the Papalexi data and Frangieh data). We found that, while both statistics controlled type-I error, the score statistic was considerably more powerful than the difference-in-residual-means statistic. In particular, depending on the dataset under analysis and the stringency of the multiple testing adjustment, the score statistic produced a discovery set up to 50% larger in size than the one produced by the difference-in-residual-means statistic.*

*> We added a reference to this additional analysis in the main text (pg 6): “A natural question is whether SCEPTRE is better (in some sense) than the simpler method that entails `regressing out’ the technical factors via an NB GLM, extracting the residuals from the fitted GLM, and then performing a permutation test on the residuals (taking, for example, the difference in means across treatment and control groups as the test statistic). We answered this question in the affirmative, finding that SCEPTRE was considerably more powerful than the residual-based method on both real and simulated data. (See section Comparing the score statistic to the difference-in-residual-means statistic.)”*

2. There's a mismatch between how strongly the authors state their robustness claims (CAMP) and how much evidence is provided. To justify the strong guarantees stated in the Methods, the authors should really need to present a proof, rather than a single example in which the model appears to tolerate artificial deviations well. Without a proof, I'd suggest rephrasing to "We tested the model under these conditions, and found it robust."

*> We agree that we overstated the robustness claim in the initial version of the manuscript. We also agree that it would be useful to provide a proof of CAMP. However, in our view, proving CAMP falls outside the scope of the current manuscript, which is already quite involved. We therefore updated the “Statistical robustness property” portion of the Methods section (p 30) as follows.*

*> “SCEPTRE empirically demonstrated a robustness property that we term ``confounder adjustment via marginal permutations,'' or `CAMP.' We observed evidence of CAMP across our simulation studies (Figures 4, S9, and S12) and real data analyses. We describe CAMP in greater detail here. For simplicity we consider the version of SCEPTRE that does not involve fitting a skew-normal distribution to the null test statistics and instead computes the standard permutation test $p$-value by directly comparing the observed test statistic to the null test statistics. If at least one of the following conditions holds, we conjecture that the left-, right-, and two-tailed SCEPTRE p-values are valid: (i) the perturbation is unconfounded (i.e., the vector of technical factors $Z_i$ contains all possible confounders, and $Z_i$ is independent of $X_i$); (ii) the NB GLM (1) is correctly specified up to the size parameter $\theta$ and the effective sample size is sufficiently large. We reasoned that CAMP enabled SCEPTRE to address the core single-cell CRISPR screen analysis challenges of sparsity, confounding, and model misspecification both in theory and practice. We intend to derive a mathematical proof of CAMP in a followup, more theoretical work.”*

3. The authors' description of "Inductive without replacement sampling" makes it sound like they are sampling from the same set of treatment cells for the permutations to test ALL treatment-gene associations: "Thus, if a given gene-perturbation pair contains k ≤ M treatment cells, then Ak constitutes a valid WOR sample for that pair." Ak appears to be a permutation using a fixed set of treatment cells.

In general, it is not valid to use samples drawn from (potentially) a completely different distribution X to test the null hypothesis that distributions Y and Z have the same mean. For example, suppose that gRNA A induces large increase in gene G's expression. Permutations using A-infected cells will have systematically increased variance in G due to randomly mixing in these high-expressing cells. If these permutations are used to test for significance with gRNA B, which might produce weaker changes in G, it can lead to a pessimistic p-value for the B-G association. Alternatively, if G has lower dispersion in gRNA A cells than gRNA B, using A-treated cells could lead to an optimistic p-value for the B-G association.

It's important to note that the analyses the authors use are unlikely to detect misspecification due to using the wrong treatment cells in the permutations, since the most robust test they perform is based on pairs of negative controls which should be drawn from the same expression distributions (modulo confounding). Can the authors explain why this sampling strategy does not result in misspecification for the case of different treatments? Can they make plots comparing the p-values calculated using permutations of the correct treatment and control vs permutations using the treatment with most cells across gene/targeting sgRNA pairs? Alternatively, if I've misunderstood the sampling strategy, it would be helpful if they could clarify that section.

*> We did not explain IWOR sampling in the initial version of the manuscript in a sufficiently clear way. IWOR sampling involves carrying out an exact, provably correct permutation test for every hypothesis; it is an approximation-free technique. Furthermore, IWOR sampling does not involve sharing cells across hypotheses; rather, it involves sharing an “IWOR sample” — from which the set of permutation indices for a given hypothesis can be constructed — across hypotheses. To better explain IWOR sampling, we updated and clarified the introductory paragraph of the “Inductive without replacement sampling” section (pg 49). Furthermore, we added a new subsection, “A concrete example of IWOR sampling.” This subsection provides a worked example of IWOR sampling on a simple dataset. It also presents a visualization of IWOR sampling (Figure S11, pg 49), which helps to convey intuition for the procedure.*

*> We also added a reference to the supplementary section describing IWOR sampling in the main text (pg 6): “Finally, we developed a novel strategy --- ``inductive without replacement sampling'' --- for recycling compute across permutation tests in which each test contains the same number of control units. (See Inductive without replacement sampling for details.)”*

Minor points:

In the Methods, line 42 "This formula is derived in Section .”

*> Fixed*

2. In the derivation of the score statistic, line 18, should the augmented matrix D have shape n x p+1?

*> Yes. We updated the text to make the dimension of the vectors and matrices consistent throughout. $Z$ is the covariate matrix and has dimension $n \times p$. $Z$ includes a column of ones corresponding to the intercept. Next, $X$ is an n-dimensional vector of perturbation presences and absences. Finally, $D = [X, Z]$ is the matrix of dimension $n \times (p+1)$ that results from concatenating $X$ and $Z$.*

3. Line 8: the reference to Figure 4c should be to 4d

*> Fixed*

4. Line 17: "We used publicly available ChIP-Seq data"--could the authors cite the data?

*> We added the citation to this sentence in the main text (previously the reference was only in the Methods section).*

5. The authors cite but don't evaluate scMaGECK. Can they explain why in the "Methods not included" section?

*> We added the following text to the “Methods not included” section (pg 33): “Finally, scMaGECK (proposed by Yang(14)) tests for association between CRISPR perturbations and gene expressions using a permutation test with a linear regression coefficient test statistic. Given that we carefully evaluated the high-MOI version of scMaGECK in our prior work(13), and given that MIMOSCA also is based on a permutation test with a linear regression coefficient test statistic, we deprioritized scMaGECK for benchmarking.”*

6. Reference 27 used an Anderson-Darling test on z-scored expression changes IIRC, not KS

*> We thank the reviewer for catching this. The three major single-cell CRISPR screen papers from the Weissman Lab are Adamson 2016, Replogle 2020, and Replogle 2022. The first two studies used a KS test, while the latter study used a Mann-Whitney test as well as an Anderson-Darling test. We removed reference 27 from Table 1.*

Reviewer #2: This manuscript presents an elegant study to explore differential expression testing for single-cell CRISPR screens, which is a core problem in the data analysis of single-cell CRISPR screens. As this technology becomes commonly adapted in various fields, addressing this core problem is definitely very critical in terms of statistical rigor and robustness. Through benchmarking, authors reveal that existing methods do not perform well due to three different analysis challenges, and further propose a method (SCEPTRE-low-MOI) to address these challenges. This method has been benchmarked with existing methods, and overcomes the current three limitations.

Overall this is a well-performed study that addresses a critical problem in the field. The statistical aspect of this method is novel and robustness. A few issues are identified and mentioned below.

*> We thank the reviewer for the positive and thoughtful comments.*

Major:

systematic identification of core analysis challenges. This section is definitely very important and thanks the authors for performing detailed analysis. This section is currently very brief and can be expanded to address the following:

*> We thank the reviewer for the positive feedback on this section. We note that we described our exploration of the core analysis challenges in much greater detail in the Methods section (“Details of the investigation into the core analysis challenges”, pg 24). We added the following sentence to the main text to direct interested readers to the Methods section (pg 4): “This section is abbreviated to preserve space; interested readers can consult Details of the investigation into the core analysis challenges, which contains a more detailed description of the investigation into the analysis challenges.”*

Sample size: this is a very important point to address the limitation of existing methods and is very informative on the experimental design (e.g., how many cells per perturbation are needed to avoid p value inflation). Maybe authors can perform additional analysis to inform the experimental design.

*> We agree that the results that we obtained on the power and calibration of SCEPTRE can be used to inform questions related to experimental design, such as the number of cells required per perturbation. Thus, we added a new section — “Experimental design considerations” (pg 61) — to the supplementary materials. This section presents a simple strategy for determining the number of cells required per perturbation to ensure adequate calibration and power of SCEPTRE on a given dataset. The takeaway message is that — on a “standard” single-cell CRISPR screen dataset — the experimenter should try to ensure that each perturbation is contained within (at minimum) 50-65 cells.*

*> We also added the following text to the section “Pairwise quality control and experimental design” (pg 7) to refer interested readers to the supplementary analysis: “Additionally, we reasoned that our results on the power (Figure S10) and calibration (Figures S5-S6) of SCEPTRE might inform questions related to the experimental design of single-cell CRISPR screens, such as the number of cells per perturbation required to ensure adequate power and calibration of SCEPTRE on a given dataset. We conducted a supplementary analysis in which we derived a simple mathematical formula for the minimum number of cells that must contain each perturbation so as to ensure that a specified fraction of pairs passes pairwise QC, where the pairwise QC threshold was selected on the basis of SCEPTRE's calibration and power at different effective sample sizes. We concluded that, on a dataset of standard sparsity (e.g., the Frangieh IFN-$\gamma$ dataset or the Papalexi gene modality dataset), each perturbation should be contained within at least 50-65 cells to ensure that $95\%$ of pairs pass pairwise QC. (See Experimental design considerations for more details. Also, see also Morris et al.,\cite{Morris2023} who conducted a complementary power analysis in the high-MOI setting.)”*

2. Sample size: right now only 2 datasets are tested (Fig. 2 and S5) and the performance of existing methods is very different. Therefore adding 1-2 more datasets for evaluation is recommended

*> We agree that it would be useful to add additional datasets. Thus, we added an additional supplementary figure (namely, Figure S6), which displays the negative control results of all methods stratified by effective sample size on two additional datasets: the Frangieh (control) dataset and the simulated dataset. We also updated the main text (pg 4) as follows: “We found that effective sample size had a substantial effect on the calibration of many methods under consideration (Figure S5-S6), …”*

3. Technical factors: Figure 2d is a little but confusing as I don't quite get how differences in biological replicates affect the results? Maybe authors show the results for R1/R2/R3 separately, and in comparison with the results merging three replicates together?

*> We agree that the original version of the manuscript did not adequately explain Figure 2d. We added the following paragraph to the “Systematic identification of core analysis challenges” section (pg 4):*

*> “Second, technical factors, such as biological replicate, batch, and library size, impact not only a cell's expression level, but also its probability of receiving a perturbation, thereby creating a confounding effect that can lead to spurious associations. All existing methods adjust for library size, but few adjust for other technical factors (Table 1). We studied how the variable of biological replicate confounded the Papalexi (gene modality) data (Figure 2d). The Papalexi data were sequenced across three separate biological replicates (which we label “R1,” “R2,” and “R3”). We visually examined the relationship between biological replicate and a given NT gRNA (“NTg4") and gene (FTH1). We plotted the fraction of cells in each biological replicate that harbored ``NTg4'' (Figure 2d, left); additionally, we plotted the (library-size-normalized) expression level of FTH1 across biological replicate, superimposing boxplots indicating the 25th, 50th, and 75th percentiles of the distribution (Figure 2d, right). We observed clear visual evidence that biological replicate impacted both NTg4 presence or absence and FTH1 expression level, creating a confounding effect. For example, cells in biological replicate R1 were much more likely than cells in biological replicates R2 or R3 to contain NTg4; on the other hand, cells in biological replicate R1 exhibited a much lower expression level of FTH1 than cells in biological replicates R2 or R3. If one naively tested for association between NTg4 and FTH1 while ignoring batch, one would incorrectly conclude that NTg4 decreased the expression of FTH1. In fact, NTg4 exerted no effect on the expression FTH1, as NTg4 was a negative control gRNA.”*

*> We note that figure 4e displays the results of NB regression on the Papalexi data with and without biological replicate included as a covariate. The calibration of the method improved when biological replicate was included as a covariate, demonstrating (indirectly) that biological replicate exerted a confounding effect. We believe that this is a slightly more principled approach to carrying out the analysis that the reviewer suggests (i.e., applying a method to analyze the biological replicates separately, and then applying the same method to analyze all biological replicates together, while excluding biological replicate as a covariate).*

4. Technical factors: Fig S6 is difficult to understand. More explanation is needed to show how replicates affect the QQ plot

*> We agree that Figure S6 (now Figure S7) was challenging to interpret in the original version of the paper. We updated the caption of the figure as follows (pg 43):*

*> “We conducted a test of association between each NT gRNA and biological replicate. We plotted the resulting p-values on a QQ plot (left); each point in the plot corresponds to a different NT gRNA. Likewise, we carried out a test of association between the expression of each gene and biological replicate (right); each point in the plot similarly corresponds to a different gene. We observed that the two sets of p-values were inflated, indicating that the bulk of the NT gRNAs and the bulk of the genes was associated with biological replicate. Because biological replicate affected both gRNA presence/absence and gene expression, we reasoned that biological replicate acted as a confounder on the Papalexi data. The section ‘Details of the investigation into the core analysis challenges’ describes how the tests of association were conducted.”*

positive control data (page 7, line 51). A more description is needed here in terms of what pairs are considered positive. TSS guides are easy to understand. For enhancers: identifying enhancer-gene pairs is a very challenging task, therefore I'm not sure how to define "known" enhancers here.

*> We agree that validating enhancer-gene links is indeed challenging. Therefore, to ensure that the positive control pairs were of uniformly high-quality, we removed the enhancer-gene pairs from the set of positive control pairs, retaining only the TSS-gene (or, in the case of the Papalexi protein modality, TSS-protein) pairs. We note that the original set of positive control pairs contained 584 pairs. Of these, only 5 (or <1%) were enhancer-gene pairs. Thus, removing the enhancer-gene pairs essentially did not change the positive control results at all (Figure 4c).*

*> We updated the text as follows (pg 6): “Next, we assessed the power of the methods by applying them to positive control data. We constructed positive control pairs for each dataset by coupling perturbations targeting TSSs to the genes (or proteins) regulated by these TSSs.”*

1. A related thought is, since authors already use synthetic data, generating synthetic positive perturbation-gene pairs would be a better way for evaluating sensitivity?

*> We added a simulated positive control dataset to the set of positive control datasets that we analyzed (Figure 4c). To mirror the simulated negative control dataset, we designed the positive control dataset such that it was in an “easy” region of the problem space: there was no confounding, and the pairwise effective sample sizes were fairly large. We found that SCEPTRE tied three other methods for making the greatest number of discoveries on the simulated positive control dataset. While the simulated positive control dataset was useful for verifying that the methods were capable of making true discoveries under known ground truth, the real-data positive control analyses were more realistic.*

*> We added the following paragraph to the Methods section (pg 22) to describe generation of the synthetic positive control data: “We also generated a synthetic positive control dataset to assess the power of the competing methods under known ground truth. The synthetic positive control dataset contained 125 genes, 25 positive control gRNAs, 100 negative control gRNAs, and 15,000 cells. The mean expression of each gene across ``treatment'' and ``control'' groups was drawn (separately) from a Gamma(0.5, 2) distribution. Finally, gene-wise sizes were drawn from a Unif(1, 25) distribution. Like the negative control dataset, the positive control dataset was devoid of confounding.”*

- discovery analysis (page 9, last paragraph before Discussion). The IRF1/STAT1 analysis is rudimentary and requires further analysis. For example, how is SCEPTRE compared with other methods for identifying the enrichment of IRF1/STAT1 targets using existing ChIP-seq? Also authors mention Papalexi and Frangieh datasets are used, however only IRF1/STAT1 (from Papalexi dataset) are mentioned. How about the results in the Frangieh dataset?

*> We applied all seven methods to carry out a partial trans analysis of the Papalexi (gene modality) data, assessing the extent to which the methods produced discovery sets that were enriched for ChIP-seq signal. We described our results in the “Application of SCEPTRE for discovery analyses” section (pg 8):*

*> “To validate the results of the discovery analysis, we identified the downstream target genes of the transcription factors STAT1 and IRF1 using cell-type-relevant ChIP-seq data. We examined the degree of concordance between the discovery set produced by each method and the ChIP-seq-identified targets (Figure S8). To this end, for each method, we constructed a two-by-two contingency table of genes contained within the discovery set of the method and genes whose TSS overlapped with a ChIP-seq peak. We computed a p-value (via a Fisher exact test) on this contingency table, quantifying the extent to which the method's discovery set was enriched for ChIP-seq signal.*

*> We made several observations. First, the discovery set of the KS test did not demonstrate enrichment for ChIP-seq signal (enrichment p > 0.5), likely because the KS test produced a large number of false discoveries. Second, and somewhat surprisingly, MIMOSCA's discovery set exhibited the greatest degree of enrichment for ChIP-seq signal (among all methods) on STAT1 and the lowest degree of enrichment (among all methods, excluding the KS test) on IRF1. However, MIMOSCA made many fewer discoveries than any other method on the discovery data (in particular, 956 and 875 fewer discoveries than the next closest method for IRF1 and STAT1), a trend consistent with MIMOSCA's results on the the positive control data (Figure 4). Among the methods that made a large number of discoveries on both the positive control data and the discovery data (i.e., all methods except for MIMOSCA), SCEPTRE ranked second out of six for ChIP-seq signal enrichment on both IRF1 and STAT1, with the top method being different across the two transcription factors. Thus, SCEPTRE appeared to exhibit consistently good performance on the discovery data (and more broadly across all of our analysis; Figure 4), increasing our confidence in the results.”*

*> We additionally added a new figure (Figure S8) to display these results. We only performed a ChIP-seq analysis on the Papalexi data, as we could not locate cell-type-relevant and condition-relevant ChIP-seq data for the Frangieh data despite searching extensively.*

Minor:

- page 7, line 24: "hundreds of times faster" -- is there a figure/table to actually demonstrate the author's claims?

*> We conducted an empirical benchmarking study to compare the speed of the spectral decomposition approach to that of the standard QR decomposition approach for computing GLM score tests. We reported on this analysis in the new section “Comparing the spectral decomposition algorithm to the QR decomposition algorithm for computing GLM score tests” (pg 60). We found that that spectral decomposition approach was hundreds of times faster than the QR decomposition approach for sparse, binary treatment vectors (Table S4).*

*> We added a reference to this section on pg 6: “See Comparing the spectral decomposition algorithm to the QR decomposition algorithm for computing GLM score tests for details.”*

**Second round of review**

**Reviewer 1**

Overall, I think this is an important study, well and rigorously executed, and a valuable contribution to the field. It deserves to be published in Genome Biology. The authors have thoroughly addressed my questions.

I note as a future consideration for the authors: although I am satisfied that IWOR is a valid approach and rather elegant as a sampling strategy, I don't understand the motivation for conserving _indices_ across perturbations. The authors state, "Unfortunately, drawing B WOR samples for every perturbation-gene pair — which is required for carrying out the permutation test dataset-wide — is slow." Maybe, but it's non-obvious that it matters compared to the other operations performed per permutation. Even if it does, why not use the same permutations across all _genes_ tested for a given perturbation? This would require no special sampling approach. That said, while IWOR may be unnecessary it certainly shouldn't harm anything.

**Reviewer 2**

The revised manuscript is satisfactory for publication.

Authors addressed all my previous concerns by performing quite substantial additional analyses. I don't have any further comments. A recommendation (not a major concern) is, authors may consider plotting ROC curve for the synthetic datasets used in Figure 4. An ROC curve will better reflect the balances between true positives and false positives between different methods.
